# Scar shape analysis and simulated electrical instabilities in a non-ischemic dilated cardiomyopathy patient cohort

**DOI:** 10.1371/journal.pcbi.1007421

**Published:** 2019-10-28

**Authors:** Gabriel Balaban, Brian P. Halliday, Wenjia Bai, Bradley Porter, Carlotta Malvuccio, Pablo Lamata, Christopher A. Rinaldi, Gernot Plank, Daniel Rueckert, Sanjay K. Prasad, Martin J. Bishop

**Affiliations:** 1 Department of Informatics, University of Oslo, Oslo, Norway; 2 School of Biomedical Engineering and Imaging Sciences, King’s College London, London, United Kingdom; 3 National Heart and Lung Institute, Imperial College, London, United Kingdom; 4 Department of Computing, Imperial College, London, United Kingdom; 5 Department of Informatics, King’s College London, London, United Kingdom; 6 Department of Cardiology, Guy’s and St. Thomas Hospital Trust, London, United Kingdom; 7 Institute of Biophysics, Medical University of Graz, Graz, Austria; 8 Cardiovascular Research Centre and Cardiovascular Magnetic Resonance Unit, Royal Brompton Hospital, London, United Kingdom; University of California San Diego, UNITED STATES

## Abstract

This paper presents a morphological analysis of fibrotic scarring in non-ischemic dilated cardiomyopathy, and its relationship to electrical instabilities which underlie reentrant arrhythmias. Two dimensional electrophysiological simulation models were constructed from a set of 699 late gadolinium enhanced cardiac magnetic resonance images originating from 157 patients. Areas of late gadolinium enhancement (LGE) in each image were assigned one of 10 possible microstructures, which modelled the details of fibrotic scarring an order of magnitude below the MRI scan resolution. A simulated programmed electrical stimulation protocol tested each model for the possibility of generating either a transmural block or a transmural reentry. The outcomes of the simulations were compared against morphological LGE features extracted from the images. Models which blocked or reentered, grouped by microstructure, were significantly different from one another in myocardial-LGE interface length, number of components and entropy, but not in relative area and transmurality. With an unknown microstructure, transmurality alone was the best predictor of block, whereas a combination of interface length, transmurality and number of components was the best predictor of reentry in linear discriminant analysis.

## Author summary

Non-ischemic dilated cardiomyopathy is a disease in which the lower left chamber of the heart is abnormally large. The cause of the disease can be anything that is not a loss of blood supply to the heart. Many patients with non-ischemic dilated cardiomyopathy have scars in their hearts which can be detected with magnetic resonance imaging. These scars are thought to disrupt the flow of electricity through the heart and cause deadly rhythm disorders. In our study we look at various ways to quantify the shapes and textures of the scars with MRI images, and relate these metrics to rhythm disturbances that we simulate in image-based biophysical models of electrical activity. The exact electrical properties of scars in non-ischemic dilated cardiomyopathy are unknown, so our simulations account for a variety of possible properties. Our findings suggest new ways of examining scar shapes which work well with all of the properties we tested. This makes our shape metrics potentially valuable for determining which non-ischemic dilated cardiomyopathy patients are at risk of future heart rhythm disorders.

## Introduction

Non-ischemic cardiomyopathies are a class of cardiac pathologies in which the myocardial tissue undergoes remodelling due to causes other than compromised coronary perfusion. Due to the complex and multifactorial aetiology of non-ischemic disease, assessing the risk of cardiac arrhythmias in these patients remains a major challenge. Indeed, using current risk stratification strategies, a recent clinical trial [1] failed to show a significant reduction in mortality in the non-ischemic population with the use of implantable cardioverter-defibrillators (ICD), a common therapy for the treatment of cardiac arrhythmias.

Progress has been made in the case of non-ischemic dilated cardiomyopathy (NIDCM) in which an association has been shown between arrhythmias and the presence of fibrotic scarring, as detected by late gadolinium enhanced cardiovascular magnetic resonance imaging (LGE-CMR) [2]. Since then, further imaging studies have examined the morphology of late gadolinium enhancement (LGE) in greater detail, and its relation to arrhythmic risk. In a large cohort study, Halliday et al. [3] showed that the risk of arrhythmia increased rapidly from 0 to 4% LGE extent, but did not significantly increase afterwards, and that LGE location was important. Furthermore, several studies [4–6] have investigated the crucial role of scar transmurality, and implicated either high [4, 5], or medium range (25-75%) [6] transmurality as being an arrhythmic substrate. Finally, in athletes with non-ischemic scars, a long and thin ‘stria’ pattern of LGE has been shown to be particularly dangerous [7].

In addition to shape-based features, the myocardial and LGE texture, as imaged by LGE-CMR, has also been considered. In particular, Muthalaly et al. [8] showed a correlation of left ventricular image entropy, a measure of information disorder, with arrhythmic risk in a non-ischemic patient cohort.

Computer simulations of electrical propagation have recently shed important insights into the mechanisms underlying disrupted conduction pathways and the subsequent genesis of reentrant circuits in fibrotic patterns characteristic of non-ischemic structural heart diseases [9–11]. In our own recent work, we demonstrated the importance of scar microstructure in NIDCM [11]. More specifically, we showed that the fibrosis density, the specific texture of the fibrosis (interstitial or replacement fibrosis) and the degree of gap-junction remodelling all play a role in the formation of unidirectional block and reentry.

A key feature of non-ischemic LGE-CMR imaging is that the image intensity is only a relative, and not absolute, quantity, and so determining accurate fibrosis densities is difficult [12]. Furthermore, current clinical scan resolutions limit the amount of detail with which a region of non-ischemic fibrosis can be imaged. It is therefore vital to identify key aspects of fibrosis morphology, which may be dangerous for a range of possible microstructures, and which can be accurately assessed by non-invasive imaging. Although certain aspects of scar morphology have been investigated previously with respect to arrhythmic risk, a large-cohort mechanistic analysis has not yet been carried out.

In this study, we build upon our previous work analysing the arrhythmogenic risk associated with scar microstructure, combining it now with access to a large cohort of LGE-CMR scans of NIDCM patients with LGE [2]. This allows us to conduct a cohort based computational simulation study to examine the specific morphological features of LGE associated with high arrhythmic risk. Importantly, we are able to vary the microstructure for individual LGE patterns in the cohort, allowing us to extract robust morphological metrics that quantify arrhythmogenic patterns of LGE, and which hold independent of the scar microstructure. Finally, we also provide mechanistic insight into the underlying pathological processes associated with the formation of conduction block and reentry.

## Results

### Image characteristics

**Fig 1:**
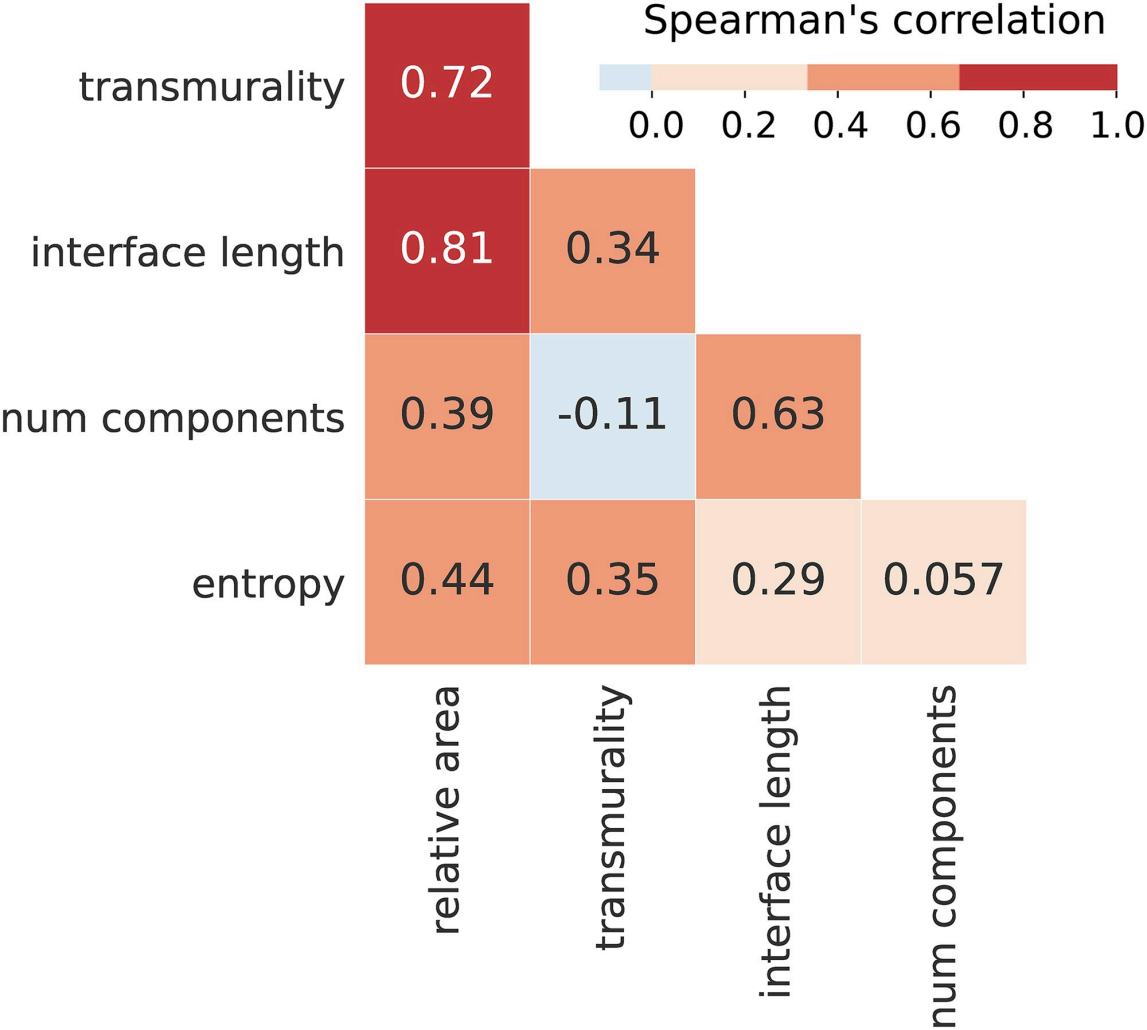
LGE feature Spearman correlation values.

We characterized each LGE-CMR image by a set of 5 features (relative area, transmurality, interface length, number of components and entropy), with the first four features being related to the LGE shape and the last to the LGE texture. The median value and interquartile range of these features is given in Table 1. The pairwise Spearman correlation values for the features are given in Fig 1. The highest correlations were observed among the features related to the size of the LGE pattern (relative area, interface length, transmurality), with the number of components and entropy having a relatively low correlation with the other features.

**Table 1:** Number of patients, images and simulations, along with LGE feature distribution given as median and IQR.

**study characteristics**		
patients	157	
images	699	
simulations	6990	
**LGE features**		
relative area	0.14	(0.07–0.25)
transmurality	0.39	(0.29–0.5)
interface length (mm)	137.5	(73.05–207.63)
number of components	7	(4–10)
entropy	2.97	(2.68–3.23)

### Simulations of block and reentry

**Fig 2:**
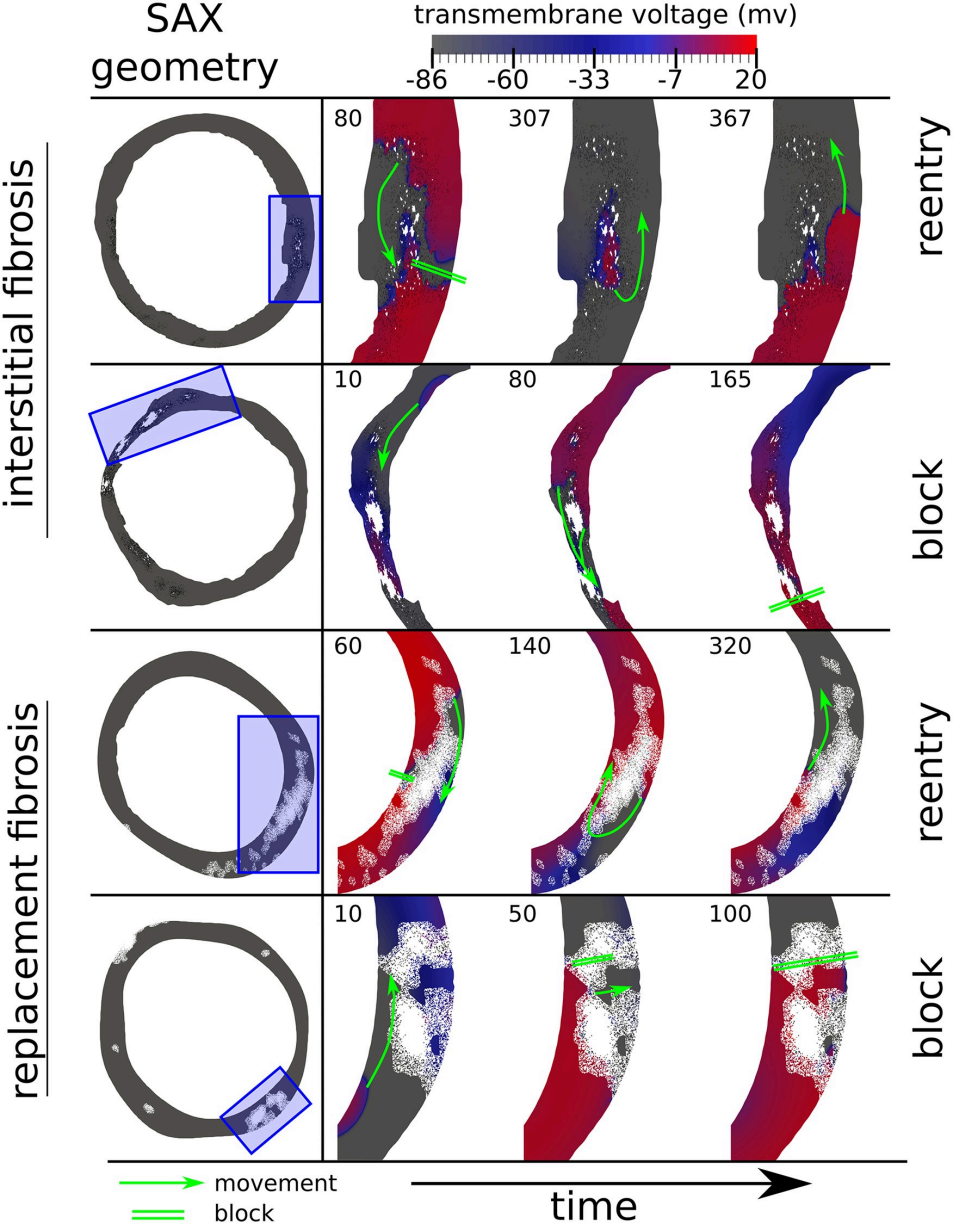
Example instances of transmural reentry and transmural block for simulation models with replacement and interstitial fibrosis based on short axis (SAX) LGE-CMR images. White areas are electrically inert, whereas black lines represent separating edges which current cannot cross.

For each LGE-CMR image we performed simulated programmed electrical stimulation using two types of fibrosis (interstitial, replacement), and 5 levels of maximum fibrosis (0.2, 0.4, 0.6, 0.8, 1.0), giving 10 different simulated fibrosis microstructures per image, and a total of 6990 simulations. Each simulation resulted in either a transmural reentry, a transmural block, or no event. Illustrative examples of transmural reentry and transmural block are given in Fig 2 as time sequence snapshots of the simulated transmembrane voltage. Example videos are given in the supplement.

### The effects of microstructure

**Fig 3:**
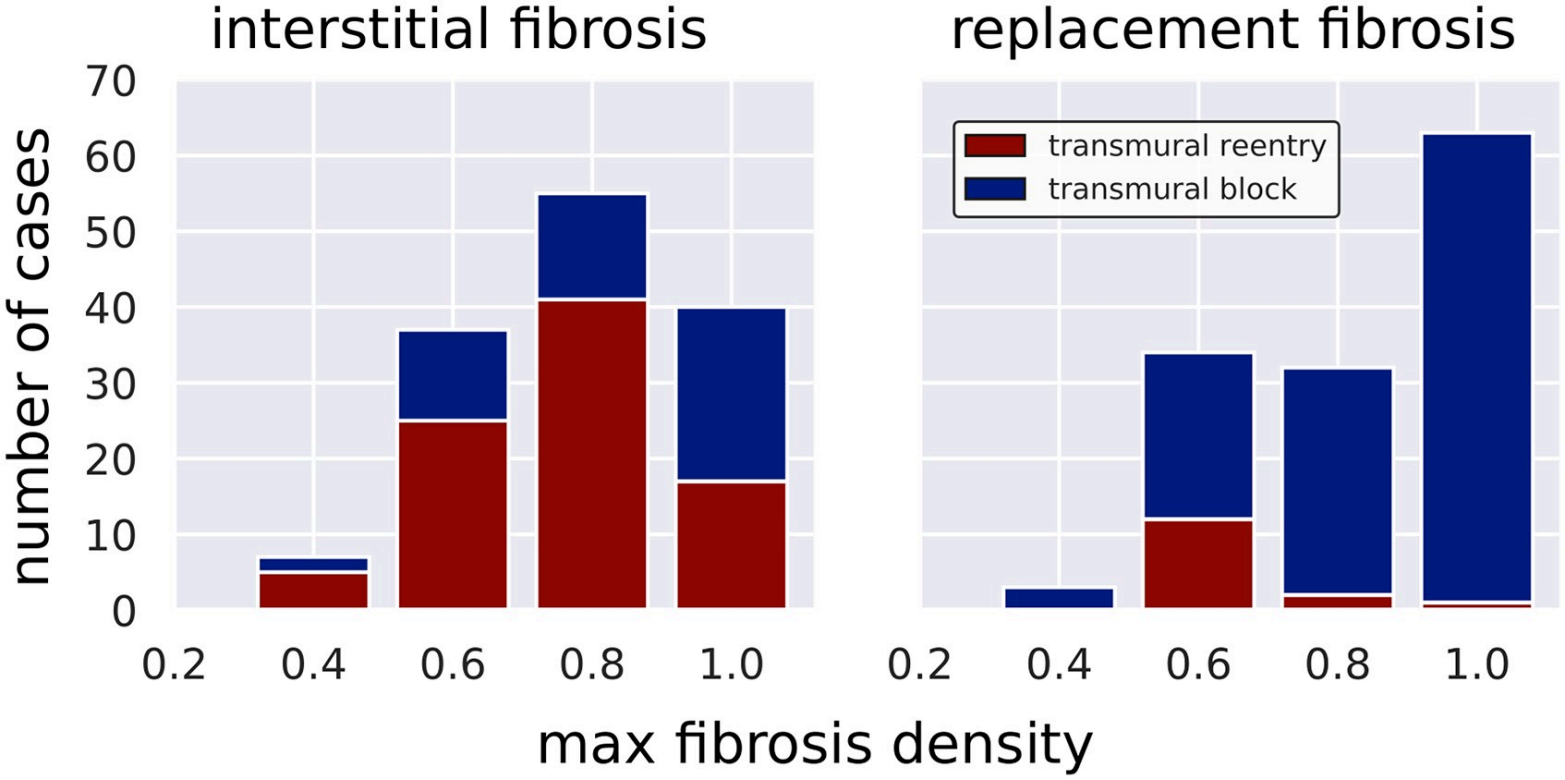
Number of simulated electrical instabilities (transmural block or transmural reentry) observed in 699 LGE-CMR derived simulation models for each combination of fibrosis type and density.

The number of occurrences of transmural block and transmural reentry for each microstructure are given in Fig 3. Blocks and reentries occurred at the maximum fibrosis density 0.4 and above. Transmural block was more prevalent with replacement fibrosis (117 replacement vs. 51 interstitial, P < 0.01) while transmural reentry was more prevalent with interstitial fibrosis (15 replacement vs. 88 interstitial, P < 0.01).

We investigated the role of microstructure in determining which LGE patterns experienced a simulated block or reentry. This was facilitated by aggregating the microstructures into four microstructure types: interstitial high density, interstitial low density, replacement high density, and replacement low density. The high and low density microstructure types contained models with maximum fibrosis density ranges 1.0-0.8 and 0.6-0.4 respectively.

**Fig 4:**
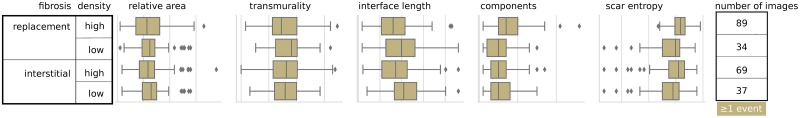
Distribution of scar feature values for various microstructure types. Microstructure types are classified as interstitial or replacement, and high (max density 0.8-1.0) or low (max density 0.4-0.6) density. Each data point represents an image whose models experienced at least one event (reentry or block) for a particular microstructure type.

For each of the four microstructure types we plotted the distribution of the scar feature values among the images whose models experienced at least one block or reentry. These data are shown in Fig 4, and showed no significant differences for relative area and transmurality. However, significant differences existed for interface length, components and entropy. For both the replacement and interstitial fibrosis types the high density groups had higher scar entropy (replacement *P* < 0.01, interstitial *P* = 0.02), and lower interface length (replacement *P* = 0.02, interstitial *P* = 0.01), than the low density groups. Finally, the high density replacement microstructure had a greater number of components than the other microstructures (*P* < 0.01).

We also carried out our microstructural analysis with the outcome split into transmural reentry and transmural block. We found trends in transmurality, interface length, and scar area which did not quite reach statistical significance. This analysis is included in the supplement S1 Fig.

### Prediction of block or reentry using a single feature

Now that we have considered the effects of different microstructures, we move onto the clinically relevant problem of identifying a potentially dangerous LGE pattern without any knowledge of the microstructure, which cannot be viewed via current medical imaging technologies.

**Fig 5:**
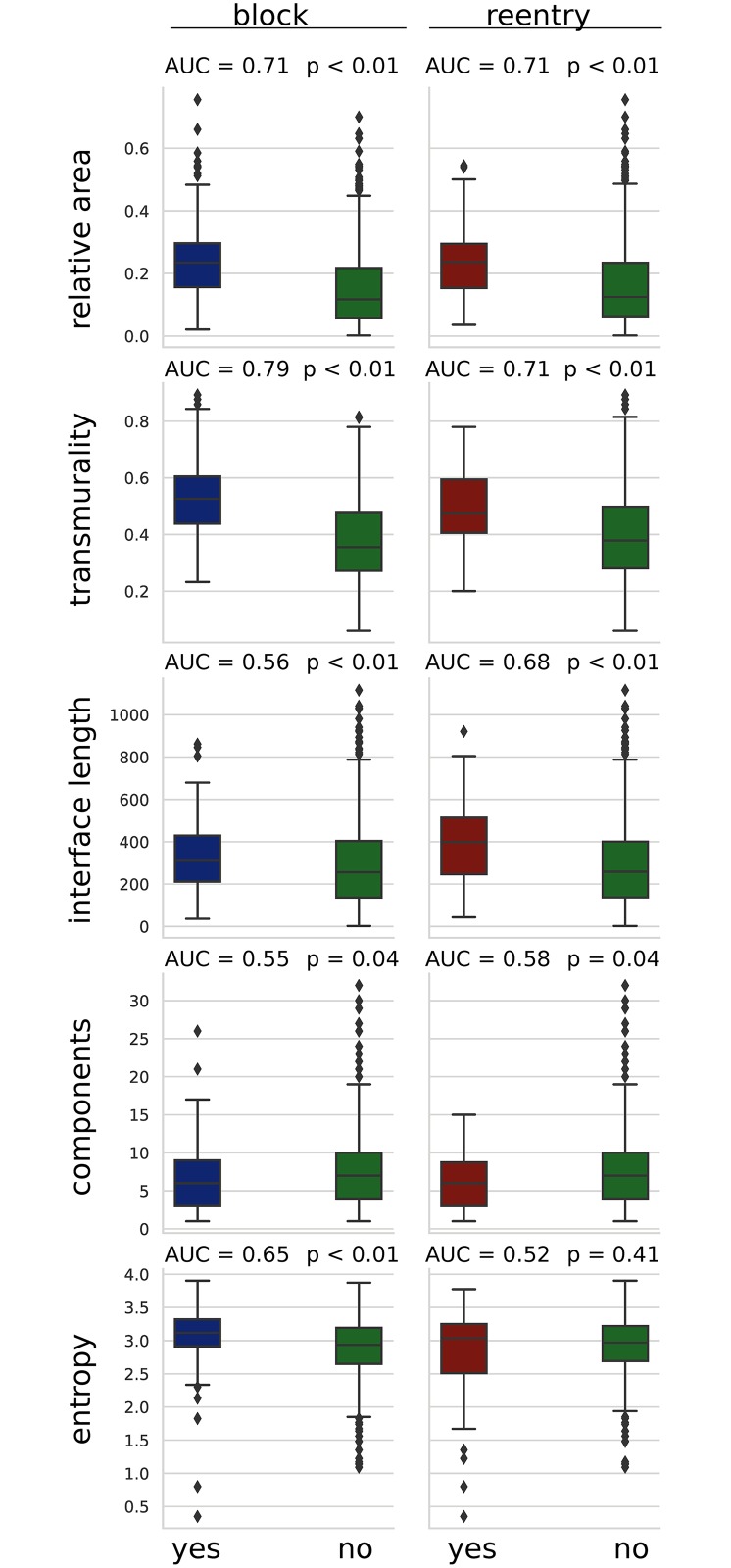
Boxplots showing the distribution of image feature values for images that had at least one simulated block vs no block (right column) or at least one simulated reentry vs no reentry (left column). AUC scores are area under curve in a receiver operating curve analysis for each feature’s ability to predict either reentry or block for a given image.

We compared the distribution of scar feature values to our simulated block and reentry results. All images that experienced transmural block in any of the simulated microstructures were compared to those that did not, with a similar division being made for reentry. The results of these comparisons are given in Fig 5. The relative area, transmurality, and interface length were all significantly higher in the block and reentry image groups (P < 0.01). The number of components was lower in both block and reentry groups (P = 0.04), whereas the entropy was only significantly greater in the block group (P < 0.01).

There were two threshold effects present in the image feature data. No blocks or reentries were observed in any image with a transmurality of less than 0.2, and no reentries were observed in images whose LGE was broken into more than 15 components.

We ranked the ability of each feature to discriminate images that experienced block or reentry by considering the area under the receiver operating characteristic curve (AUC). In the case of block the ranking was (1. transmurality, 2. relative area, 3. entropy, 4. interface length, 5. number of components) with AUC values of (0.79, 0.71, 0.65, 0.56, 0.55) respectively. In the case of reentry the ranking was (1. relative area and transmurality, 2. interface length, 3. number of components, 4. entropy) with respective AUC values of (0.71, 0.68, 0.58 and 0.52).

### Predictive capability of feature combinations

We used linear discriminant analysis (LDA) to compute linear combinations of features with the aim of improving the predictive capability of detecting a potential transmural block or reentry in an image. We found that using combinations of 2-3 features improved the AUC for reentry, but not block, and that combinations of 4 or more features did not improve the AUC any better than combinations of 2-3 features.

The top 3 combinations of 2-3 features are given in Table 2. In the case of block the AUC values of the feature combinations were not better than that achieved by using transmurality alone. For predicting reentry, we found that the LDA combinations were able to provide more accurate predictions than using any single feature. In particular, the combination of transmurality, interface length and components achieved an AUC of 0.8. This compares favourably with the 0.71 obtained by using transmurality or relative area alone, which were the most predictive features for reentry in the univariate analysis.

**Table 2:** The top 3 best combinations of 2-3 LGE features obtained from Fisher’s linear discriminant analysis (LDA) for predicting block or reentry in one more simulations for a given image. All features were max-min normalized to the range 0-1. Coefficients are the weight values assigned by the LDA to each normalized feature. AUC values are area under the curve from a 20-fold cross validated receiver operating curve analysis whose threshold values (obtained from 1 fold) are the feature linear combinations (obtained from the remaining 19 folds).

event	features	LDA coefficients	AUC
block	transmurality, entropy	6.0, 0.44	0.78
	transmurality, components	6.05, -0.55	0.78
	transmurality, interface length	6.27, -0.62	0.78
reentry	interface length, components, transmurality	7.17, -6.45, 1.28	0.80
	interface length, components	7.9, -7.09	0.79
	interface length, components relative area	8.58, -7.32, -0.74	0.79

The coefficients for the LDA combinations are also given in Table 2. Since we max-min normalized all of the features before the LDA, the size of the coefficients give the relative importance of each variable to the prediction. In particular, in the best combination for reentry prediction the interface length coefficient is almost 6 times greater than the coefficient for transmurality, indicating the greater importance of interface length. Furthermore, the signs of the transmurality and interface length coefficients are both positive, so that these features work together to improve the prediction accuracy for reentry, albeit by a modest 1 point gain in AUC over interface length and components. Such an improvement was not realized by any other combination of features related to scar size (relative area, transmurality, interface length). This is expected since the correlation between transmurality and interface length is relatively low (0.34), and is much higher between the other size features.

## Discussion

We investigated the role of scar microstructure and LGE morphology in the formation of transmural block and reentry in the context of non-ischemic dilated cardiomyopathy. We found that the microstructure underlying the LGE influenced the morphological characteristics of the LGE patterns which blocked or reentered. Furthermore, we tested the value of morphological LGE features for predicting reentry or block when the microstructure was unknown. Our results suggest that transmurality alone is the best predictor of an LGE pattern that has the potential for transmural block, and that a combination of interface length, number of components and transmurality is the best predictor of reentry.

### Mechanisms of block and reentry

Ex-vivo studies of non-ischemic fibrosis in myocardial tissue have shown a slowing of conduction velocity in fibrotic areas [13, 14]. This is most likely due to the fibrosis itself acting as a barrier to conduction [13], and to reduced conductivity in remodelled myocytes [14, 15].

In our simulations, this slowed conduction was exacerbated by pacing at the effective refractory period (ERP), resulting in zones of transient conduction block which restricted electrical propagation into increasingly narrow and convoluted pathways. When a signal from one of these narrow pathways met a relatively large area of unactivated tissue, propagation could fail due to a source-sink mismatch. Such pathways could then be reentered at a later time when excitability was recovered. These mechanisms are similar to what has been noted in simulations of idealized fibrosis [16], atrial fibrosis [17], as well as in our previous study of fibrosis microstructure in NIDCM [11]. For the interstitial fibrosis, the reentry distribution followed a biphasic pattern with a peak at the maximum density 0.8, which is consistent with previous studies [11, 16, 17].

### The effects of fibrosis microstructure and morphology

A novelty in our current study is that we have examined the contribution of both fibrosis micro- and macro-structure to the formation of reentry in NIDCM. Our results indicate that transmural block most often occurs with highly transmural scars, and for replacement fibrosis with high density (0.8-1.0). Such microstructures have a very high volume fraction of fibrosis. This severely restricts propagation and acts together with the high fibrosis transmurality to make transmural conduction failure more likely.

Furthermore, we observed differences in the microstructures with regards to block vs reentry prevalence. The relative percentage of images that experienced one or more reentries vs. images that experienced either one or more reentry or block was 3%, 35%, 67%, 70% for the replacement high density, replacement low density, interstitial high density, and interstitial low density fibrosis groups (supplementary S1 Fig). This ordering can be explained in terms of the relative electrical permeability of the scars. As scars become more permeable they are less likely to block completely, thereby favouring transmural reentry events rather than complete conduction block. Higher density scars are less permeable than lower density scars, and interstitial fibrosis is more permeable than replacement fibrosis due to relatively unhindered longitudinal conduction.

A key aspect of the transmural reentries that we simulated was a long and torturous conduction path through the fibrosis. Such a path allowed neighbouring healthy tissues sufficient time to recover so that they could be reactivated by waves exiting the fibrosis. We showed that the fibrosis transmurality played a key role in such a scenario, most likely because greater transmurality allowed for longer intra-fibrosis activation paths. Indeed, we noted a minimum transmurality of 0.2 for any reentry or block to occur. The connectivity was also important, with disconnected scars containing shorter internal activation paths which reduced the chance of reentry.

In the best LDA combination for predicting reentry, interface length played a large role. A longer interface length most likely extended the distance (and therefore activation time) between entrance and exit sites of signals meeting fibrosis via healthy tissue. This made it more likely for signals exiting fibrosis to meet tissue which had recovered exitability, thereby creating a reentry.

Two recent LGE-CMR studies have examined the role of entropy in NIDCM. Muthalaly et al. [8] measured entropy across the entire myocardium (LV entropy) and concluded that high LV entropy is a predictor of future arrhythmic events. Using a slightly different method which focused on LGE only, Gould et al. [18] showed no correlation between scar entropy and arrhythmic outcome in non-ischemic patients.

Mechanistically, a high entropy value means greater variation in the local microstructure, which may exacerbate source-sink mismatches and make conduction block and therefore reentry more likely. This is supported by our results for transmural block, as well as by Kazbanov et al. [10]. Nevertheless, the lack of a significant difference in entropy in our reentrant LGE patterns suggests that other factors may be more important, or that the effects of entropy are more significant outside of the LGE, a scenario which our study does not investigate. More research in this area is required.

### Clinical implications

In our study we introduce the novel concept of interface length for the detection of arrhythmogenic non-ischemic scars with LGE-CMR imaging. Our results suggest that interface length is best considered together with transmurality and connectedness. More specifically, an LGE pattern with sufficient transmurality, long interface length, and high connectedness (i.e. few components) is a potential substrate for reentrant ventricular arrhythmia. This corresponds to the ‘stria’ pattern which was shown to be dangerous in athletes by Zorzi et al. [7]. By quantifying LGE patterns in a set of morphological features, we allow for the automated detection of such stria patterns by machine learning algorithms, which we expect will play a greater role in medicine in the future.

Furthermore, our microstructural analysis suggests that stria scars are particularly dangerous when the fibrosis density is intermediate (0.4-0.6 maximum density in our study), and with a high degree of anisotropy (e.g. interstitial fibrosis). This is also supported by experimental [19] and clinical [20] evidence. Such microstructures consist of a mix of myocardium and fibrosis, and may appear as areas of “patchy” intensity in LGE-CMR. The danger of such patchy fibrosis is corroborated by the catheter ablation study of Sasaki et al. [5], which showed a relationship between patchy LGE and ventricular tachycardia, as well as by Vandersickel et al. [21] who demonstrated that patchy fibrosis can attract and sustain spiral wave reentries.

### Limitations

The most significant limitation of our study was that simulations were performed in 2-D. This was done to reduce the computational cost, which allowed for large scale testing of microstructure variations in a large patient cohort. However, the fibrosis representation methods underlying our reentry simulations have been shown to extend to 3-D [22] and all of our scar metrics have natural extensions to 3-D imaging. By separating our simulation outcomes into ‘transmural reentry’ and ‘transmural block’ we aimed to make our results more relevant to the 3-D setting. In the case of ‘transmural reentry’ we envisioned that the same mechanism could occur in 3-D as well. However, for our simulations of ‘transmural block’, we felt that the reentries simulated in 2-D might be affected by more complex wavefront interactions in 3-D. This is because the reentrant waves in the ‘transmural block’ group traveled through tissue which had been refractory for some time, which could lead to collisions with other waves in 3-D. In contrast to this the reentrant waves in the ‘transmural reentry’ simulations traveled back through tissue which had been recently activated, making interactions with other waves in 3-D less likely. We have therefore chosen the label ‘transmural block’, as we believe the block proceeding the reentry is feasible in 3-D for these cases. A future 3-D extension of our study could help to clarify these issues with 2-D simulations.

A further limitation is that we only considered the initiation and not the sustenance of reentries. Indeed most of the transmural reentries that we simulated terminated after circa 2 cycles. In 3-D the presence of additional pathways may allow for longer sustenance of reentries, and this issue remains to be studied.

The in-plane resolution of our LGE-CMR images varied from 0.64-1.97 mm. As a consequence, our simulated microstructures contained heterogeneities with block sizes corresponding to the image resolution. Such differences in block-size have been shown to have an effect on reentry probability [10], and hence could have influenced the results of our simulated programmed electrical stimulation. However, we believe that image resolution did not cause any significant bias in our scar metrics, as the Spearman correlation between the in-plane resolution and all of the scar metrics was less than 0.15.

Scars identified using LGE-CMR and clinical scan resolutions have been shown to differ from those identified ex-vivo at higher resolution [23] and in histology [12]. This was not an issue for our study as we compared scar metrics to simulation results derived from models with no scar identification uncertainty. However, any future comparison of scar metrics in LGE-CMR to clinical arrhythmias will have to contend with uncertainties in scar identification.

Due to the general paucity of data available regarding the electrical properties of non-ischemic fibrosis, it was necessary to make a number of modelling assumptions. Firstly, we modelled conductivity inside the LGE as reduced, using conductivity values based on the data of Anderson et al. [13]. This assumption is reasonable given the evidence for gap junction protein remodelling in myocytes proximal to fibrosis [14, 15]. This effect can contribute to conduction slowing in tandem with the disconnections caused by interstitial and or replacement fibrosis. However, the exact conductivity values of non-ischemic fibrosis and their relation to LGE-CMR intensity are unknown. For the sake of simplicity, we used 4 sets of conductivity values to represent the observations of the Anderson et al. study. However, more gradual changes in conductivity could also be considered, given that LGE intensity (and possibly any fibrosis mediated CV slowing), is proportional to the relative amount of fibrosis in a pixel [24].

In our previous study, altering the conductivity values inside the fibrotic zone had an effect on the reentry susceptibility [11], so that we expect any changes in conductivity to affect the reentry susceptibility in the current study as well.

We also assumed membrane kinetics in enhanced and non-enhanced areas based on the ten-Tusscher 2006 model [25], and we did not account for epi-endocardial differences in cellular properties or transmural variation in fibre helix angle. Whether or not these assumptions are appropriate for fibrotic tissue in NIDCM is an open question.

Finally, we only tested one pacing location per image, though we have previously shown that reentry susceptibility is affected by pacing location [11]. This was done to reduce the number of simulations. By employing an automated procedure we have created similar pacing locations in our images, thereby ensuring consistency in pacing location effects.

## Materials and methods

### Ethics statement

Approval for patient data collection was given by the UK National Research Ethics Committee [07/H0708/83, 09/H0504/104], and all patients gave written informed consent in accordance with the Declaration of Helsinki.

### Image aquisition, selection, and segmentation

LGE-CMR images from a cohort of 157 patients referred to Royal Brompton Hospital with NIDCM and mid-wall LGE were acquired, using a previously described protocol [2]. The scanning resolution of the images was 0.64 -1.97 mm in plane with a 7-10.5 mm slice thickness. A set of images from 10 patients are included in the supplement S1 Dataset. All other images are available in anonymised form upon request. Appropriate institutional data transfer agreements will be required. Requests should be made, along with an analysis proposal, via email to the Research and Development team at Royal Brompton and Harefield NHS Foundation Trust.

Images were included in this study if they were located below the outflow tract with LGE and had a ring of myocardium thick enough to transmit a simulated electrical wave at 200 ms cycle length, the minimum used in our study. This minimum cycle length was chosen to eliminate nonclinical forms of arrhythmia which are inducible at short cycle lengths [26]. Of 829 available images 699 were included and 130 were excluded, with the majority of exclusions due to the presence of the outflow tract.

Areas of fibrosis were identified by 2 independent expert readers. Fibrosis was only considered present if areas of LGE were primarily located in the intramural and/or subepi-cardial layers, and visible in both phase-encoding directions and in 2 orthogonal views. The borders of the myocardium were delineated in each short axis slice containing fibrosis. The fibrotic areas were then segmented by expert operators using a full-width at half maximum technique and semiautomated software (CMR42, Circle Cardiovascular Imaging Inc).

### Scar shape and texture analysis

Two dimensional patterns of LGE were quantified in a set of features in each short axis LGE-CMR image. These features were based on measurements of the relative scar area, transmurality, LGE-myocardial interface length, number of components and LGE entropy. The code for this analysis is freely available at https://github.com/GabrielBalabanResearch/lgemri_scar_metrics.

The *relative scar area* was measured as the ratio of enhanced to non-enhanced myocardial pixels. The scar *transmurality* was determined by a ray tracing method, using 580 rays emanating from the central pixel of the blood pool, and the formula

$$\begin{array}{c} \text{transmurality}=\frac{1}{\#|R|}\sum_{r\in R}\frac{r_{LGE}}{r_{myo}}, \end{array}$$ (1)

where R is the set of rays that intersect any LGE, and #|R| the number of such rays. Furthermore, *r*_*LGE*_ is the number of pixels in ray *r* that intersect LGE and *r*_*myo*_ the number of the ray’s pixels contained within the myocardium. An example image demonstrating the ray tracing method is included in the supplement S2 Fig.

The *interface length* was measured by extracting all borders between the myocardium and LGE and adding together the borders’ arclength. The *number of components* was measured as the number of 4-connected areas of LGE present in an image. An LGE pattern that had a high components value was broken into more pieces than one with a lower value.

Finally the *scar entropy* was measured by the standard Shannon entropy

$$\begin{array}{c} \text{entropy}=-\sum_{i\in I}P_{i}\ln P_{i} \end{array}$$ (2)

where I is the set of image intensities present in the LGE and *P*_*i*_ the LGE area fraction occupied by intensity *i*. This gave a metric which quantified the amount of disorder present in the LGE.

**Fig 6:**
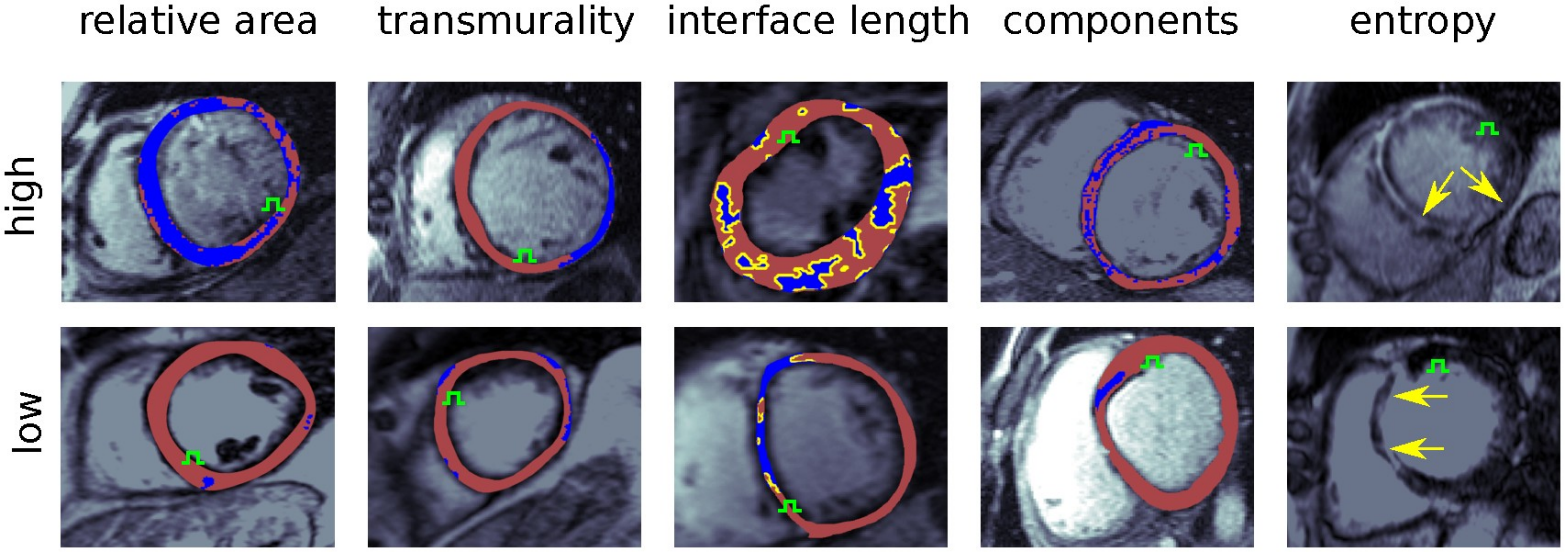
Example LGE-CMR images demonstrating high and low feature values. Areas in blue are LGE, whereas the yellow border in the interface length column shows the location of the myocardial-LGE interface. The green symbols show the stimulus sites. For the entropy images the segmentation masks are not shown to allow for visual comparison of the LGE texture.

A summary of all feature values (median, interquartile range) is given in Table 1, with inter-feature correlation given in Fig 1. Example images demonstrating high and low feature values are shown in Fig 6.

### Computational modelling

A finite element model of each image was made for the purposes of running electrophysiology simulations. This was accomplished by making a triangular mesh (CGAL) of the myocardium (max 250 *μm* edge length) using each images’ epi, endo and LGE contours [11]. Myofibre orientations were aligned with the local circumferential direction using a 2-D version of a rule based method [27].

#### Electrophysiology

The monodomain representation was used to simulate electrical activity, with cellular kinetics given by the 2006 Ten-Tusscher model [25] of the human ventricular action potential, integrated with step size 20 *μ*s. Both monodomain and cellular kinetics were implemented in the software package CARP [17]. Conductivities were tuned to match experimentally observed conduction velocities [13]. In the non-LGE areas fibre conduction velocity (CVF) was 84 cm/s and transverse conduction velocity (CVT) was 23 cm/s. LGE areas were assigned reduced conductivities as in our previous study [11], that is regions in the intensity range 0-25% and 25-50% above threshold had CVT reduced by 25% and 50% respectively, with normal CVF. Regions in the intensity ranges 50-75% and 75-100% above threshold had CVF reduced by 25% and 50% respectively, and CVT reduced by 50%.

#### Fibrosis microstructures

Random fibrosis microstructures were assigned to model LGE areas based on the local normalized image intensity

$$\begin{array}{c} I^{*}=\frac{I-I_{ref}}{I_{max}-I_{ref}}, \end{array}$$ (3)

with *I*, *I*_*max*_, *I*_*ref*_ denoting the local, maximum, and mean non-LGE reference image intensities respectively.

Both replacement and interstitial fibrosis were modelled. Replacement fibrosis was implemented by randomly removing elements with probability

$$\begin{array}{c} p_{replacement}=\alpha I^{*}, \end{array}$$ (4)

with *α* being the global maximum fibrosis level *α* ∈ [0.4, 0.6, 0.8, 1.0] which was systematically varied to create models with differing densities of fibrosis.

Interstitial fibrosis was modelled as a network of random fibrotic clefts [28], implemented by doubling mesh verticies across fibrotic edges in order to create a local no-flux boundary between triangles. The probability of a triangle edge being fibrotic was

$$\begin{array}{c} p_{interstitial}=\alpha \cos^{4}\left(\phi \right)I^{*}, \end{array}$$ (5)

where *ϕ* is the angle between the triangle edge and the local myocardial fibre direction. Triangles which were electrically isolated by a circuit of interstitial fibrosis were removed from the mesh.

### Pacing locations

Due to the large number of images considered in this study, we employed an automated procedure to determine a consistent electrical pacing location for each image. This was accomplished by choosing the minimum value of a pacing location function which we designed with the following requirements in mind:

1. The pacing locations should be consistent in their distance from the largest LGE component, which is where we expected reentries to occur.
2. The pacing locations should be endocardial as endocardial access is common in clinical electrophysiology studies, and ectopic beats often originate near Purkinje fiber terminals on the endocardium.
3. The pacing locations should allow for the simulated programmed electrical stimulation algorithm to determine the ERP properly in all simulations without interference from wavefronts lingering in fibrosis.

As these three requirements were sometimes difficult to meet in the same image, we experimented with different pacing location functions and ran test simulations until we were satisfied that requirements 1 and 2 were balanced, and that requirement 3 was always fulfilled. The resulting pacing location function

$$\begin{array}{c} \psi =\psi_{1LGE}+\psi_{endo}+\psi_{2LGE}, \end{array}$$ (6)

attempted to find an endocardial location 10 mm away from the largest LGE component which was never closer than 2mm from any LGE. Each of the three terms in the equation corresponded to a requirement, with the first term given by

$$\begin{array}{c} \psi_{1LGE}=\{\begin{array}{cc} |D_{lcLGE}-5|/10 & ifD_{lcLGE}<10\\ 1 & else \end{array} \end{array}$$ (7)

where *D*_*lcLGE*_ is the distance in mm to the largest LGE component.

The second term *ψ*_*endo*_ was defined as the transmural distance to the epicardium, obtained by solving an image Laplace equation with value 0 on the endocardium and 1 on the epicardium.

Finally the third term *ψ*_2*LGE*_ was given by

$$\begin{array}{c} \psi_{2LGE}=\{\begin{array}{cc} 0 & D_{LGE}>2\\ 2 & else \end{array} \end{array}$$ (8)

where *D*_*LGE*_ is the shortest distance in mm to any LGE. Both *D*_*lcLGE*_ and *D*_*LGE*_ were obtained via the Euclidean distance transform as implemented in the package SciPy. Example pacing locations obtained by minimizing the pacing location function are shown in Fig 6.

### Simulated programmed electrical stimulation

A simulated dynamic stimulation protocol was used to test for the possibility of each 2-D model to initiate either a transmural block or reentry. The protocol consisted of a preconditioning cycle of 3 beats at 500 ms intervals, followed by up to 5 beats with dynamically determined intervals. The timing of the dynamic beats was determined by algorithmically finding the local ERP using a binary search starting with the intervals (200 ms, 400 ms) and ending when two consecutive timings were found such that the second one initiated a new wave of activation whereas the previous one did not. A new activation wave was detected by the presence of any activations (transmembrane voltage *v*_*m*_ crossed 0 and $\frac{dv_{m}}{dt}>0$) at 8-20 ms after stimulation within 5 mm of the stimulus site.

After each dynamic beat 600 ms of electrical activity were simulated and an event was determined if any activations were present after 170 ms within 5 mm of the simulus site. Further classification of the event into a block or reentry was carried out by visual inspection of a video of the transmembrane voltage dynamics. An event was classified as ‘transmural reentry’ if the stimulus site was reentered by a wave which reversed direction, and as ‘transmural block’ otherwise. In the case of ‘transmural block’ the reactivation of the stimulus site always occurred due to propagation failure in one direction along the myocardial ring (transmural block) and a reactivation of the stimulus site by a wave traveling in the second direction. This reactivation was a potential consequence of the ring shape of the 2-D short axis geometry and might not occur in the 3-D whole organ setting. We have therefore chosen to ignore the reentry in this case and have chosen to interpret the event as ‘transmural block’.

### Statistics

Continuous variables were compared in groups by the Kruskal-Wallis test, and pairwise by the Mann-Whitney U test. The Fischer exact test was used for categorical variables. All comparisons were performed with one data point per image, to avoid a dependence of dataset size on the number of simulations. A P-value less than 0.05 was deemed significant. The mean AUC with 20 fold cross-validation was used to rank the predictive value of individual LGE features, and feature combinations determined by linear discriminant analysis.

Box and whisker plots were used to show data distributions. Boxes represent the inter-quartile range (IQR) and the horizontal line represents the median value. Whiskers represent all data within 1.5 IQR below or above the lower or upper quartile respectively. Individual points represent outliers.

## Supporting information

S1 Dataset An example set of 10 LGE-MRI patient scans and their segmentations into myocardium and enhanced (scar) areas. (ZIP)

S1 Fig An expanded version of Fig 4 with data for images that experienced at least one block and for images that experienced one reentry plotted separately in the bottom row. Images associated with least one reentry had a higher scar relative area (*P* = 0.02), higher transmurality (*P* = 0.02), and greater interface length (*P* = 0.03) than those that were associated with at least one block. For the high density interstitial microstructure, the interface length was larger (*P* = 0.02) and the transmurality smaller (*P* = 0.02), for the images associated with at least one reentry vs. those associated with at least one block. These trends, though interesting, should be viewed with caution due to the high number of comparisons (n = 20), which reduce the statistical significance after a Bonferonni correction (corrected *α* = 0.0025 assuming an original *α* = 0.05).
(TIF)

S2 Fig Demonstration of the ray tracing method used to calculate the scar transmurality. Myocardium is red, enhanced areas are blue, and rays are drawn in yellow. The ratio of enhanced to myocardial (including enchanced) pixels gives the transmurality along a ray. Note that the number of rays has been reduced to 30 in this image in order to demonstrate the method.
(TIF)

S1 Video Transmural block with simulated interstitial fibrosis. (OGV)

S2 Video Reentry with simulated interstitial fibrosis. (OGV)

S3 Video Transmural block with simulated replacement fibrosis. (OGV)

S4 Video Transmural reentry with simulated replacement fibrosis. (OGV)
