# Supplementary figures and images for: Scar shape analysis and simulated electrical instabilities in a non-ischemic dilated cardiomyopathy patient cohort
Source: PLoS Comput Biol. 2019 Oct 28;15(10):e1007421. doi: 10.1371/journal.pcbi.1007421 (PMC6837623; doi:10.1371/journal.pcbi.1007421)

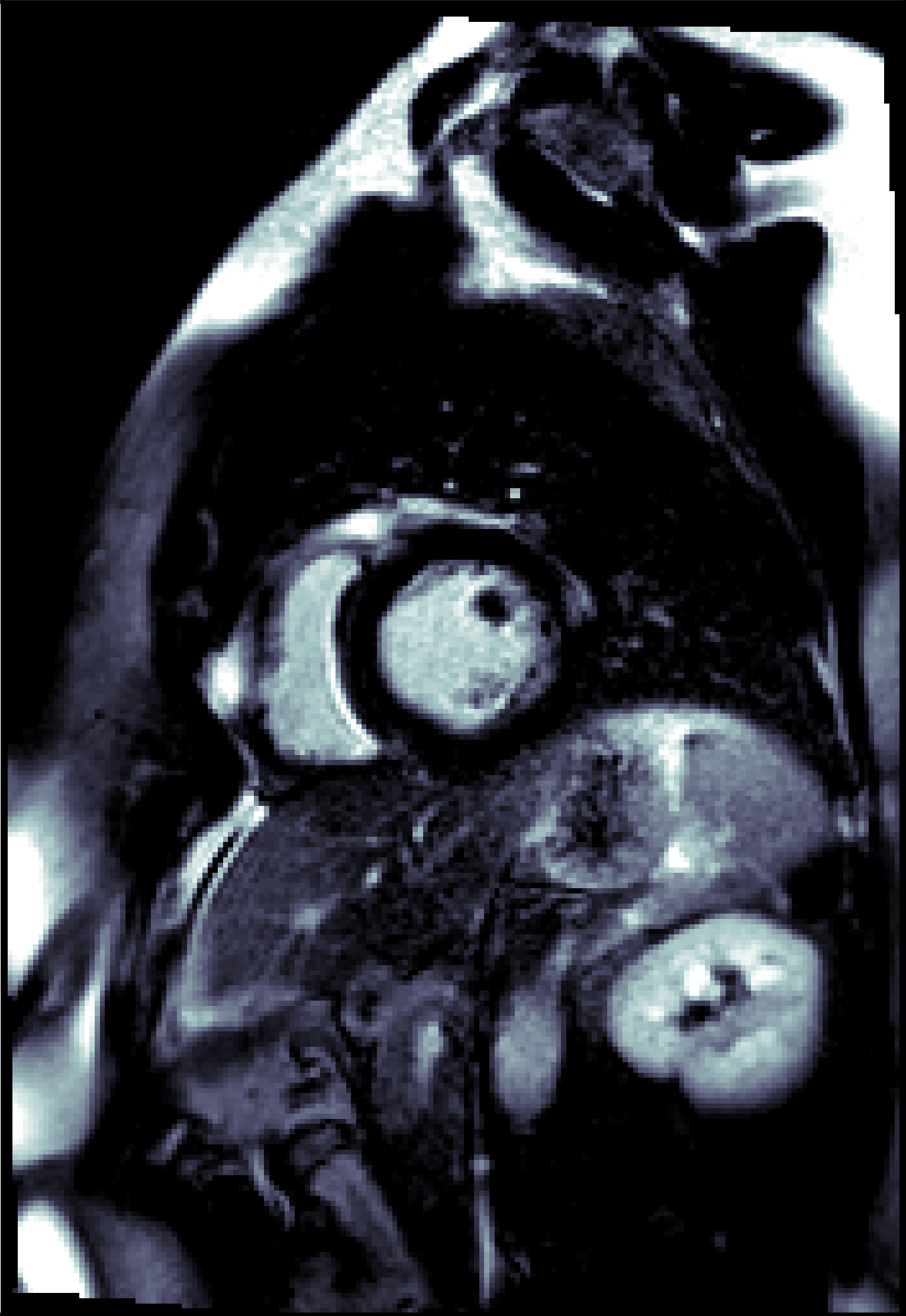

Supplement: S1 Dataset — (ZIP) [file pcbi.1007421.s001.zip › supplementary_segmented_lgemri_data/raw_data/10_00409/49_ROW_16000101124615.png]

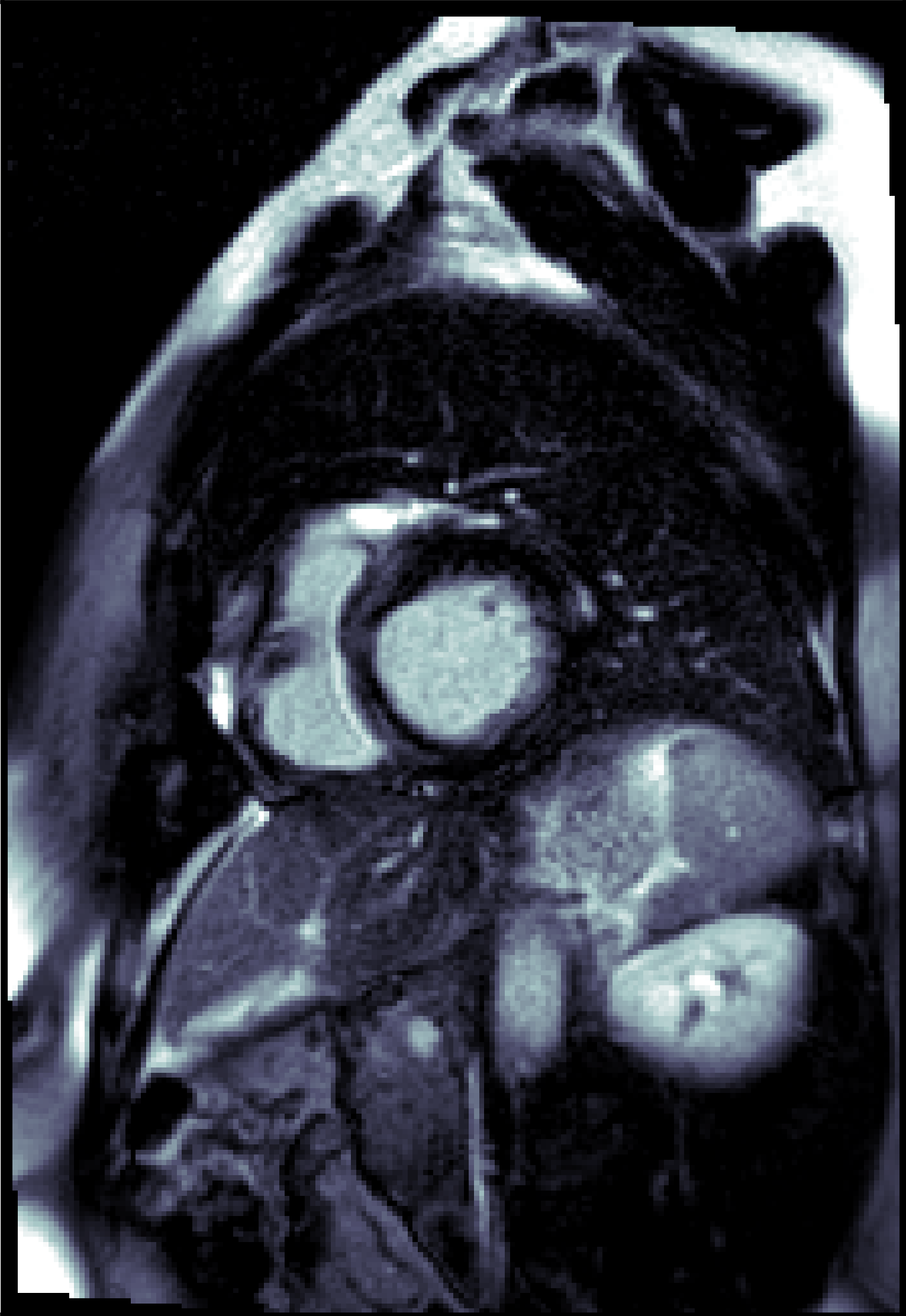

Supplement: S1 Dataset — (ZIP) [file pcbi.1007421.s001.zip › supplementary_segmented_lgemri_data/raw_data/10_00409/39_ROW_16000101124611.png]

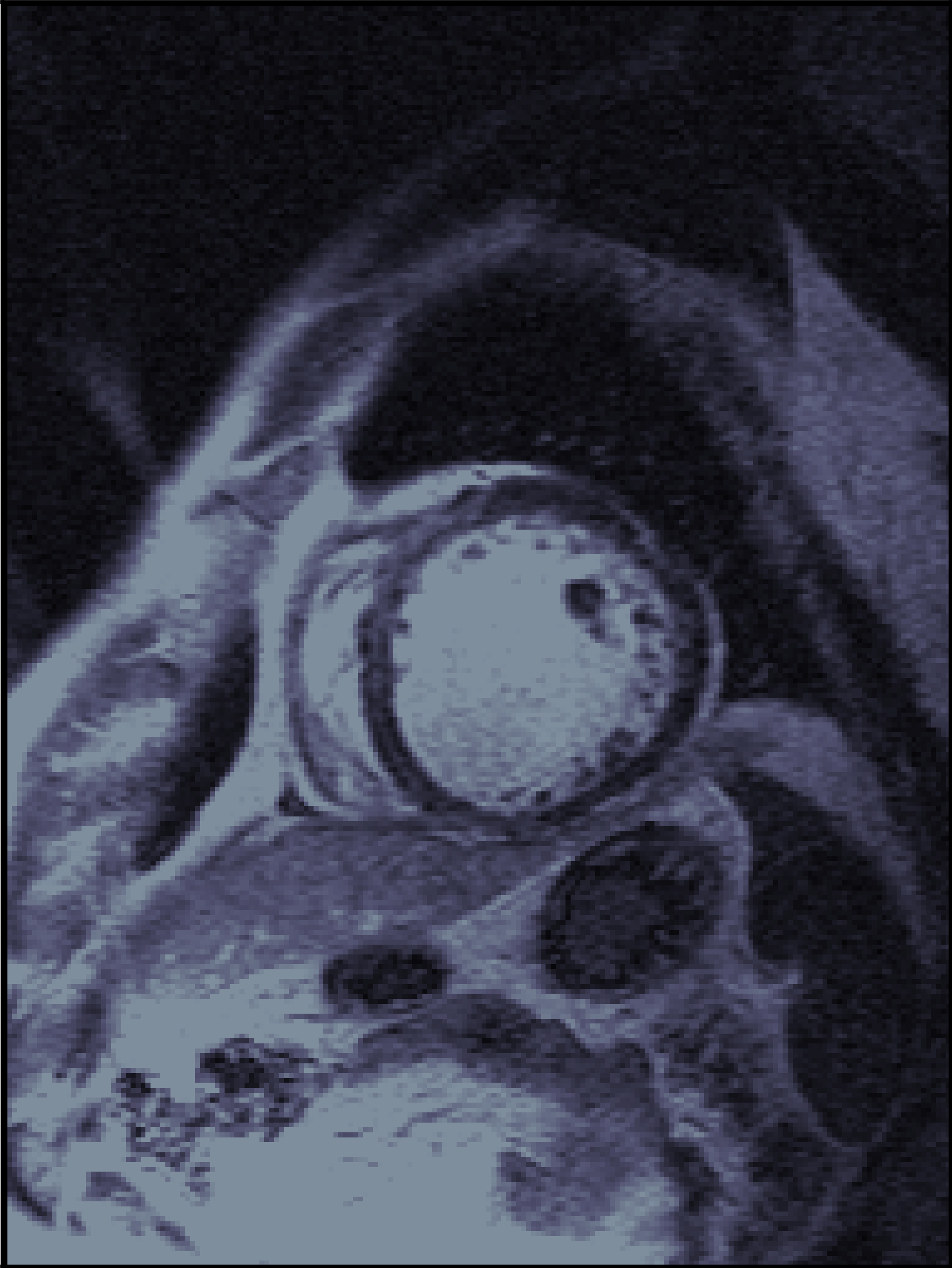

Supplement: S1 Dataset — (ZIP) [file pcbi.1007421.s001.zip › supplementary_segmented_lgemri_data/raw_data/07_18651/-114_ROW_20070822084753.png]

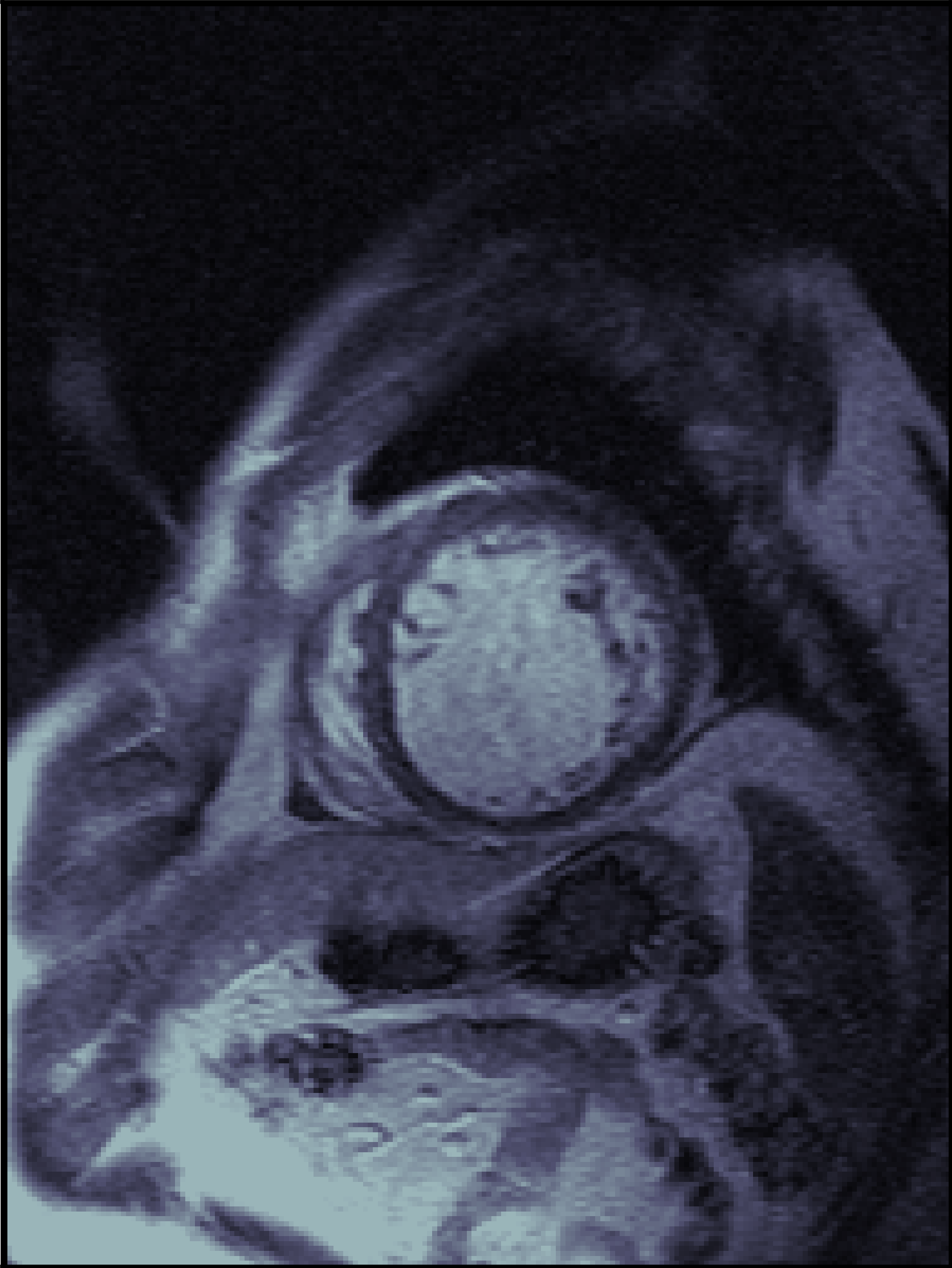

Supplement: S1 Dataset — (ZIP) [file pcbi.1007421.s001.zip › supplementary_segmented_lgemri_data/raw_data/07_18651/-124_ROW_20070822084820.png]

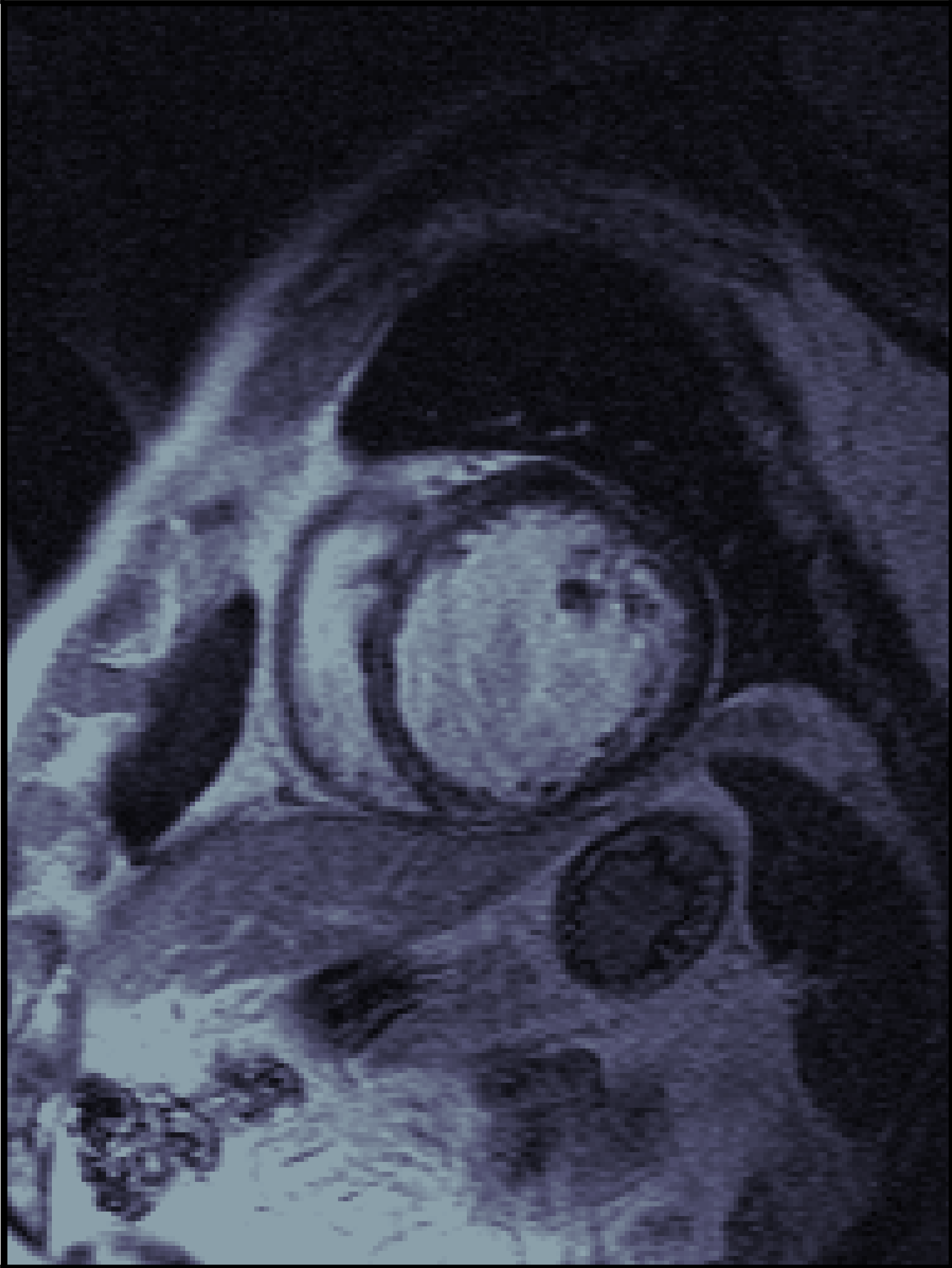

Supplement: S1 Dataset — (ZIP) [file pcbi.1007421.s001.zip › supplementary_segmented_lgemri_data/raw_data/07_18651/-104_ROW_20070822084727.png]

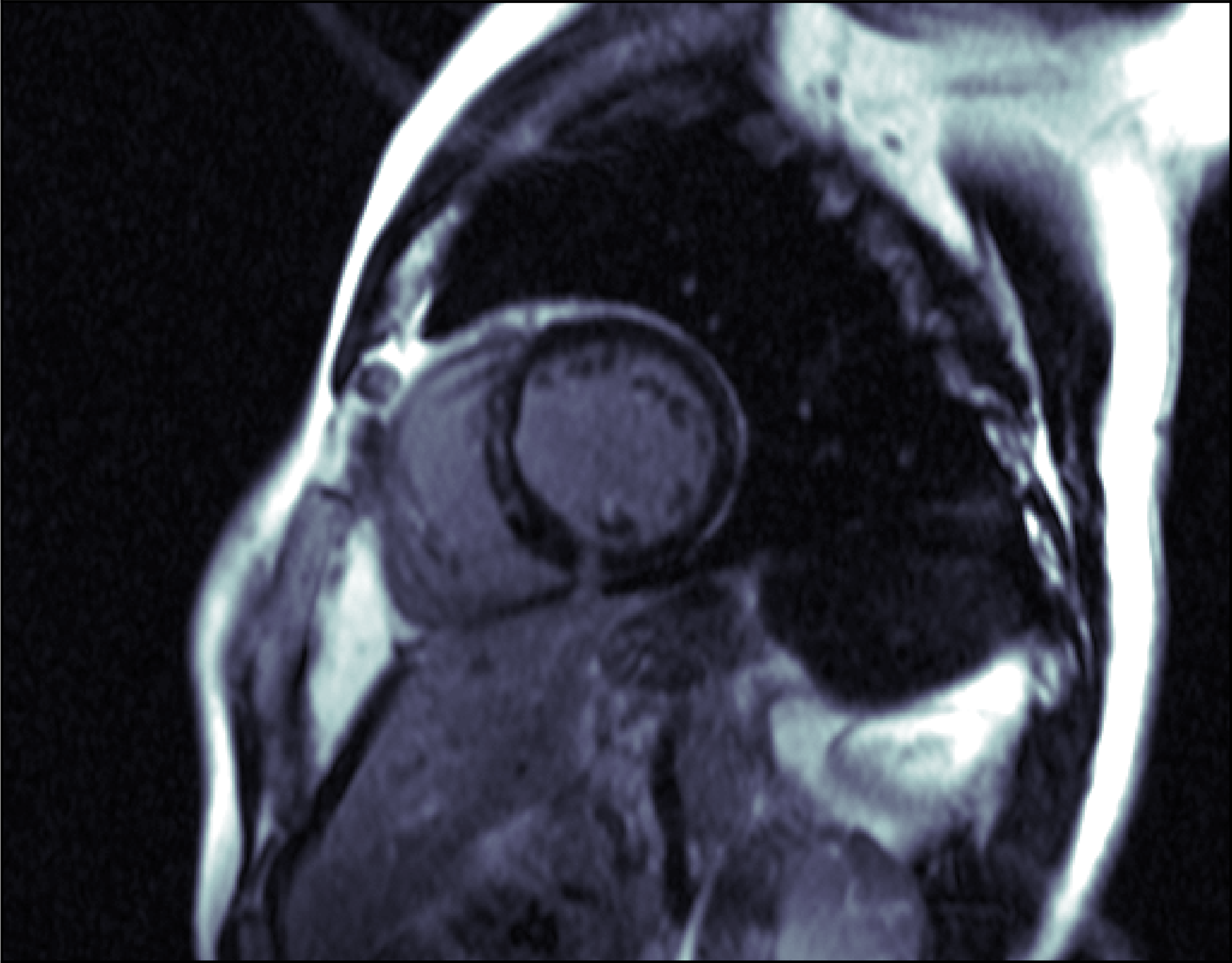

Supplement: S1 Dataset — (ZIP) [file pcbi.1007421.s001.zip › supplementary_segmented_lgemri_data/raw_data/07_18643/70_COL_20071005130111.png]

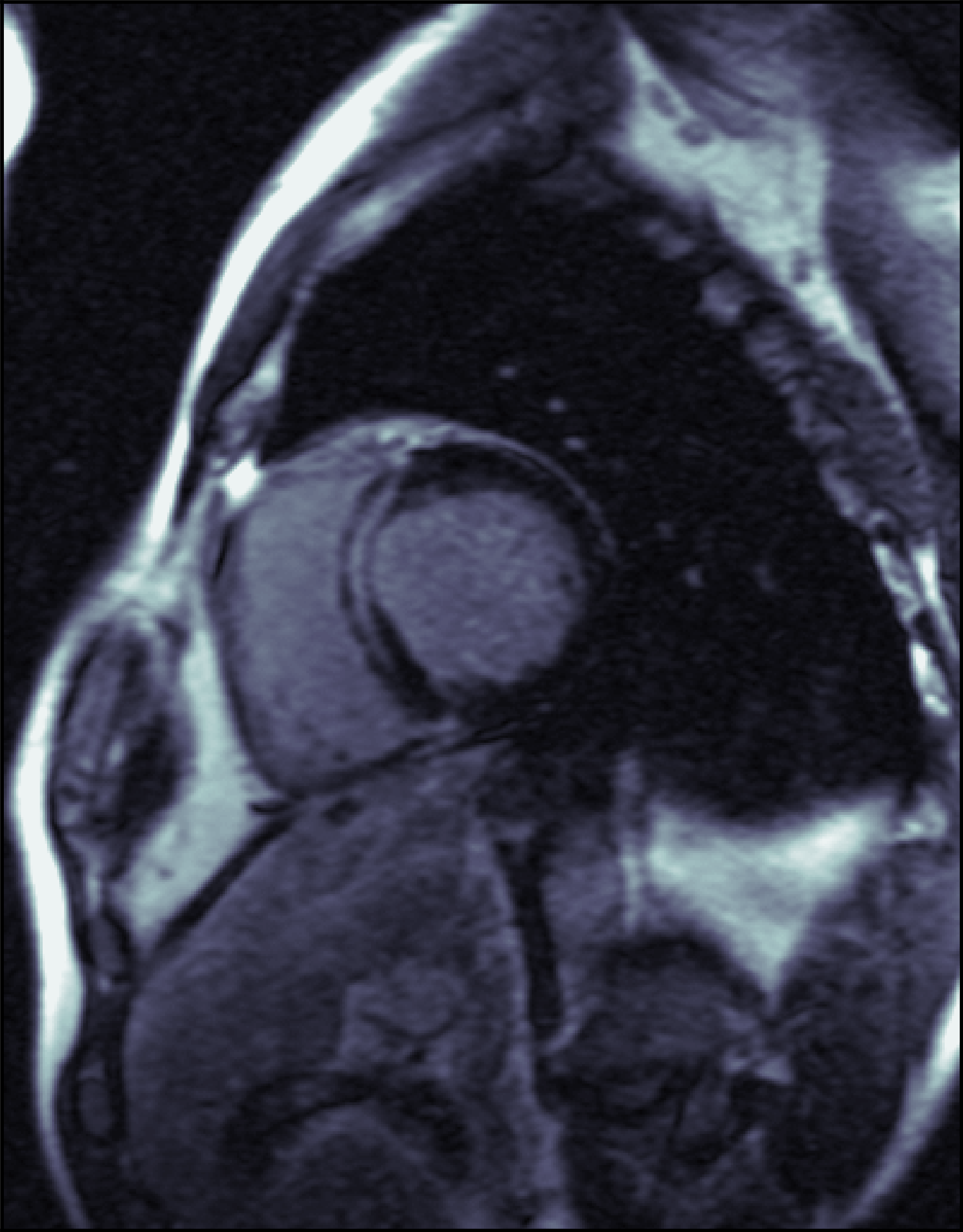

Supplement: S1 Dataset — (ZIP) [file pcbi.1007421.s001.zip › supplementary_segmented_lgemri_data/raw_data/07_18643/59_ROW_20071005125548.png]

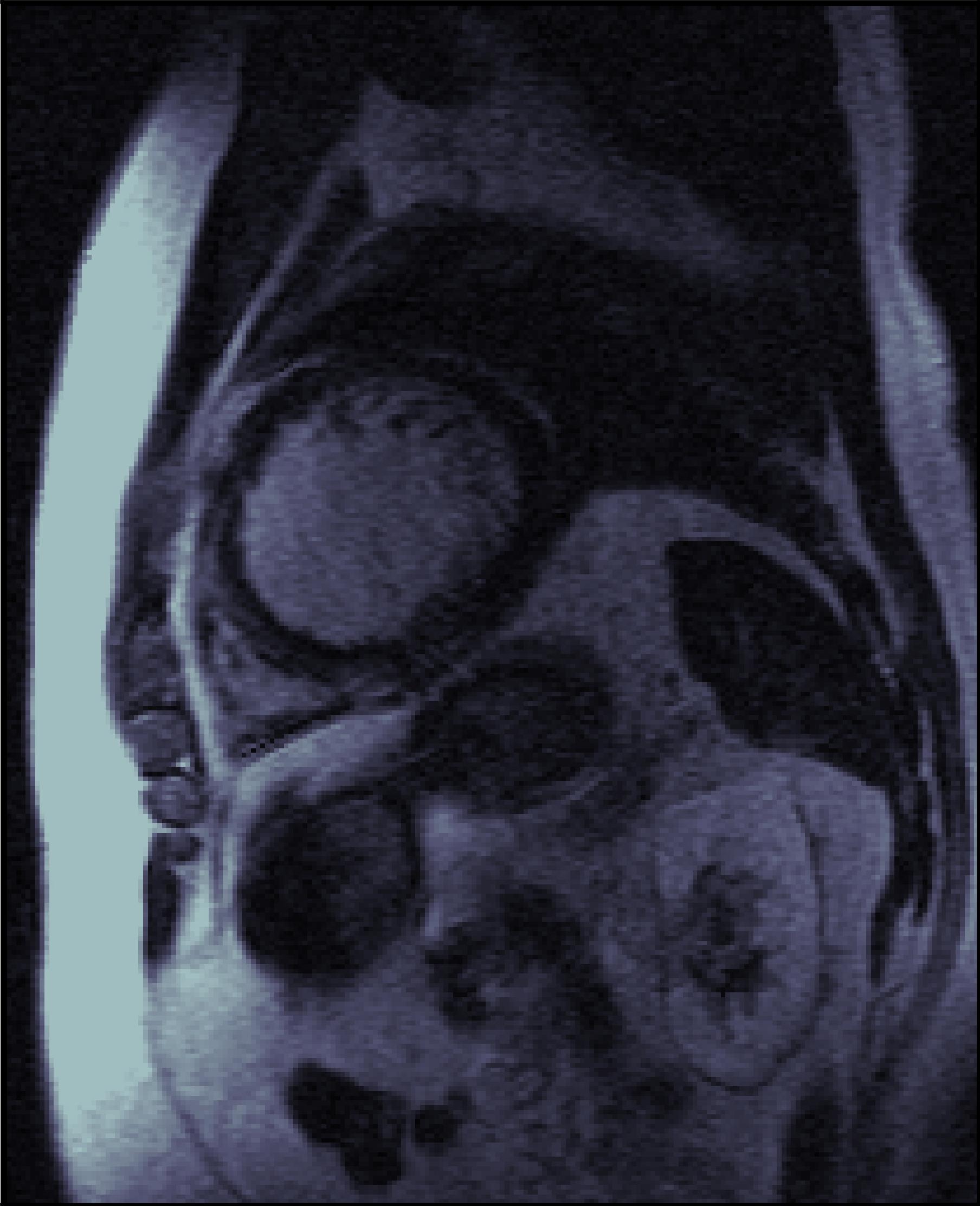

Supplement: S1 Dataset — (ZIP) [file pcbi.1007421.s001.zip › supplementary_segmented_lgemri_data/raw_data/07_14899/95_ROW_20070627154921.png]

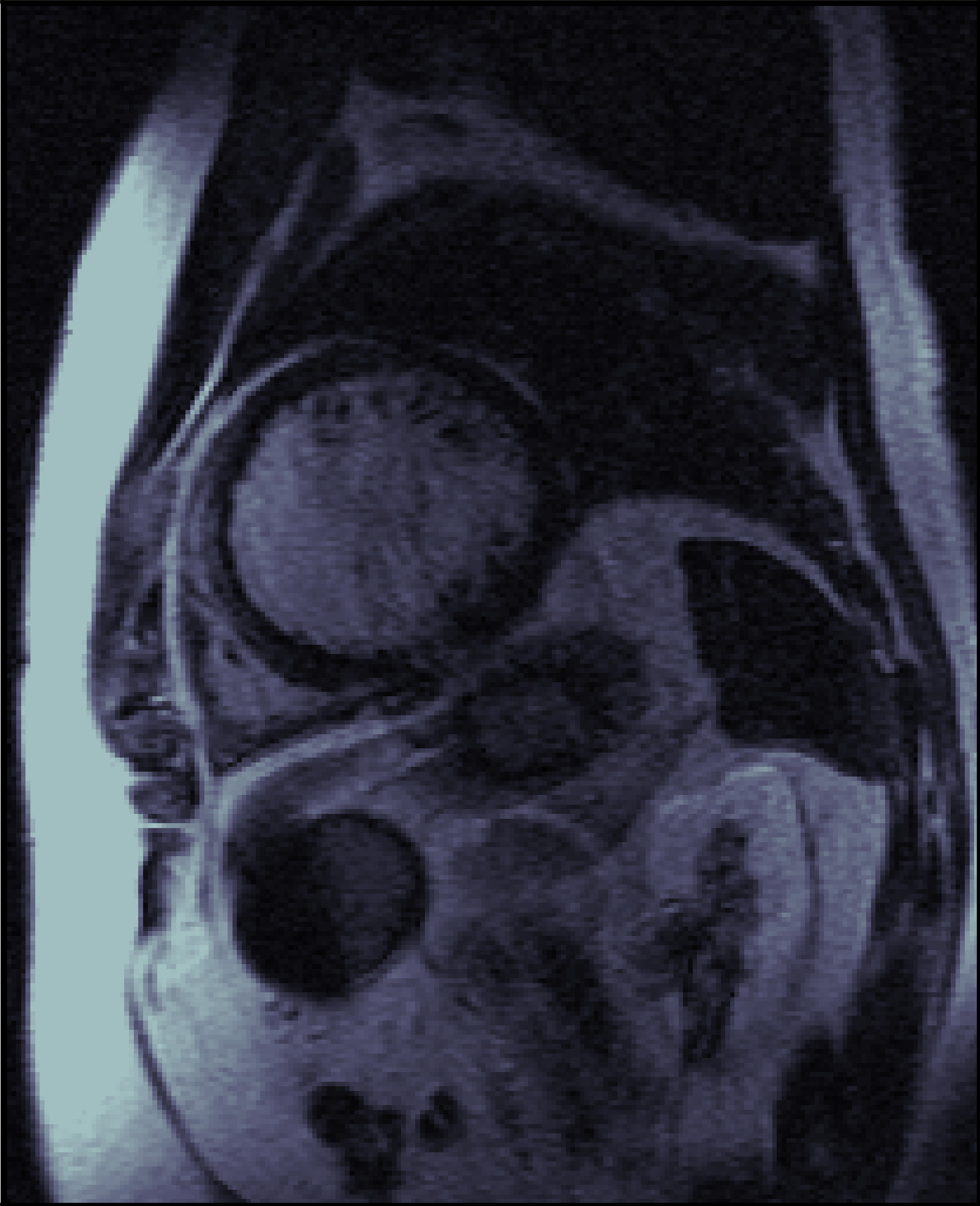

Supplement: S1 Dataset — (ZIP) [file pcbi.1007421.s001.zip › supplementary_segmented_lgemri_data/raw_data/07_14899/85_ROW_20070627154846.png]

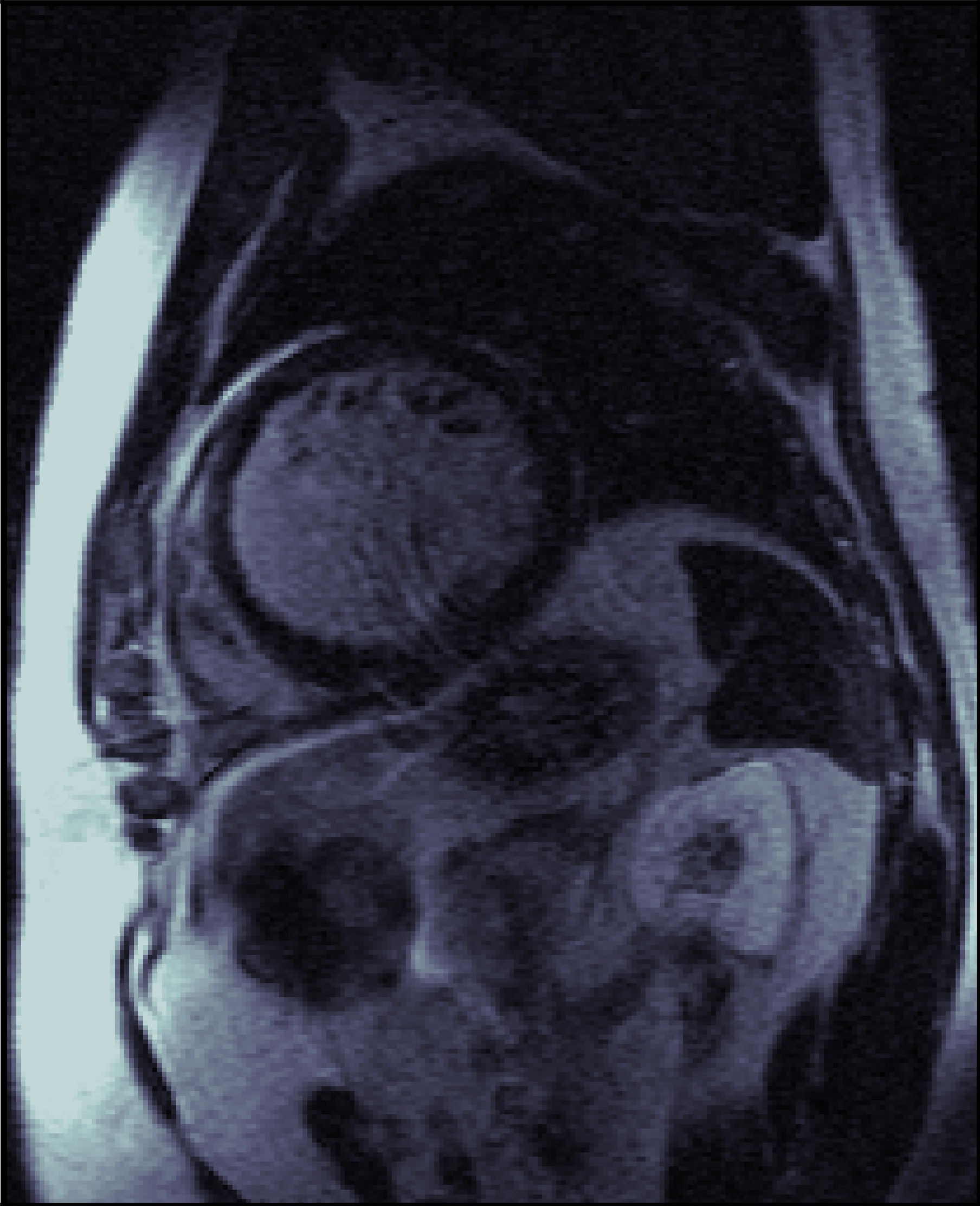

Supplement: S1 Dataset — (ZIP) [file pcbi.1007421.s001.zip › supplementary_segmented_lgemri_data/raw_data/07_14899/75_ROW_20070627154815.png]

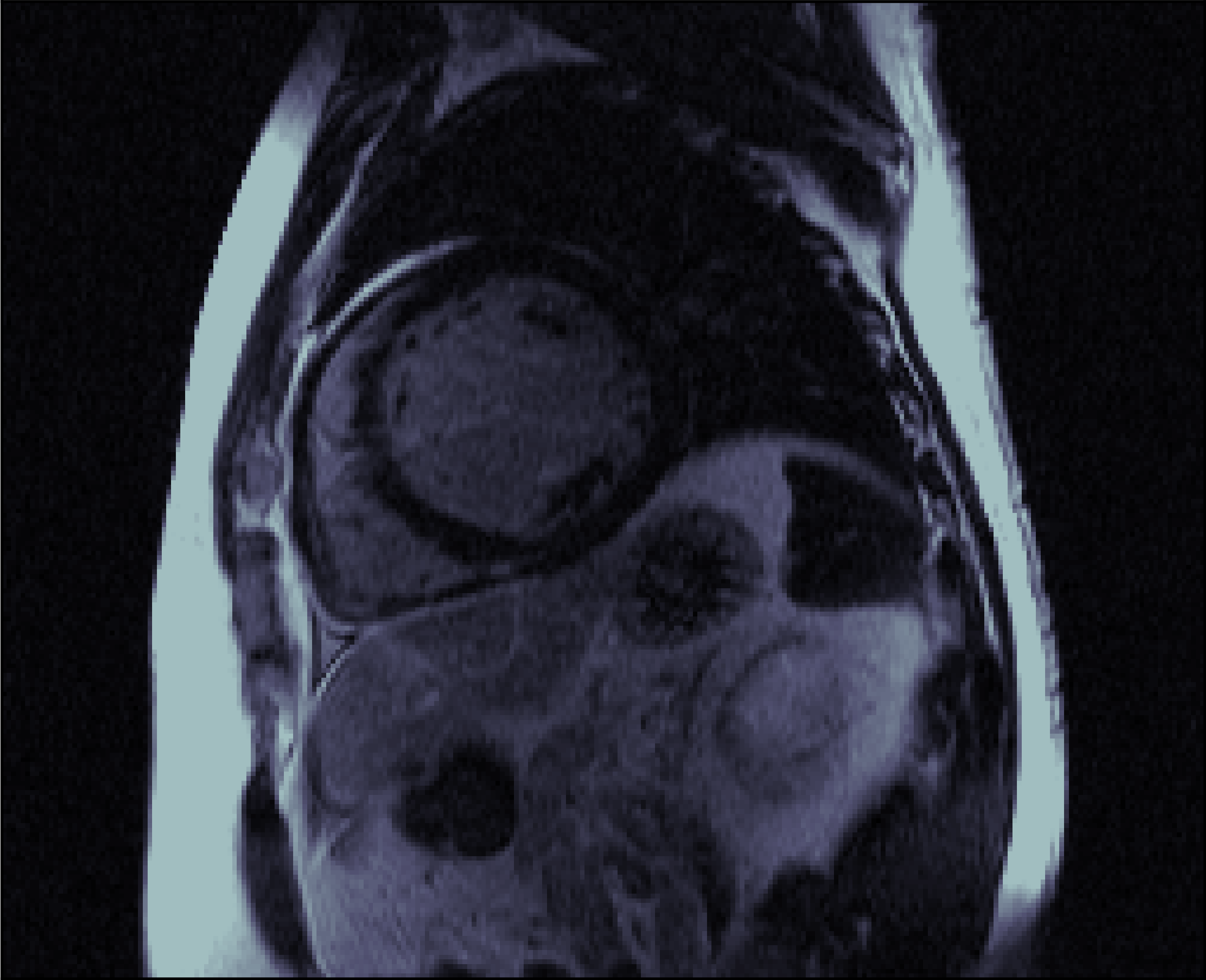

Supplement: S1 Dataset — (ZIP) [file pcbi.1007421.s001.zip › supplementary_segmented_lgemri_data/raw_data/07_14899/55_COL_20070627155347.png]

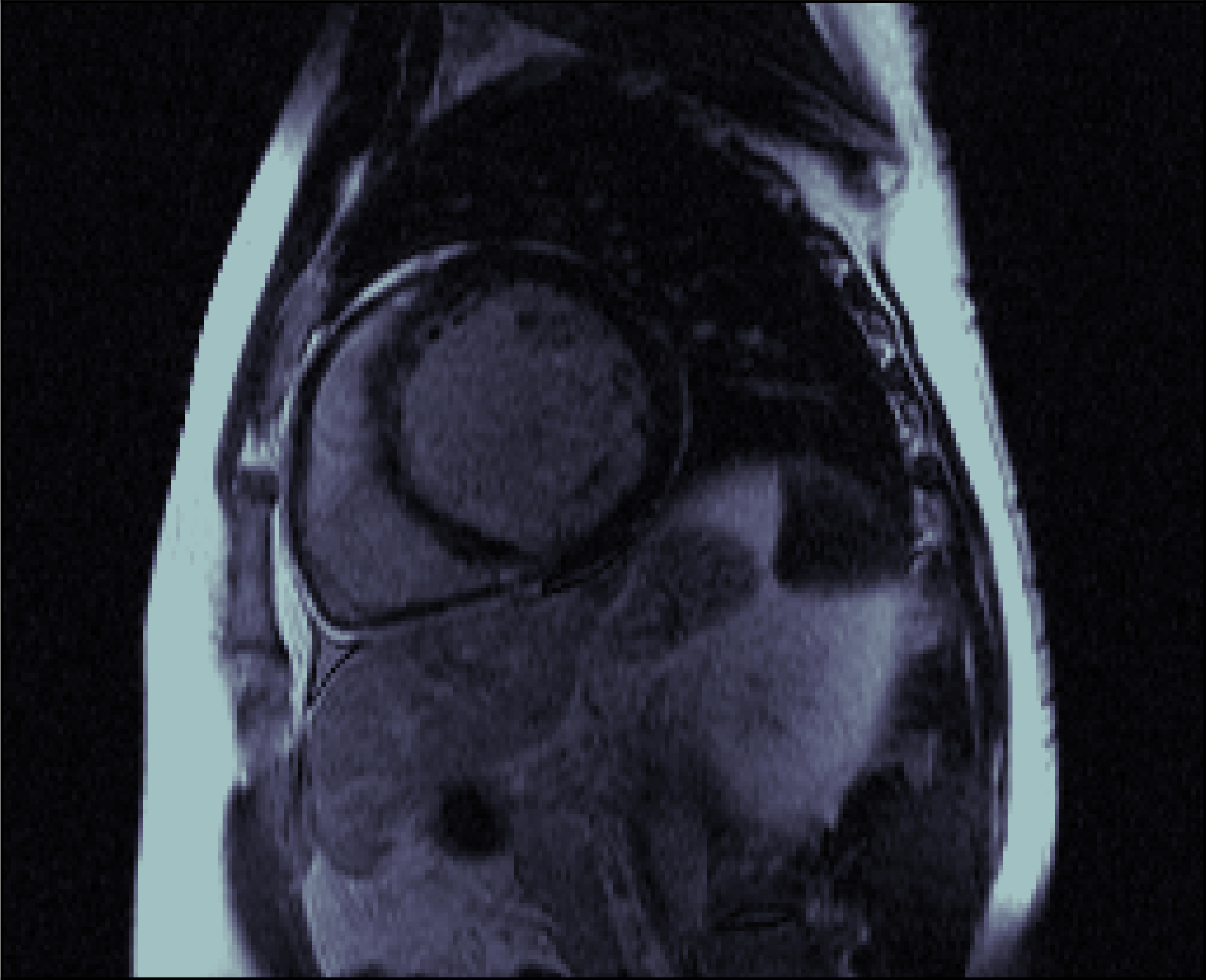

Supplement: S1 Dataset — (ZIP) [file pcbi.1007421.s001.zip › supplementary_segmented_lgemri_data/raw_data/07_14899/45_COL_20070627155251.png]

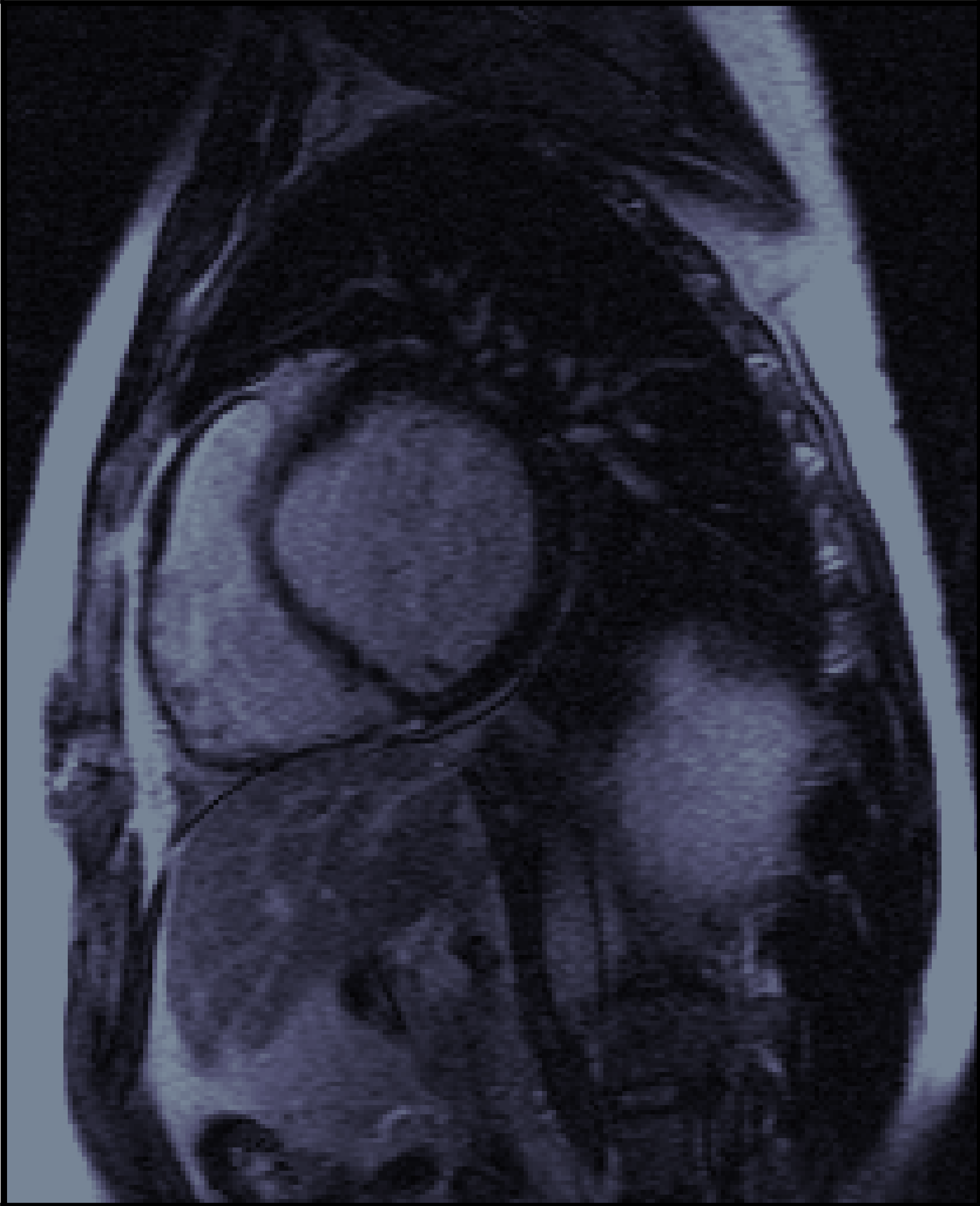

Supplement: S1 Dataset — (ZIP) [file pcbi.1007421.s001.zip › supplementary_segmented_lgemri_data/raw_data/07_14899/35_ROW_20070627154437.png]

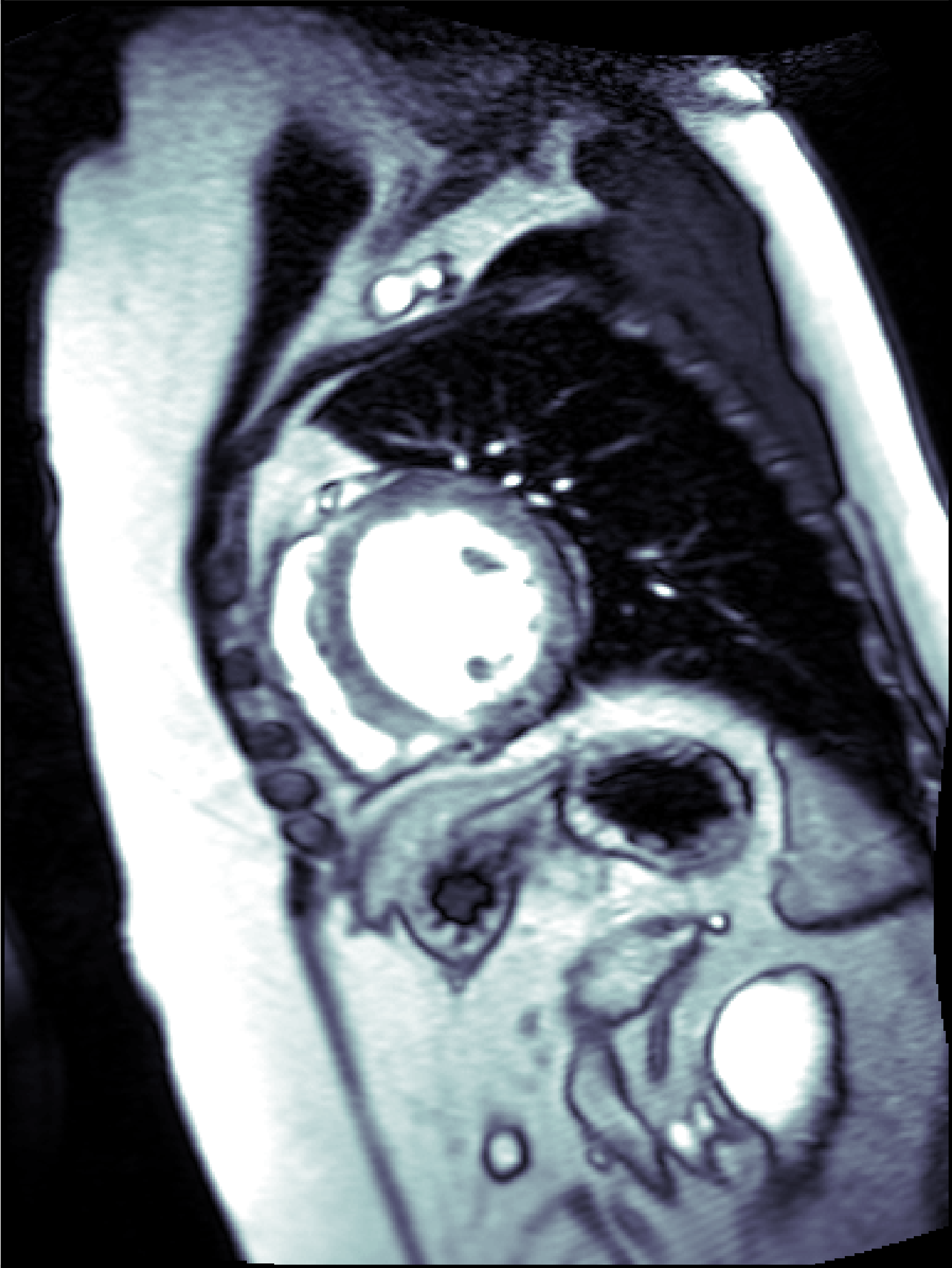

Supplement: S1 Dataset — (ZIP) [file pcbi.1007421.s001.zip › supplementary_segmented_lgemri_data/raw_data/07_14321/50_ROW_20121211122454.png]

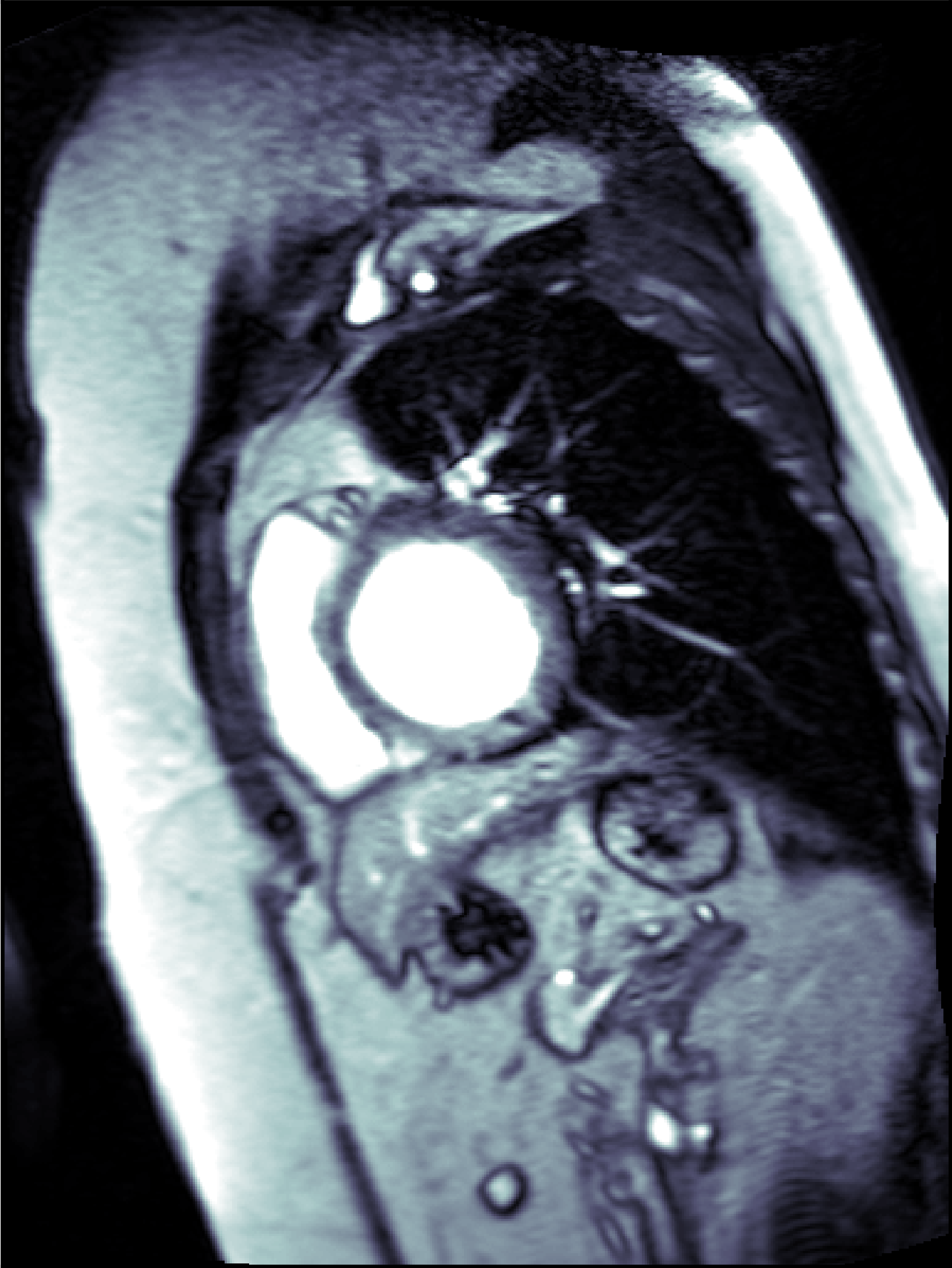

Supplement: S1 Dataset — (ZIP) [file pcbi.1007421.s001.zip › supplementary_segmented_lgemri_data/raw_data/07_14321/30_ROW_20121211122344.png]

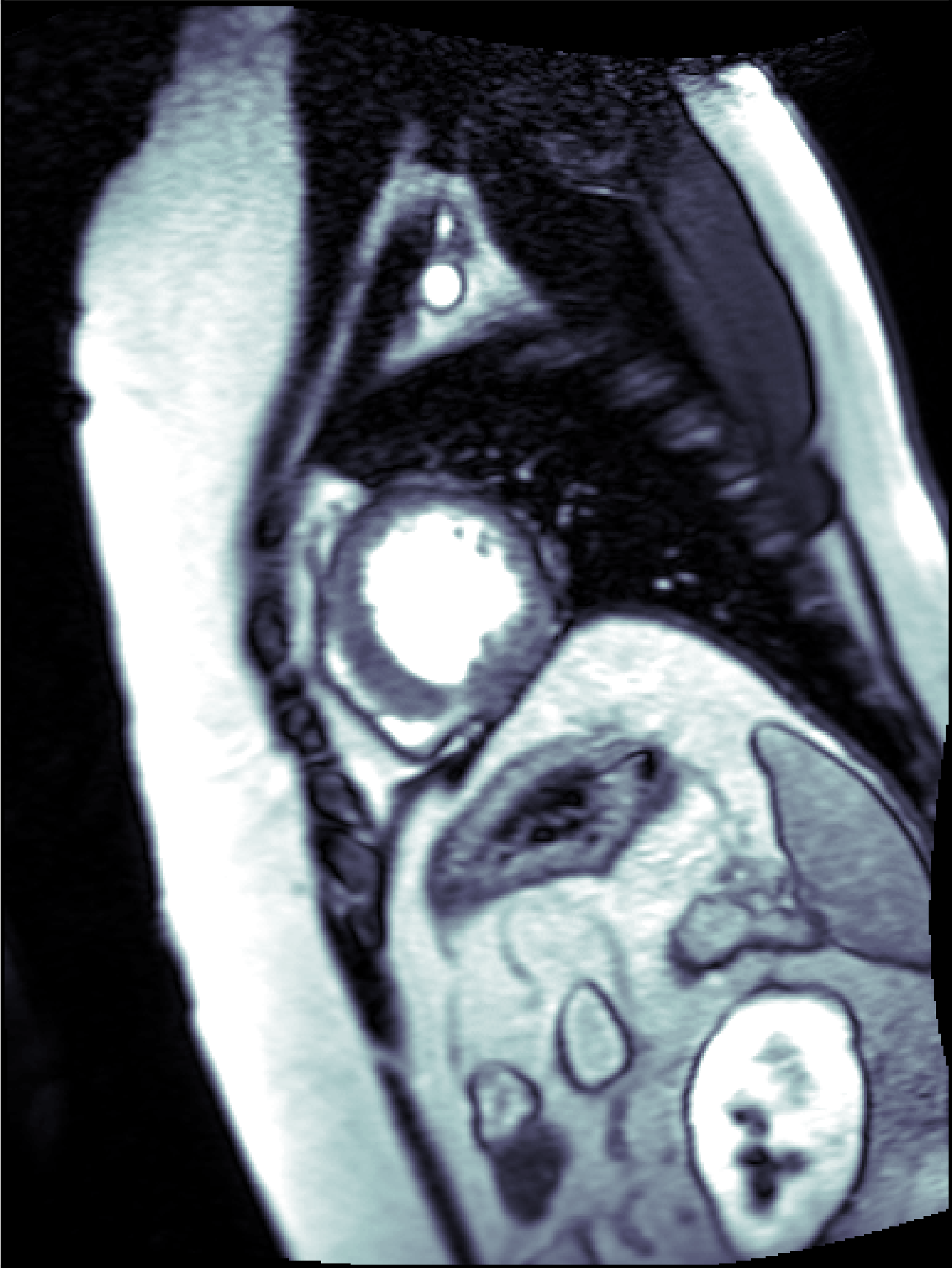

Supplement: S1 Dataset — (ZIP) [file pcbi.1007421.s001.zip › supplementary_segmented_lgemri_data/raw_data/07_14321/80_ROW_20121211122552.png]

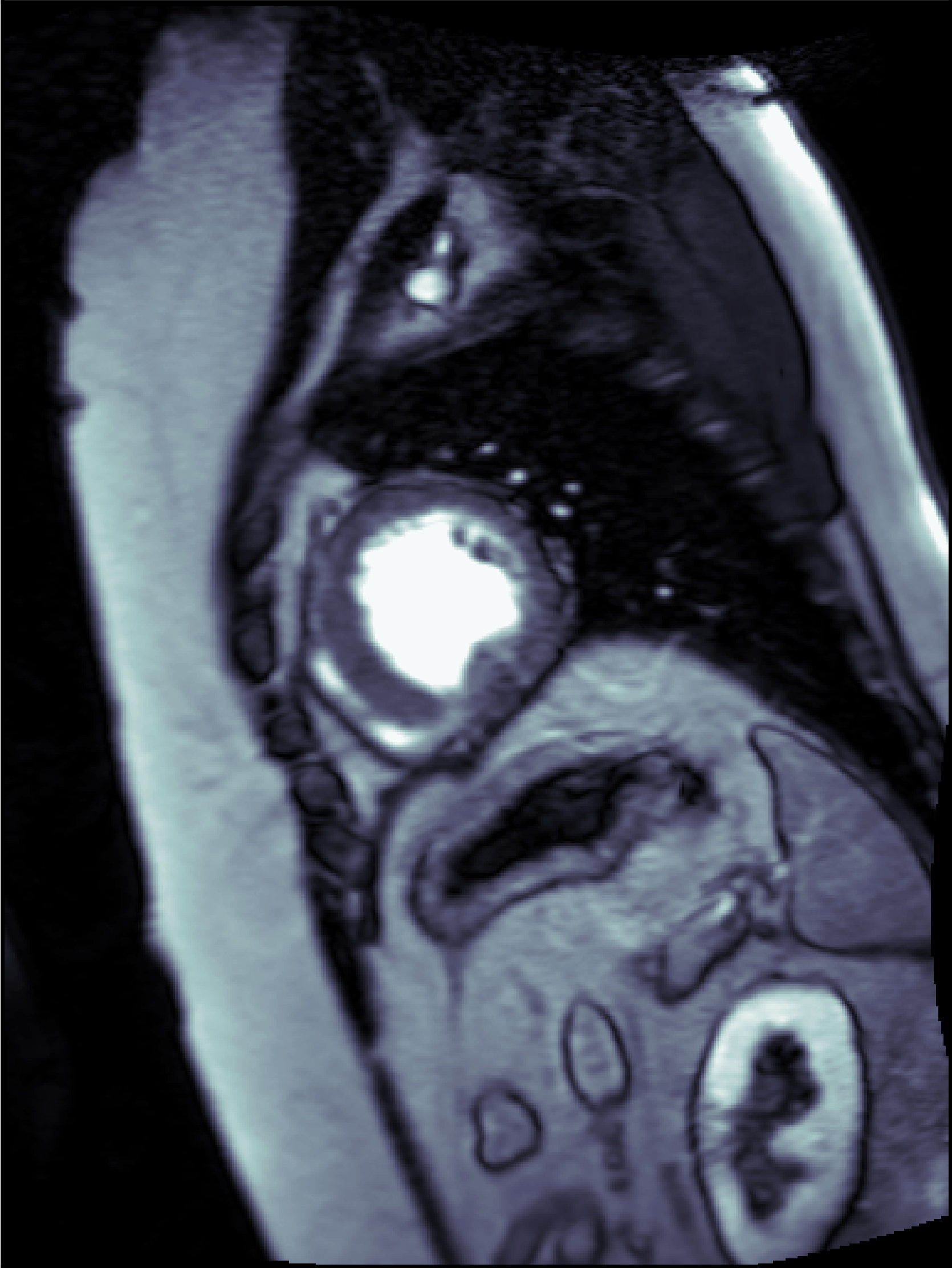

Supplement: S1 Dataset — (ZIP) [file pcbi.1007421.s001.zip › supplementary_segmented_lgemri_data/raw_data/07_14321/70_ROW_20121211122531.png]

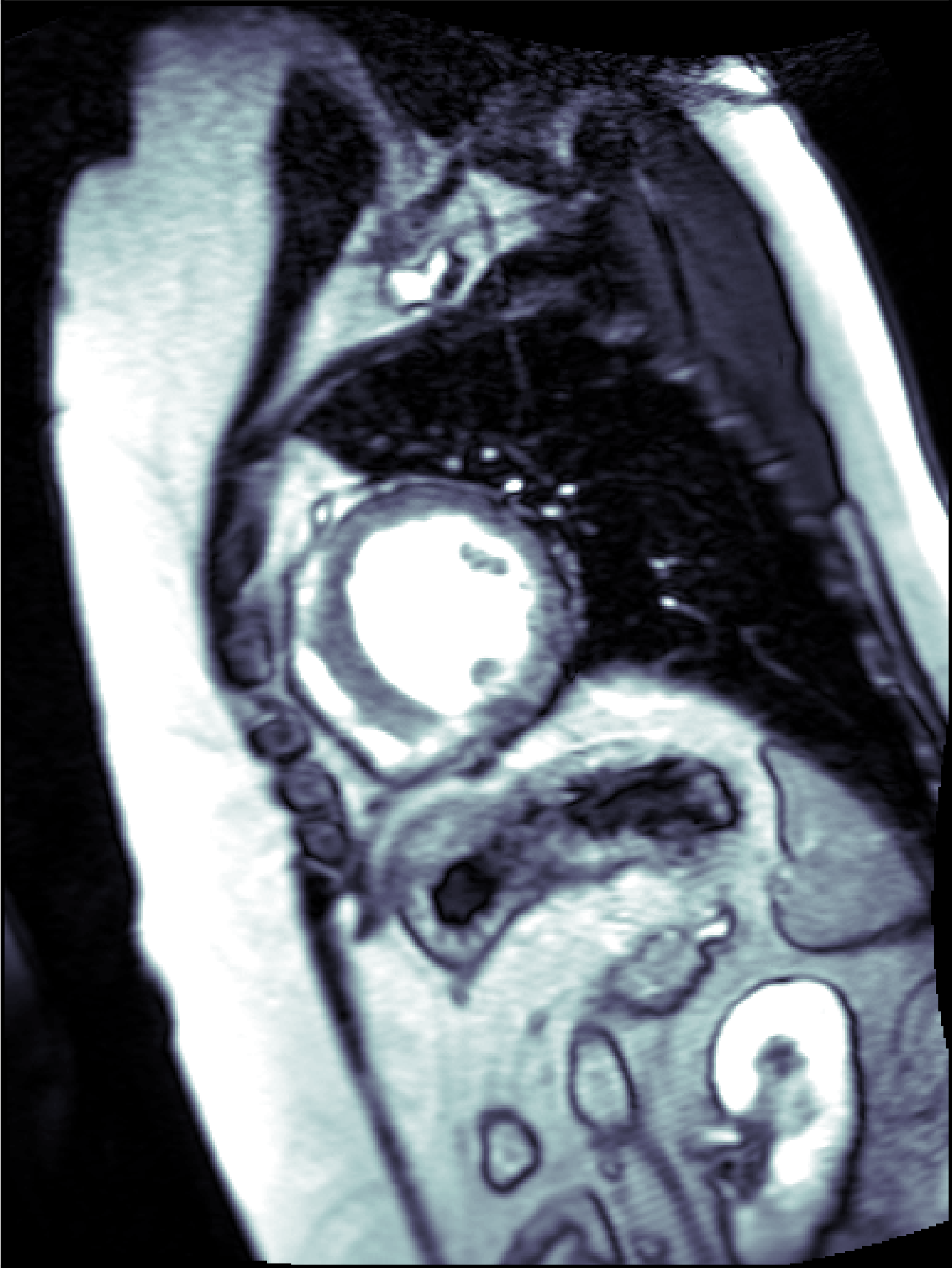

Supplement: S1 Dataset — (ZIP) [file pcbi.1007421.s001.zip › supplementary_segmented_lgemri_data/raw_data/07_14321/60_ROW_20121211122512.png]

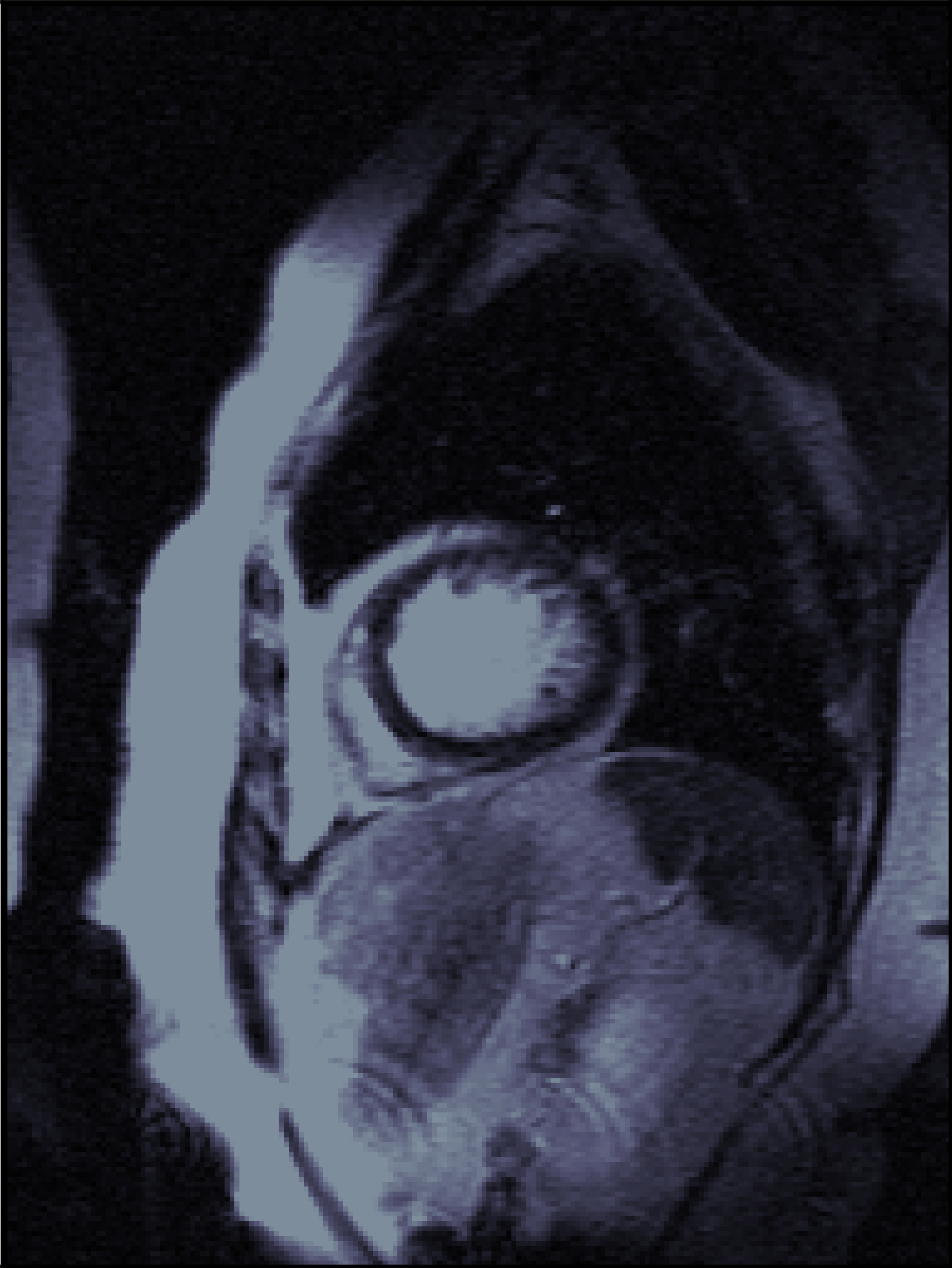

Supplement: S1 Dataset — (ZIP) [file pcbi.1007421.s001.zip › supplementary_segmented_lgemri_data/raw_data/07_10248/109_ROW_20070605125451.png]

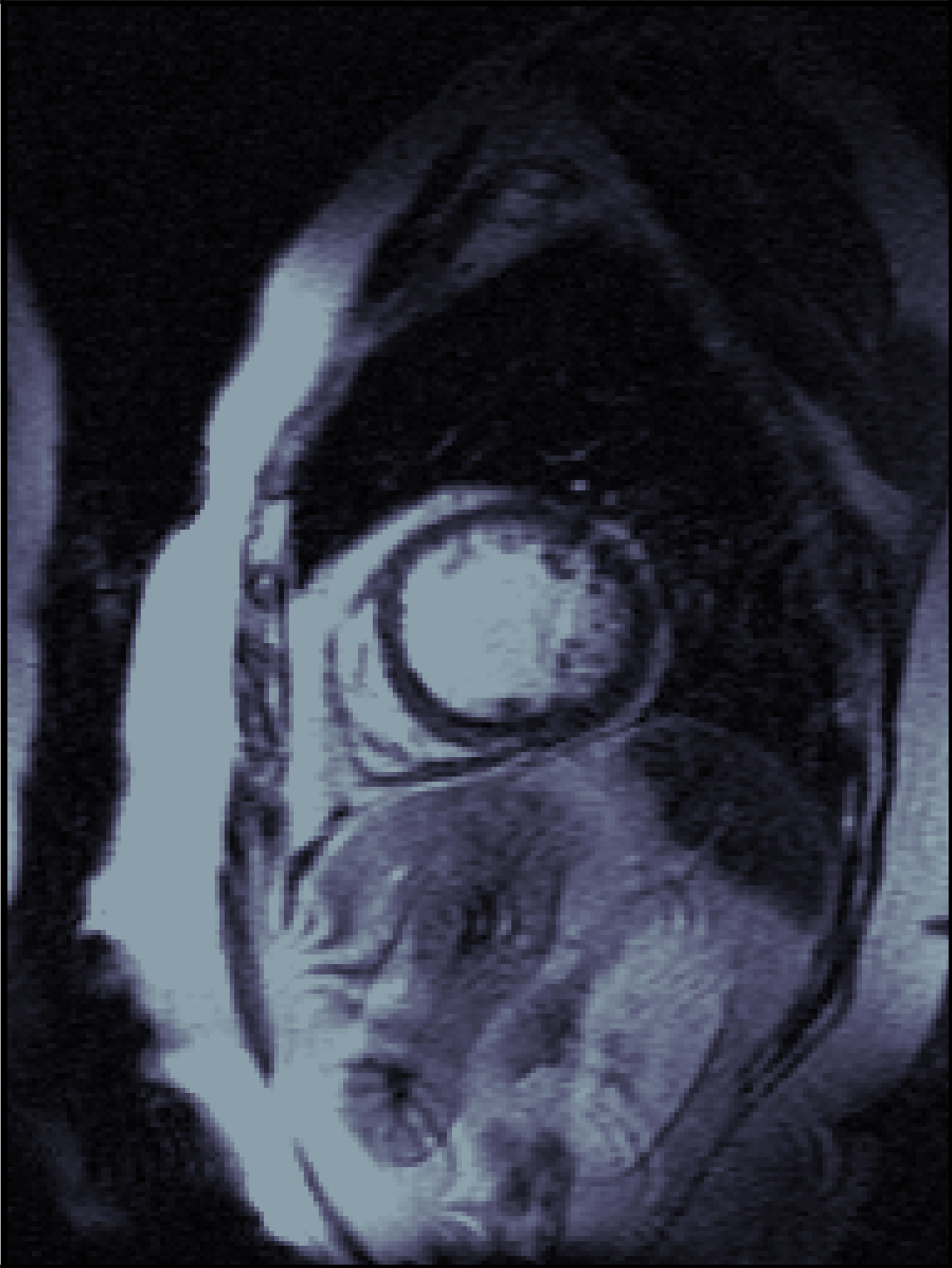

Supplement: S1 Dataset — (ZIP) [file pcbi.1007421.s001.zip › supplementary_segmented_lgemri_data/raw_data/07_10248/100_ROW_20070605125426.png]

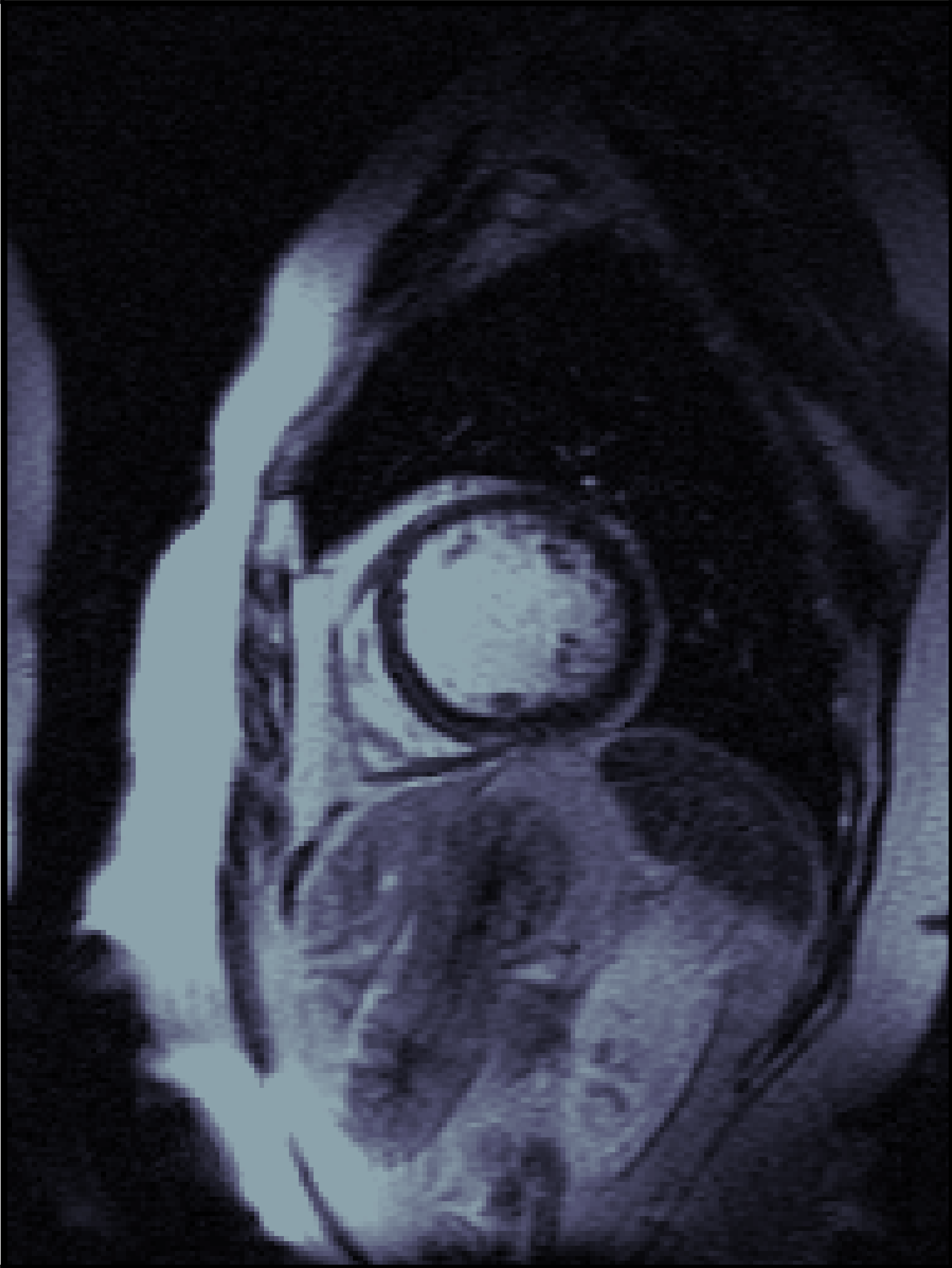

Supplement: S1 Dataset — (ZIP) [file pcbi.1007421.s001.zip › supplementary_segmented_lgemri_data/raw_data/07_10248/100_ROW_20070605125359.png]

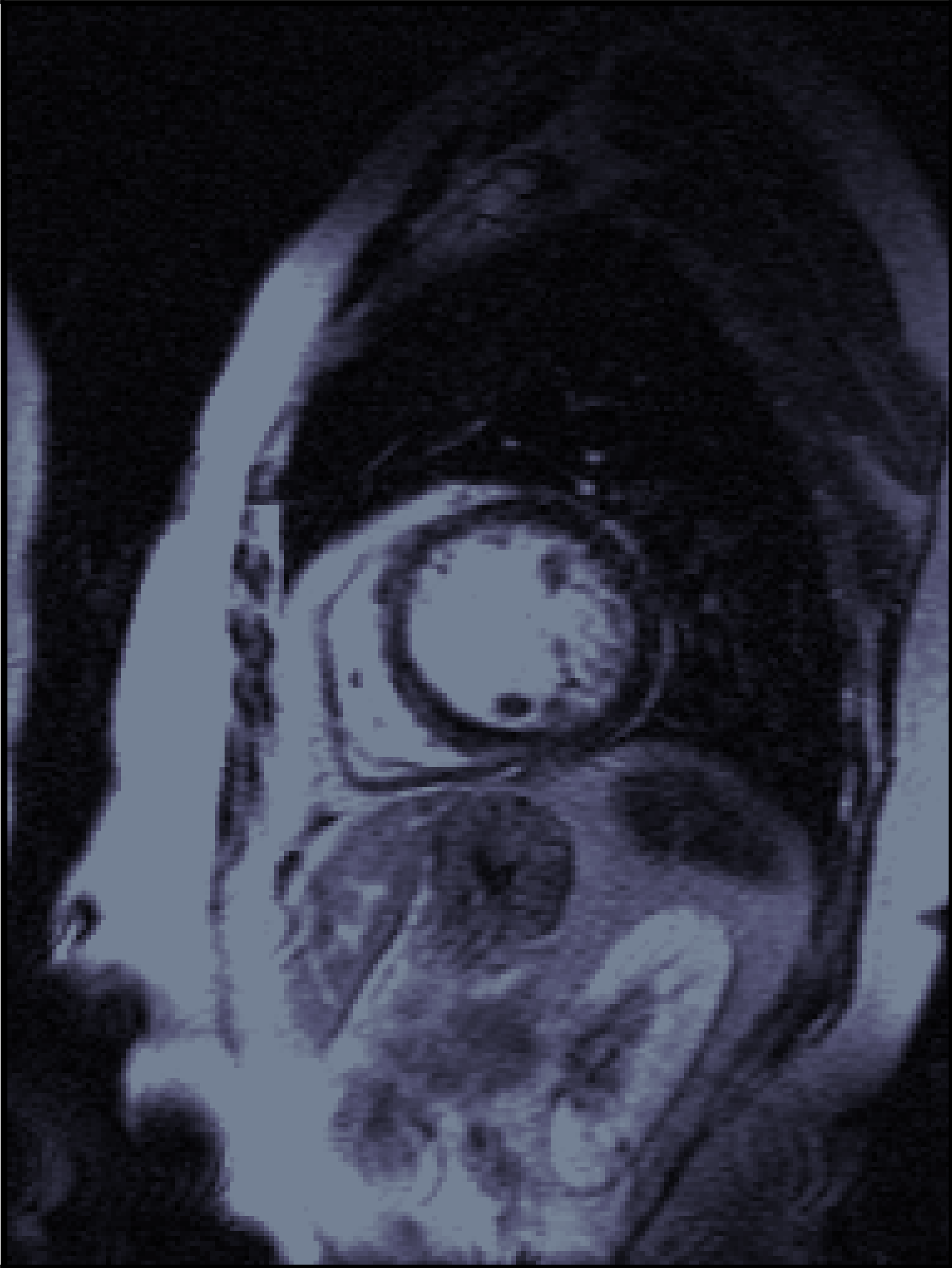

Supplement: S1 Dataset — (ZIP) [file pcbi.1007421.s001.zip › supplementary_segmented_lgemri_data/raw_data/07_10248/90_ROW_20070605125335.png]

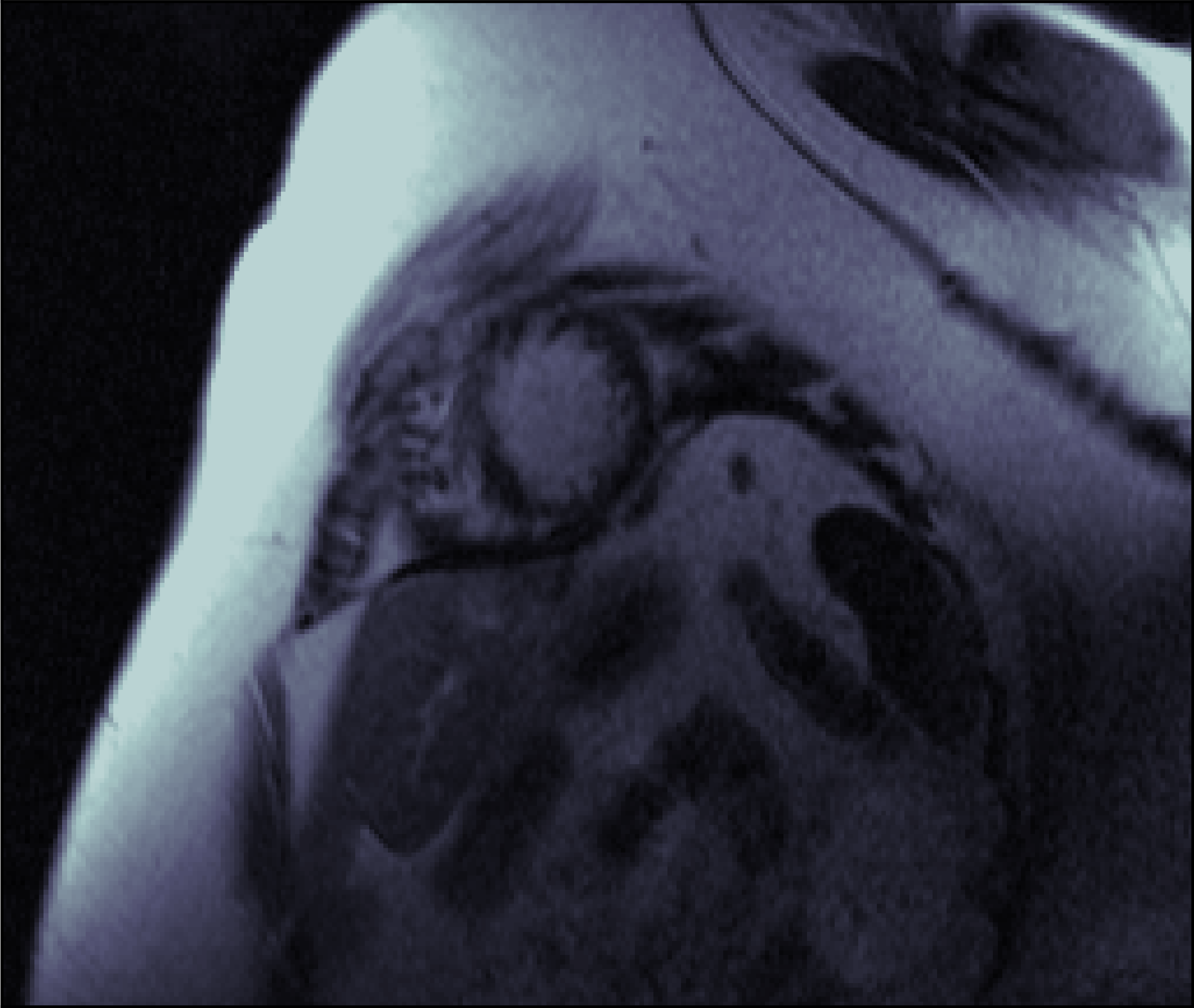

Supplement: S1 Dataset — (ZIP) [file pcbi.1007421.s001.zip › supplementary_segmented_lgemri_data/raw_data/07_04952/126_COL_20070515111212.png]

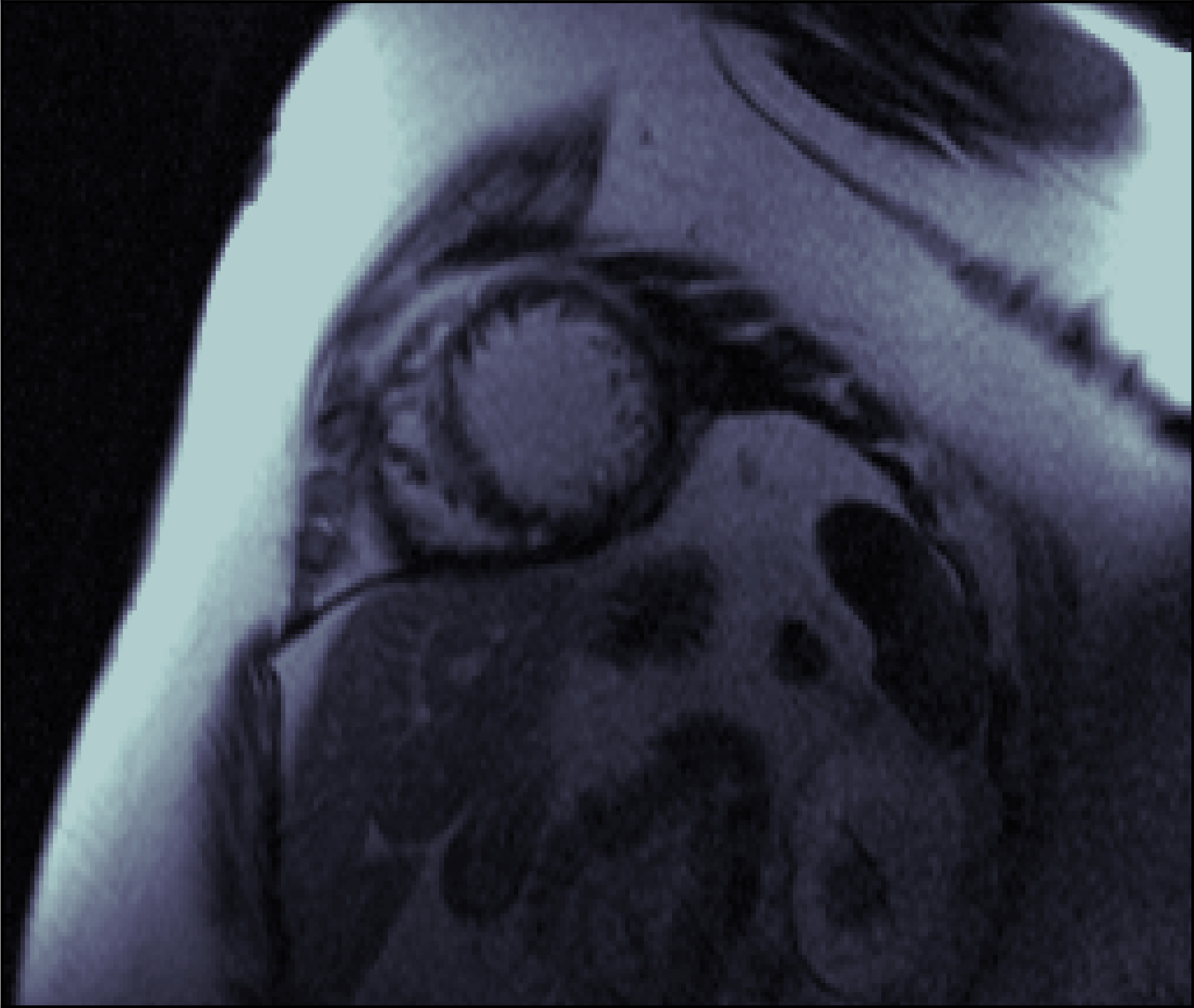

Supplement: S1 Dataset — (ZIP) [file pcbi.1007421.s001.zip › supplementary_segmented_lgemri_data/raw_data/07_04952/116_COL_20070515111120.png]

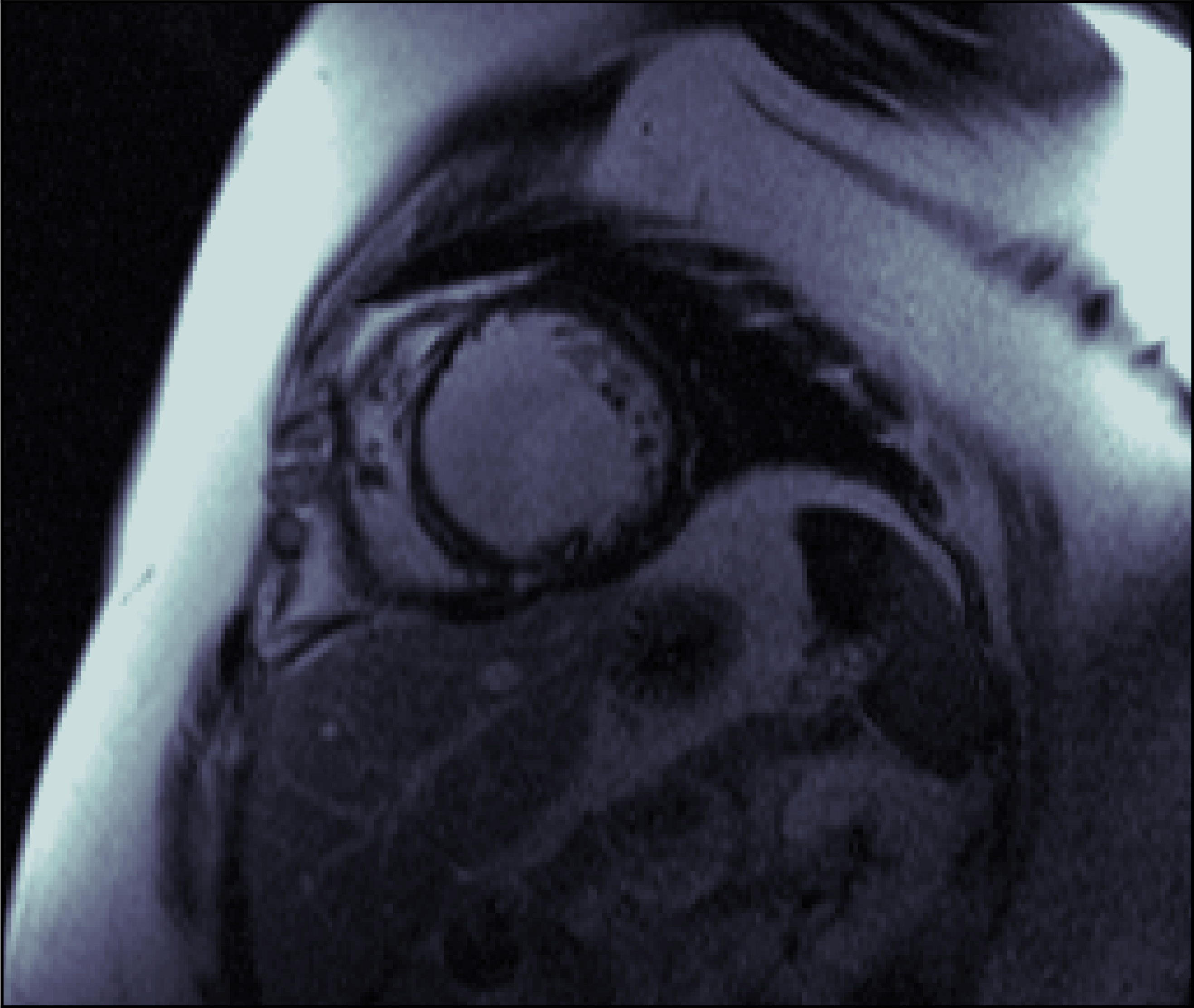

Supplement: S1 Dataset — (ZIP) [file pcbi.1007421.s001.zip › supplementary_segmented_lgemri_data/raw_data/07_04952/106_COL_20070515111044.png]

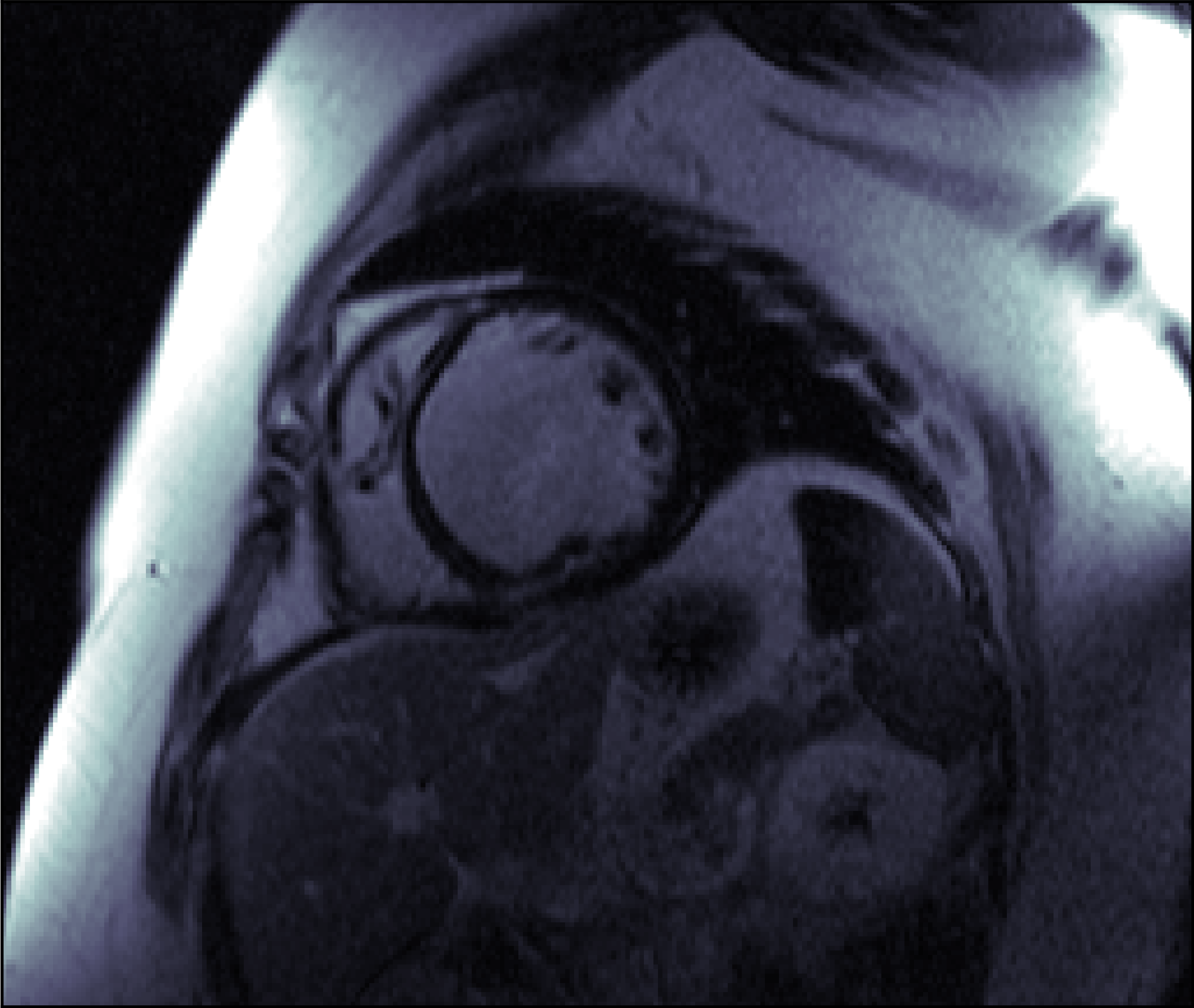

Supplement: S1 Dataset — (ZIP) [file pcbi.1007421.s001.zip › supplementary_segmented_lgemri_data/raw_data/07_04952/96_COL_20070515110959.png]

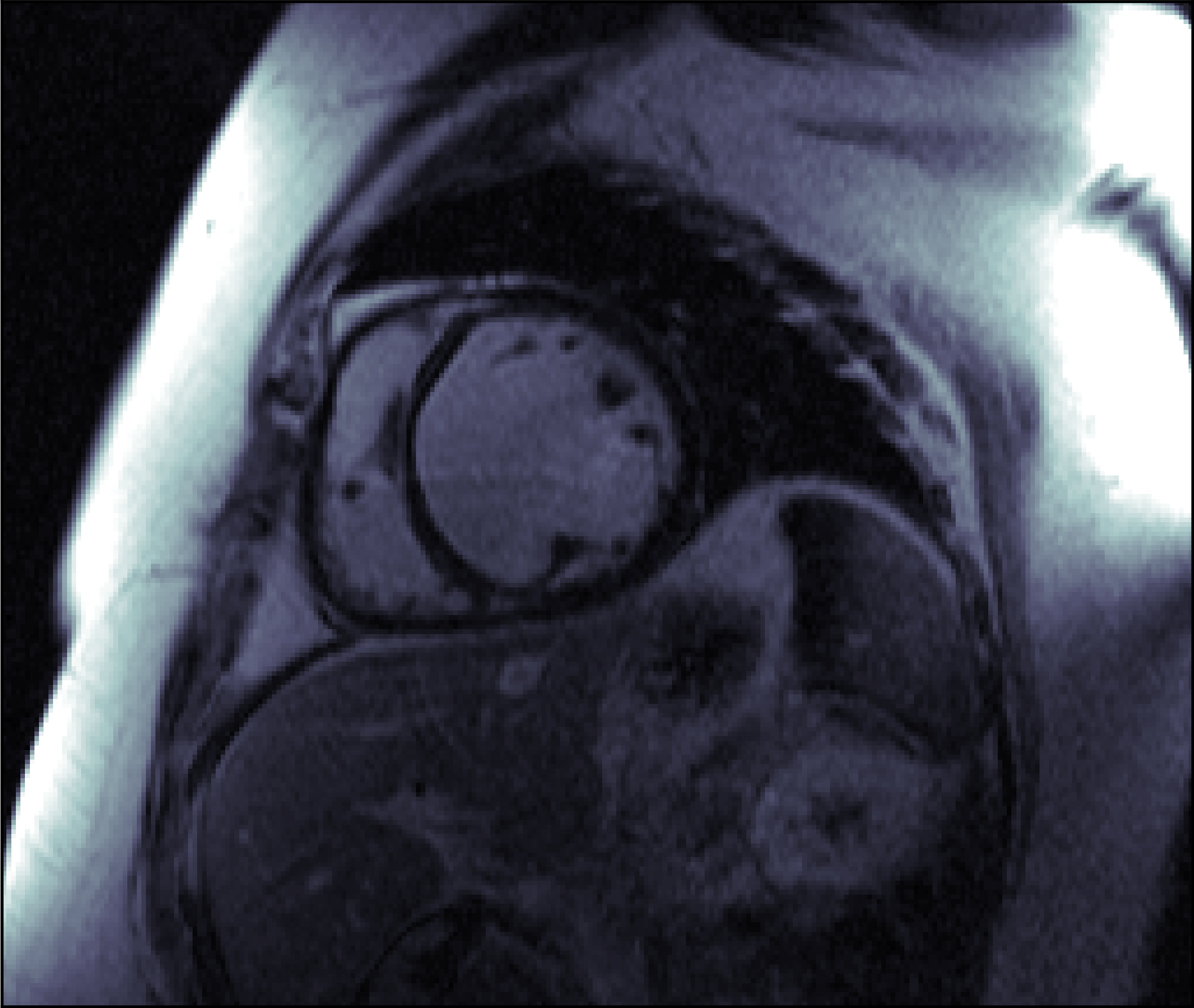

Supplement: S1 Dataset — (ZIP) [file pcbi.1007421.s001.zip › supplementary_segmented_lgemri_data/raw_data/07_04952/87_COL_20070515110910.png]

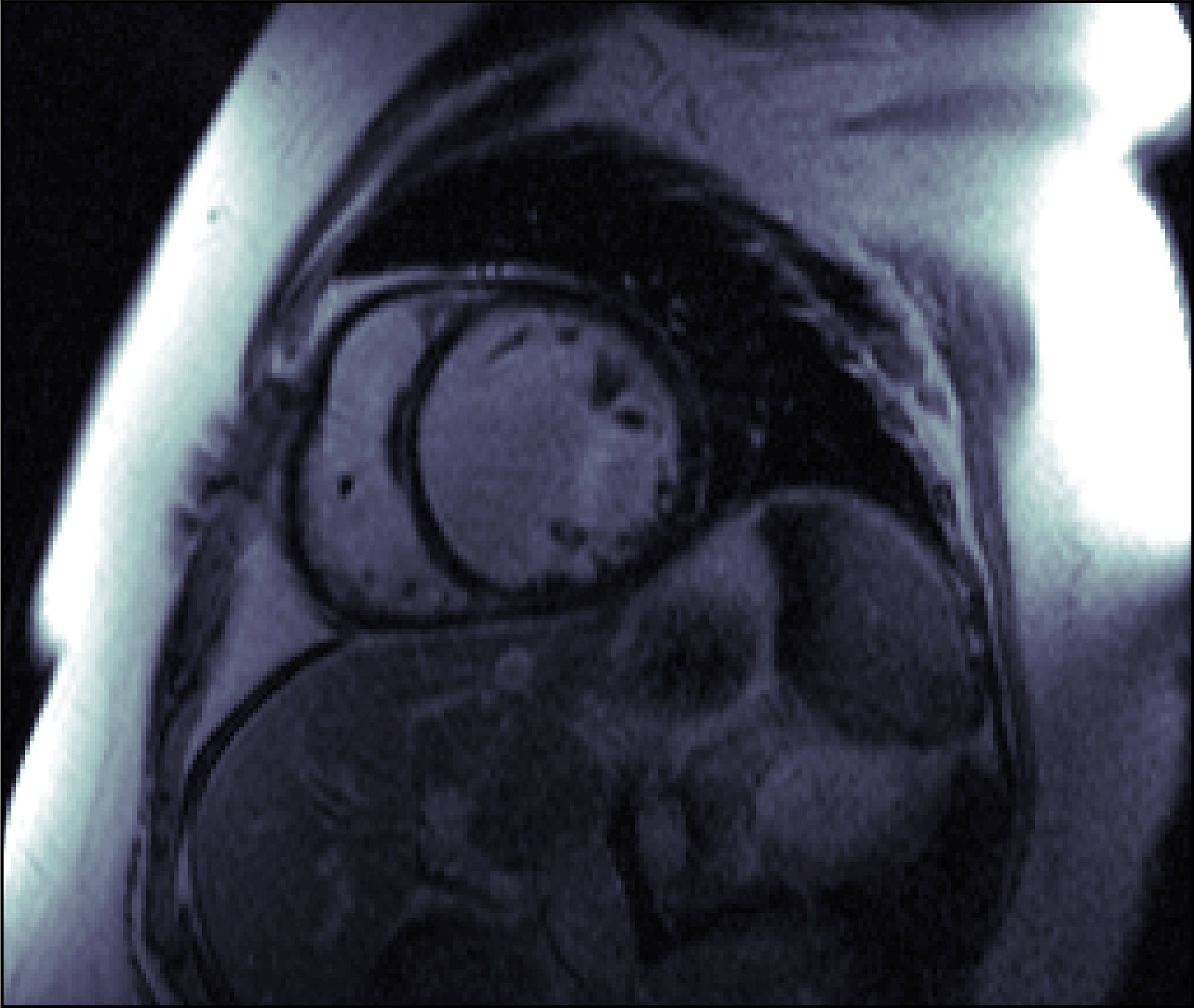

Supplement: S1 Dataset — (ZIP) [file pcbi.1007421.s001.zip › supplementary_segmented_lgemri_data/raw_data/07_04952/76_COL_20070515110821.png]

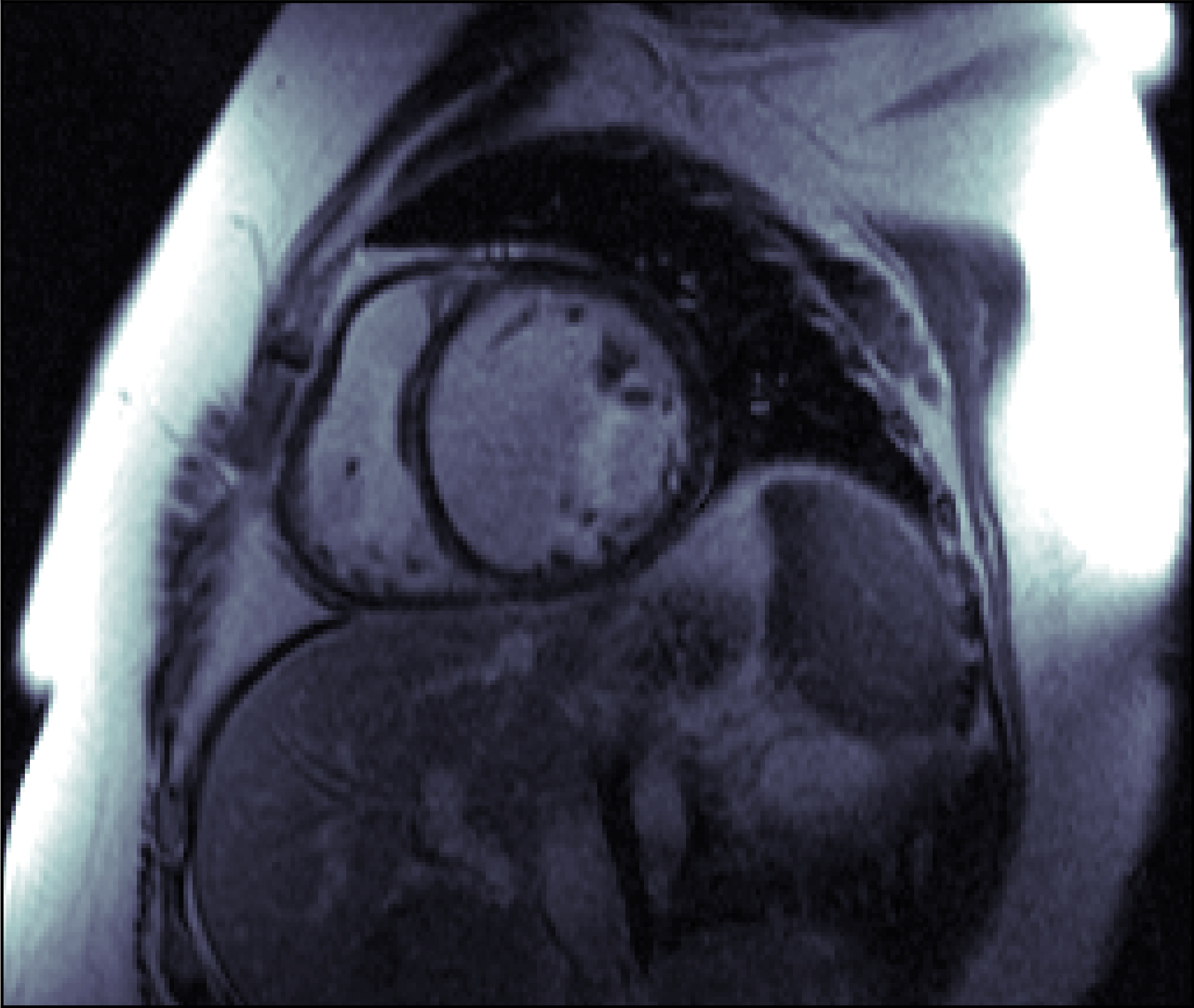

Supplement: S1 Dataset — (ZIP) [file pcbi.1007421.s001.zip › supplementary_segmented_lgemri_data/raw_data/07_04952/66_COL_20070515110747.png]

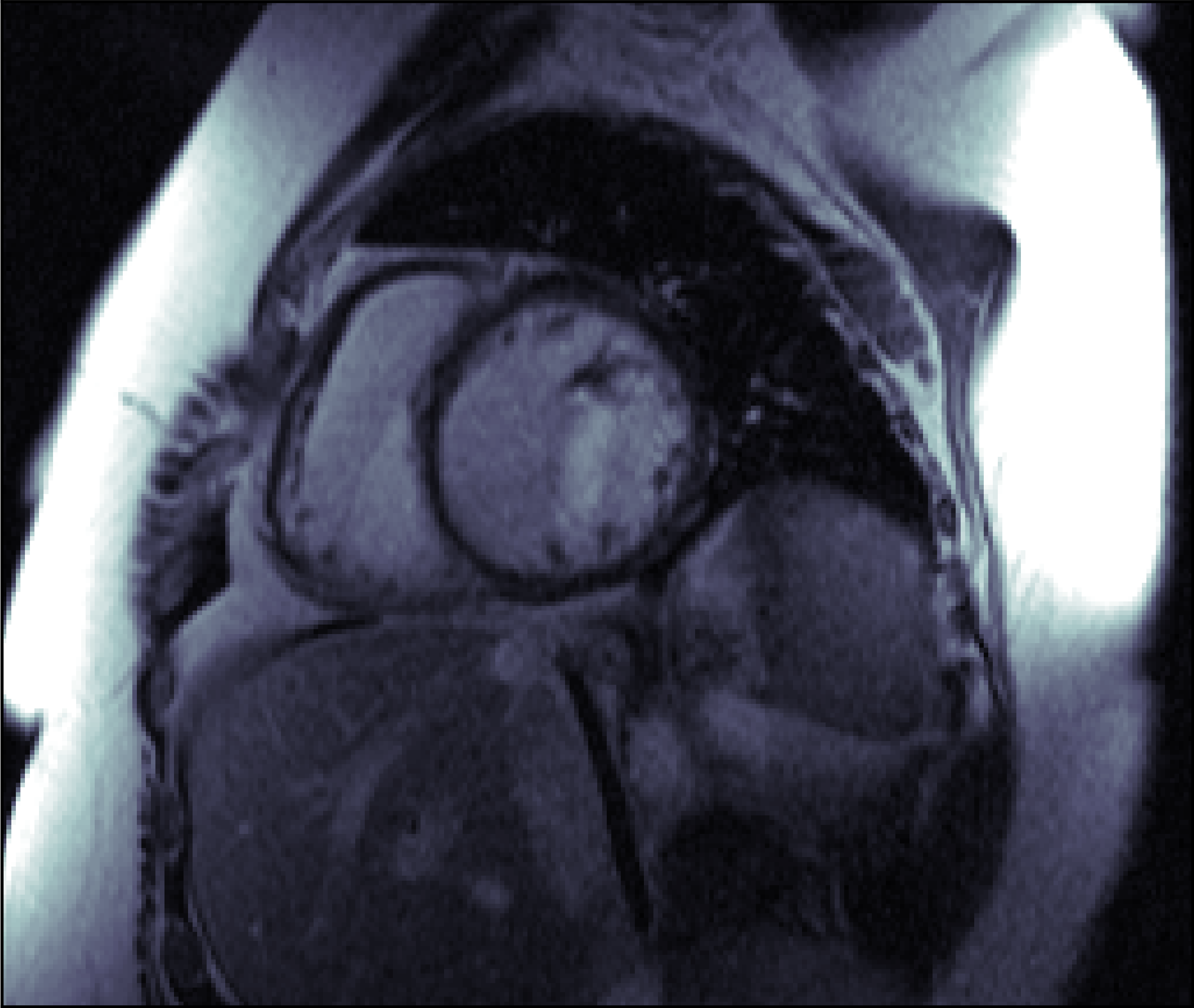

Supplement: S1 Dataset — (ZIP) [file pcbi.1007421.s001.zip › supplementary_segmented_lgemri_data/raw_data/07_04952/56_COL_20070515110706.png]

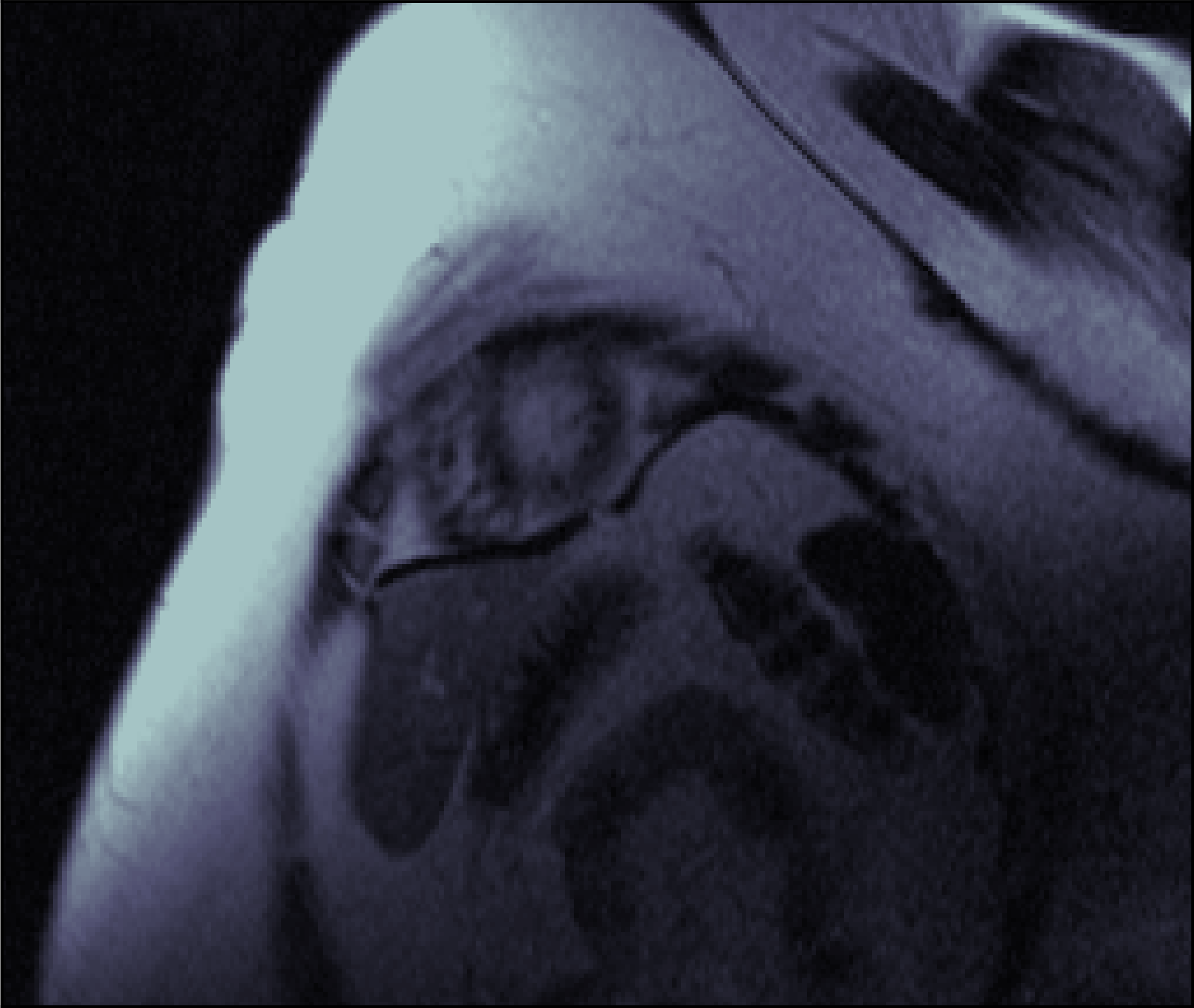

Supplement: S1 Dataset — (ZIP) [file pcbi.1007421.s001.zip › supplementary_segmented_lgemri_data/raw_data/07_04952/137_COL_20070515111245.png]

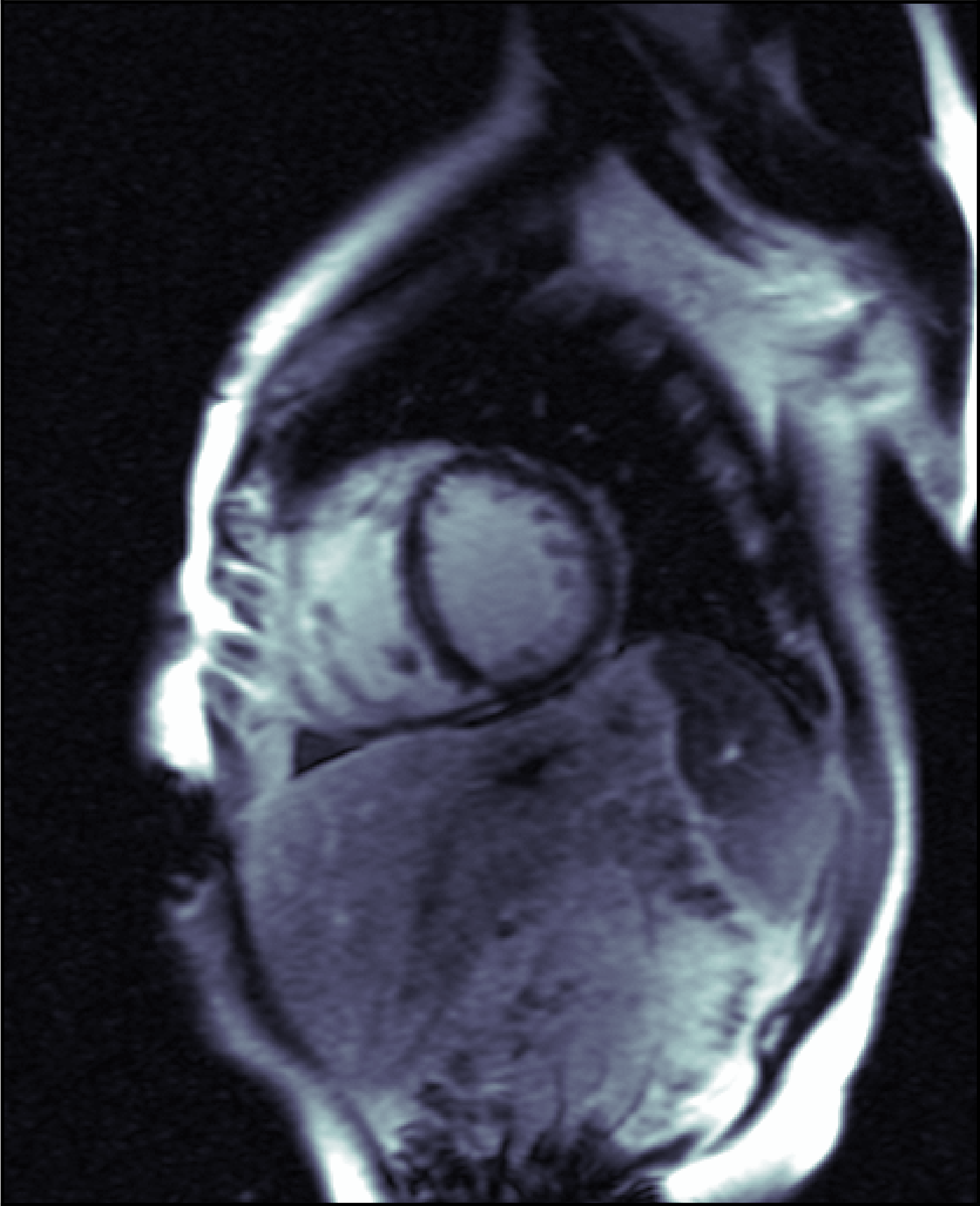

Supplement: S1 Dataset — (ZIP) [file pcbi.1007421.s001.zip › supplementary_segmented_lgemri_data/raw_data/07_01148/95_ROW_20070717092017.png]

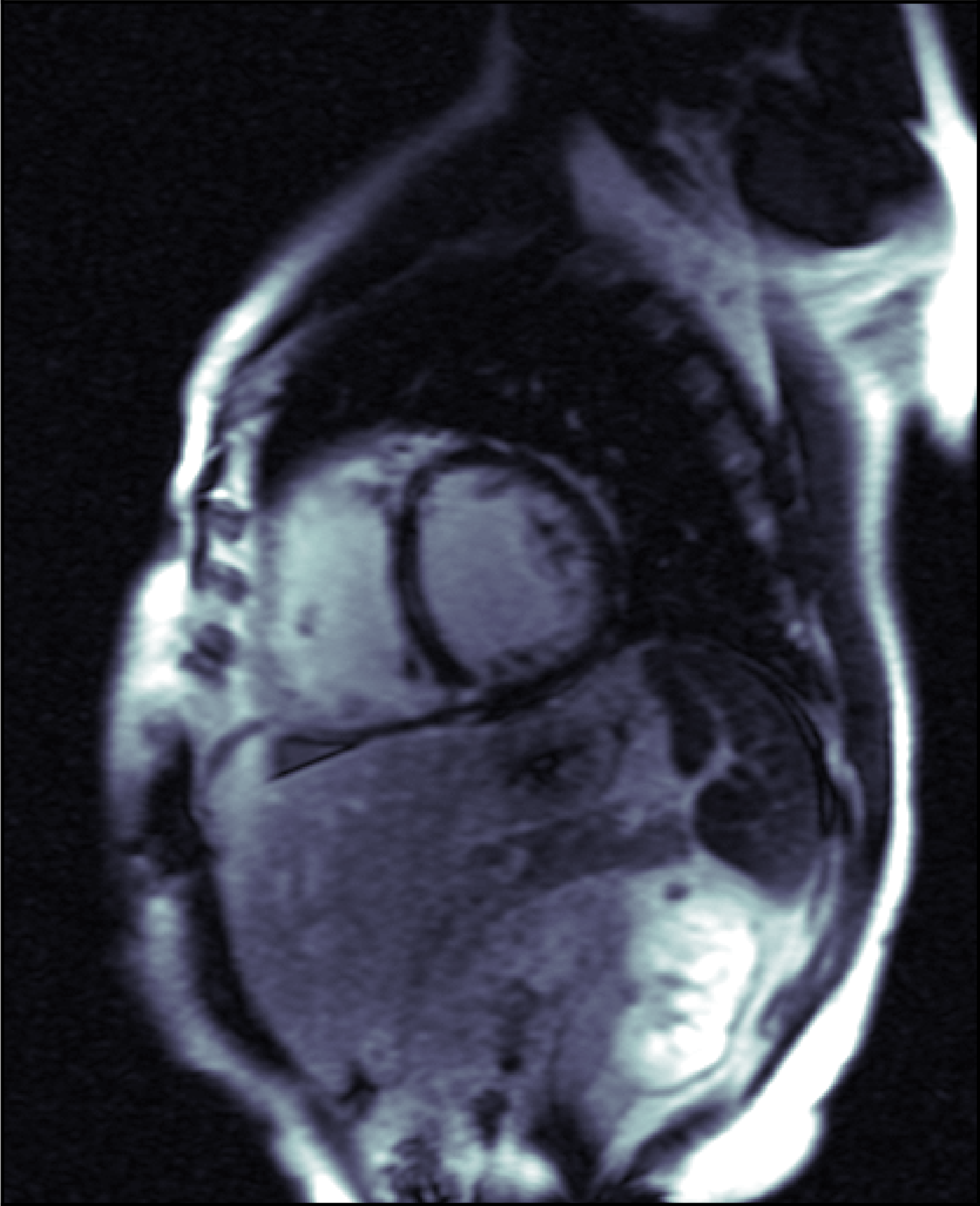

Supplement: S1 Dataset — (ZIP) [file pcbi.1007421.s001.zip › supplementary_segmented_lgemri_data/raw_data/07_01148/84_ROW_20070717091917.png]

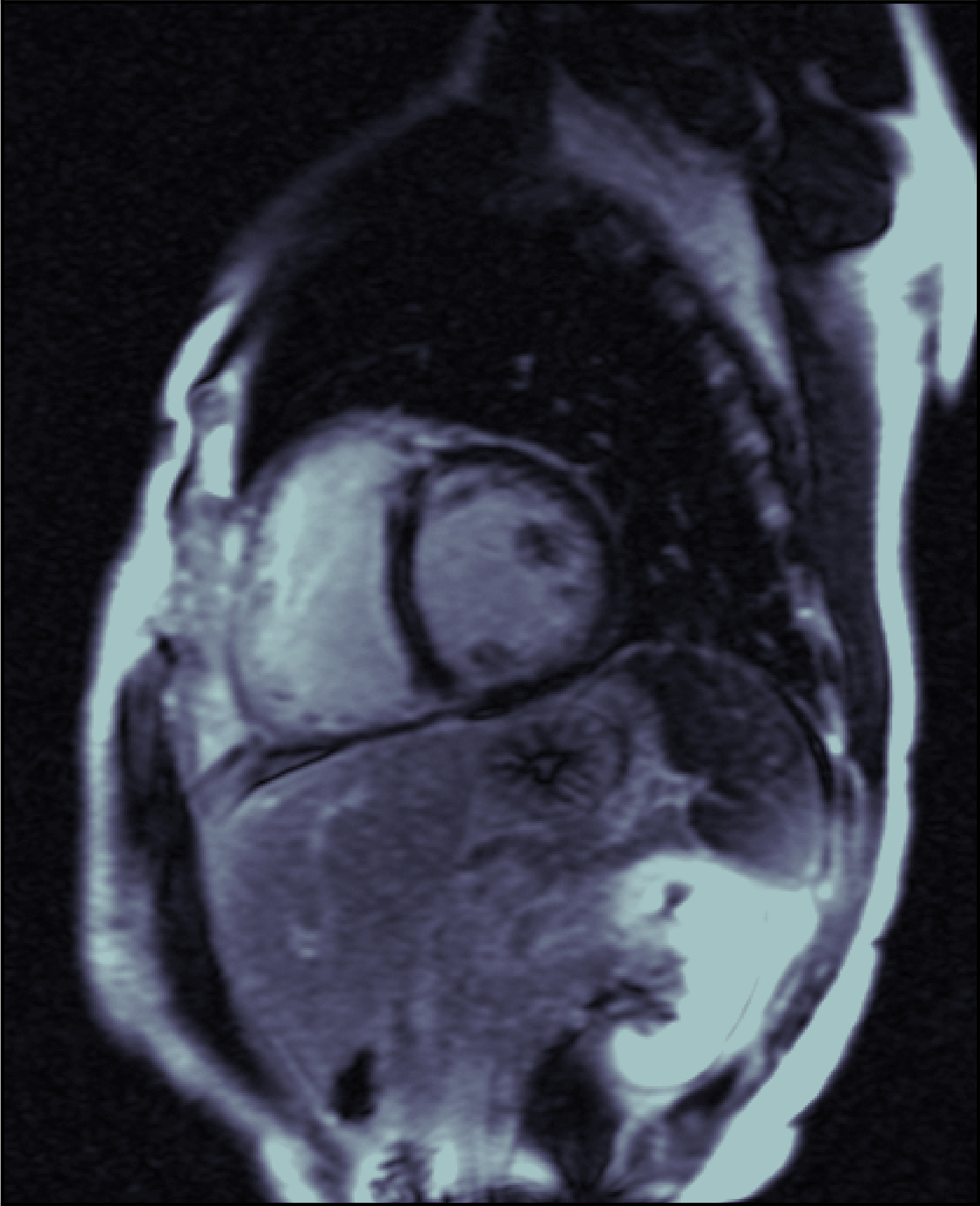

Supplement: S1 Dataset — (ZIP) [file pcbi.1007421.s001.zip › supplementary_segmented_lgemri_data/raw_data/07_01148/74_ROW_20070717091851.png]

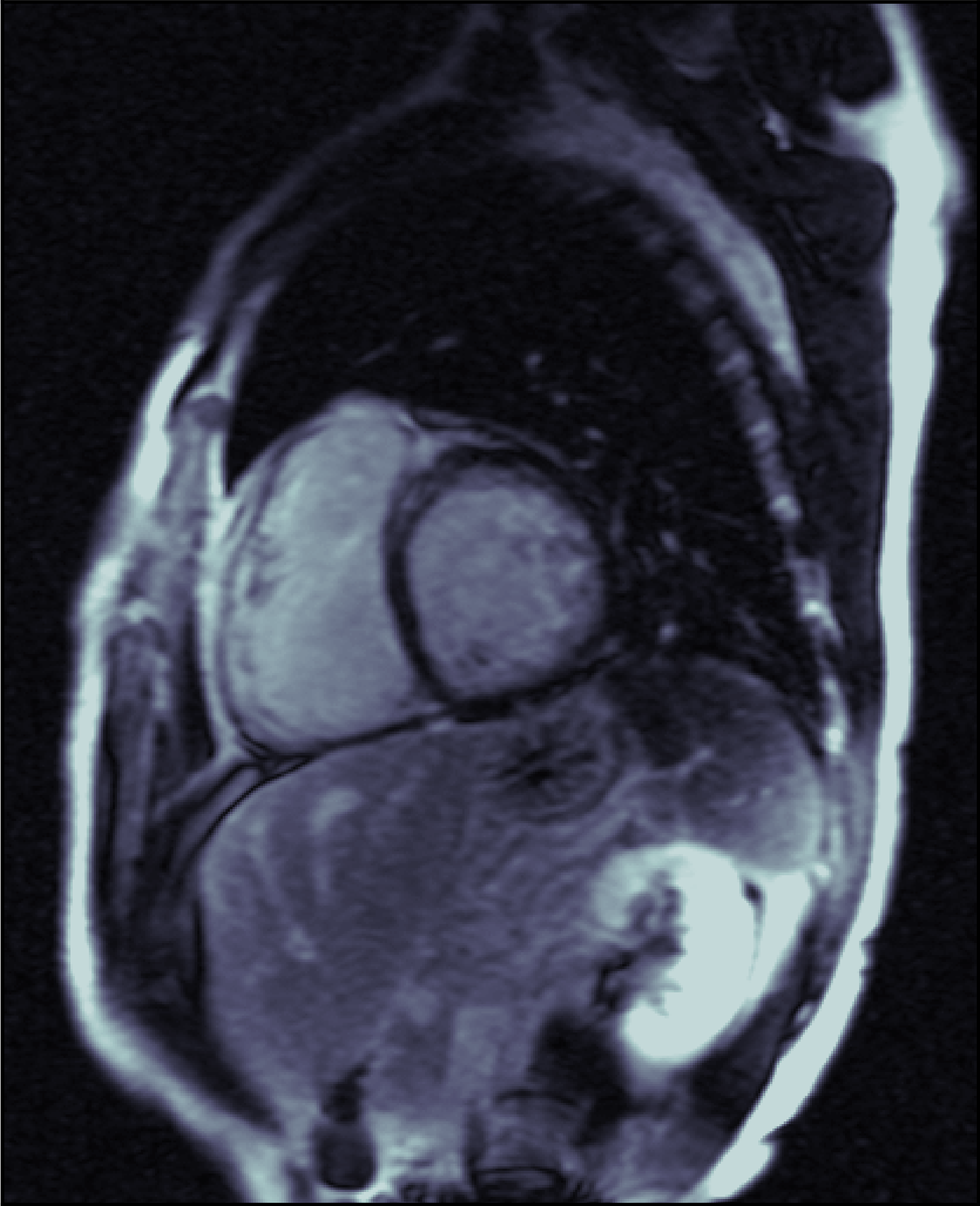

Supplement: S1 Dataset — (ZIP) [file pcbi.1007421.s001.zip › supplementary_segmented_lgemri_data/raw_data/07_01148/65_ROW_20070717091821.png]

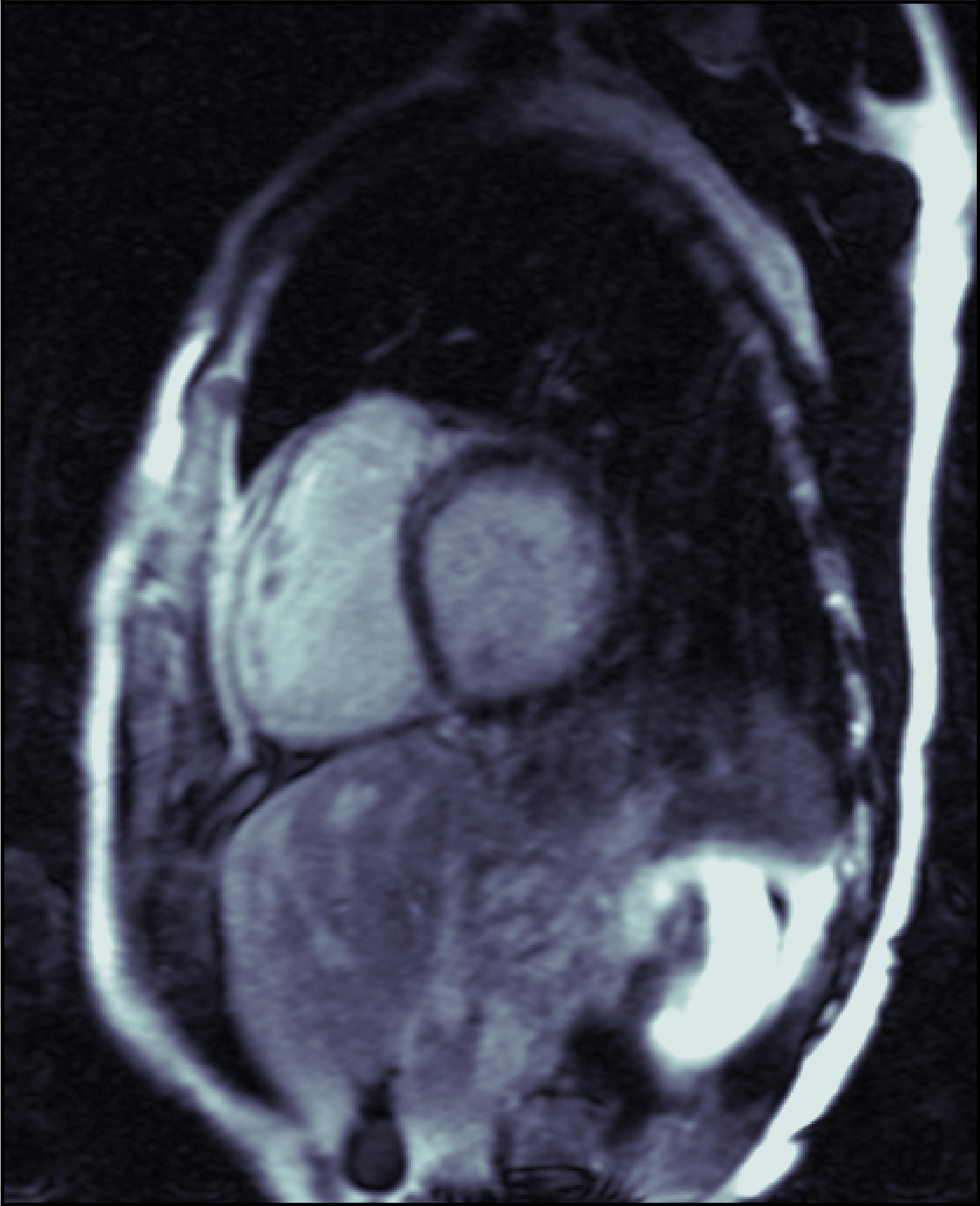

Supplement: S1 Dataset — (ZIP) [file pcbi.1007421.s001.zip › supplementary_segmented_lgemri_data/raw_data/07_01148/65_ROW_20070717091754.png]

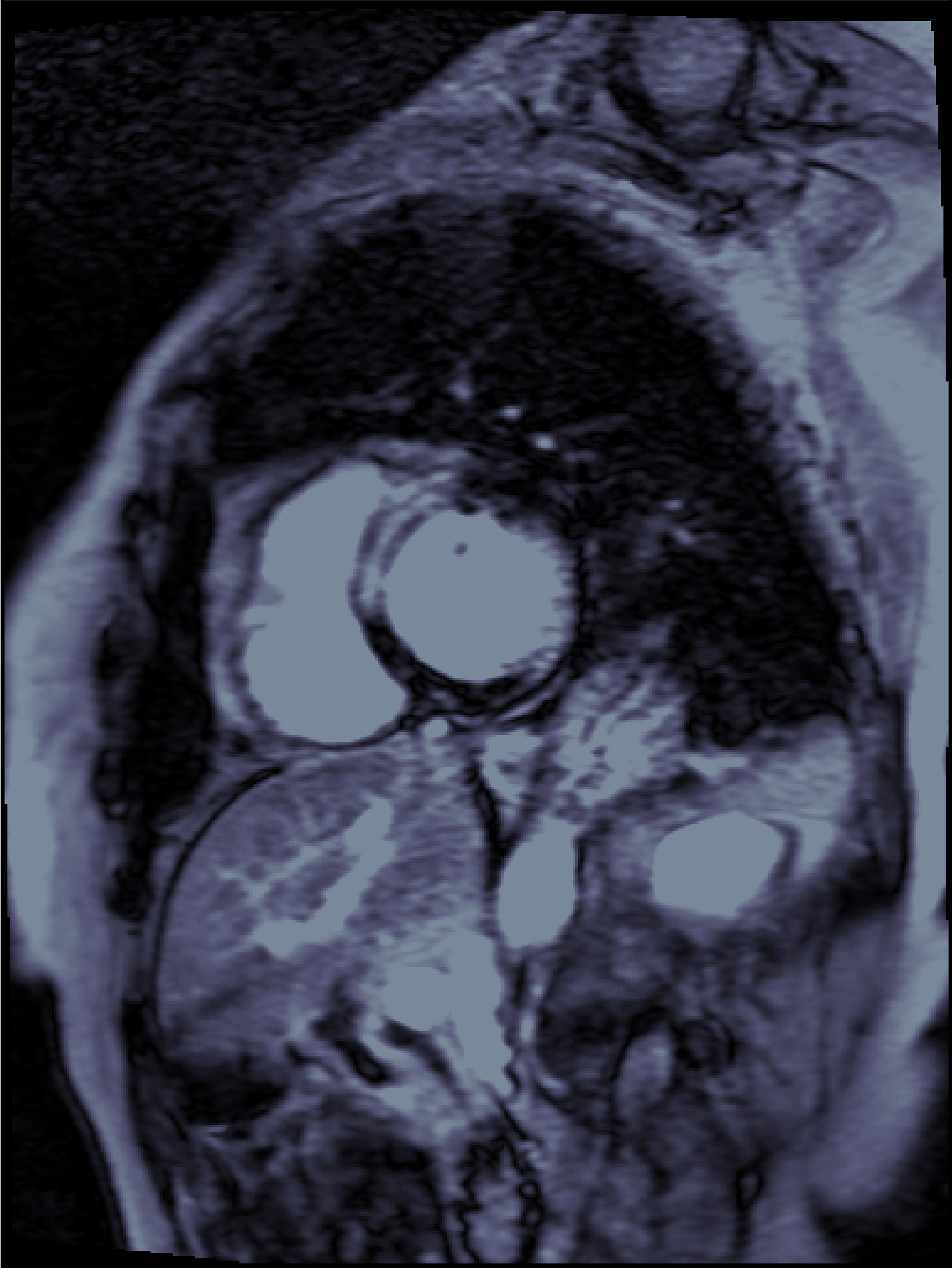

Supplement: S1 Dataset — (ZIP) [file pcbi.1007421.s001.zip › supplementary_segmented_lgemri_data/raw_data/05_23181/-36_ROW_20100713125610.png]

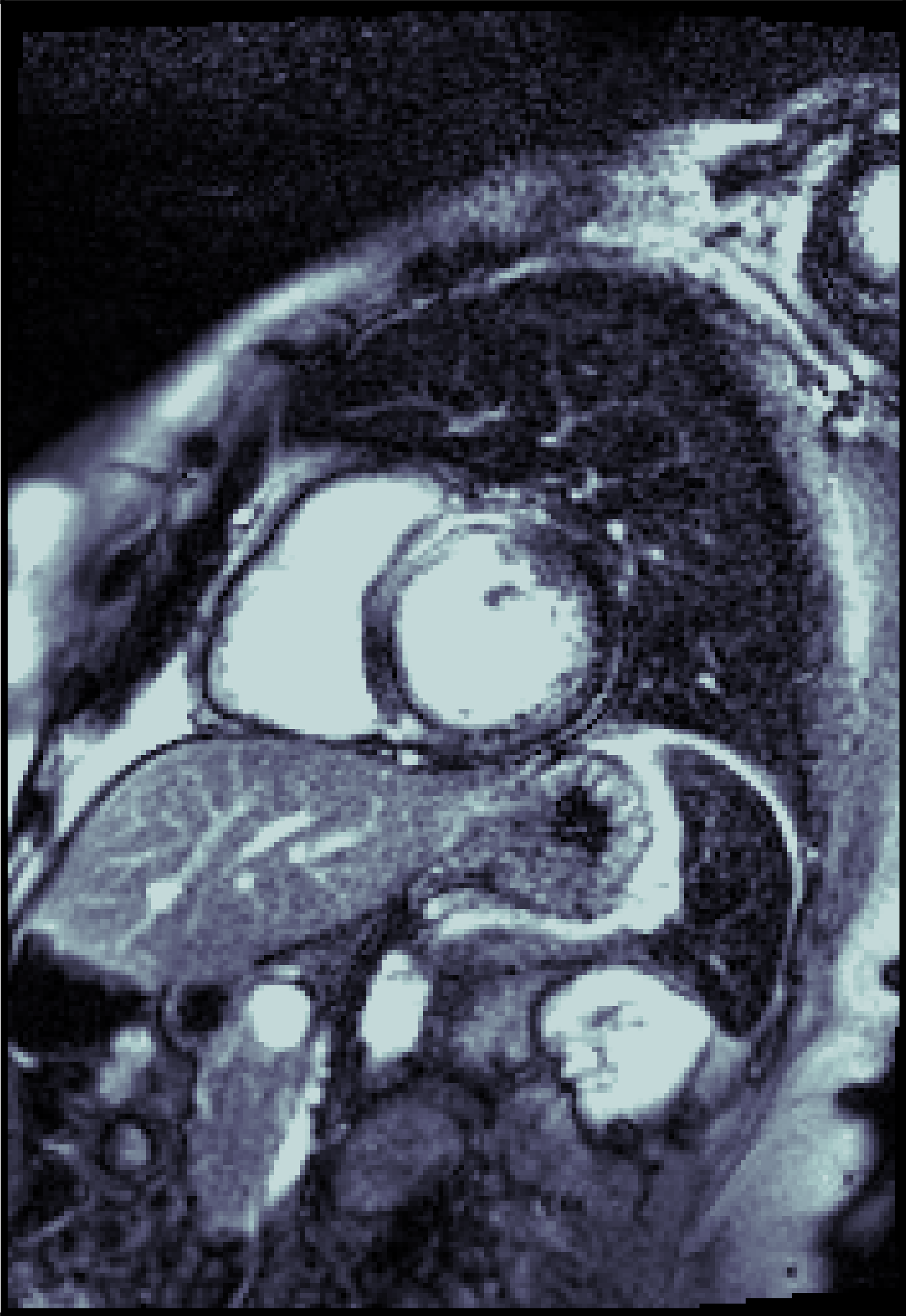

Supplement: S1 Dataset — (ZIP) [file pcbi.1007421.s001.zip › supplementary_segmented_lgemri_data/raw_data/05_23181/-45_ROW_20100713125212.png]

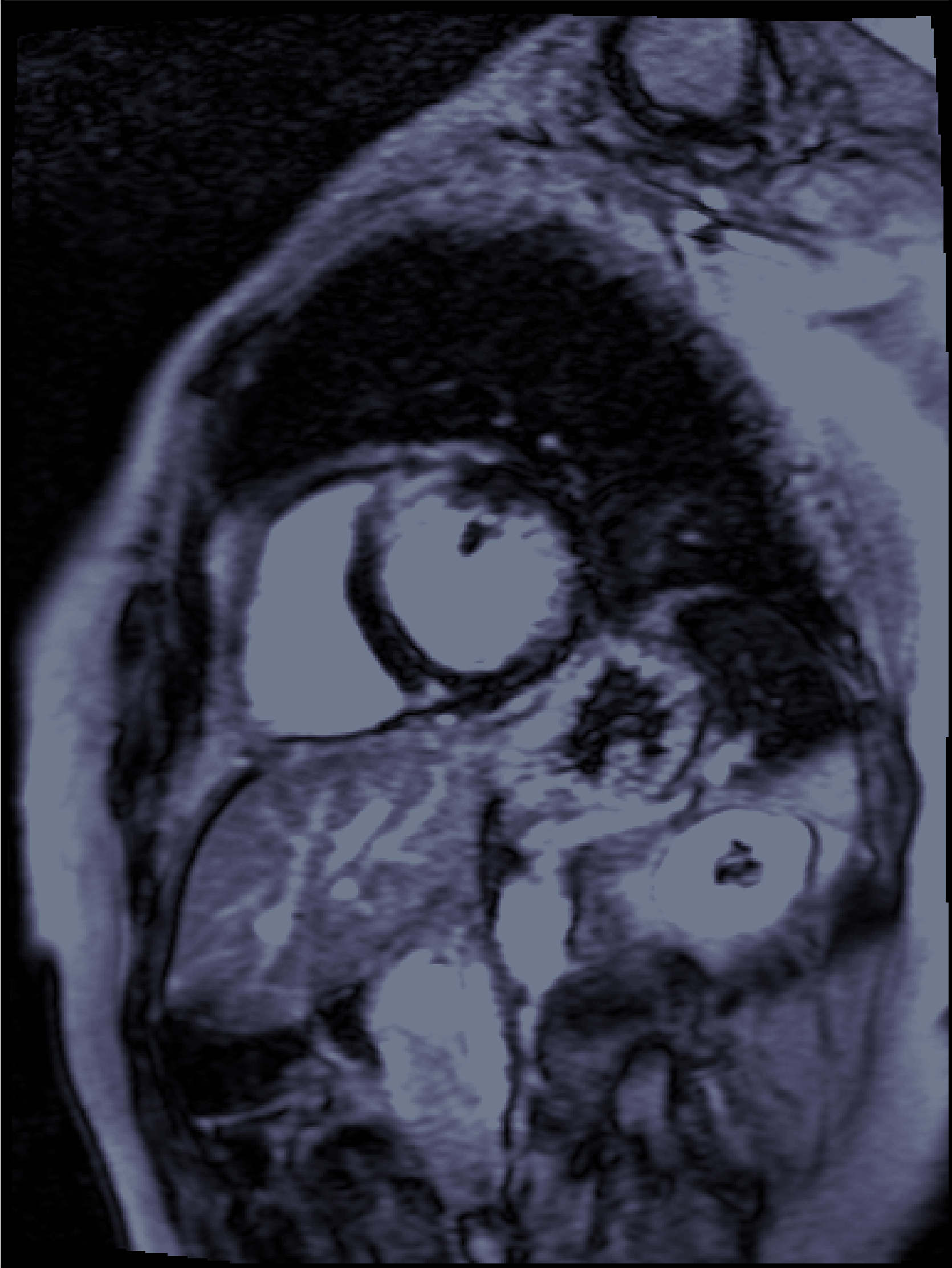

Supplement: S1 Dataset — (ZIP) [file pcbi.1007421.s001.zip › supplementary_segmented_lgemri_data/raw_data/05_23181/-46_ROW_20100713125635.png]

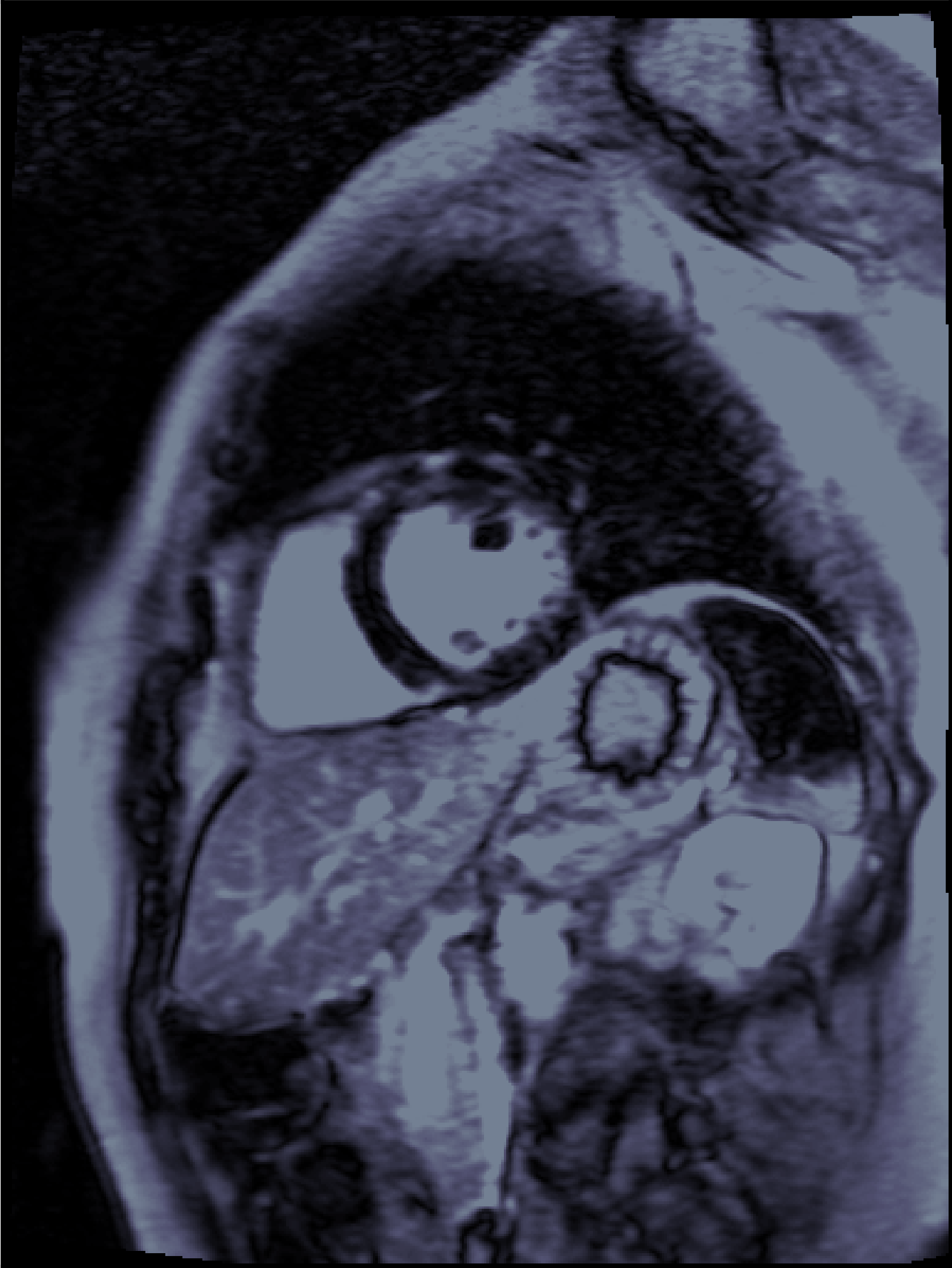

Supplement: S1 Dataset — (ZIP) [file pcbi.1007421.s001.zip › supplementary_segmented_lgemri_data/raw_data/05_23181/-56_ROW_20100713125705.png]

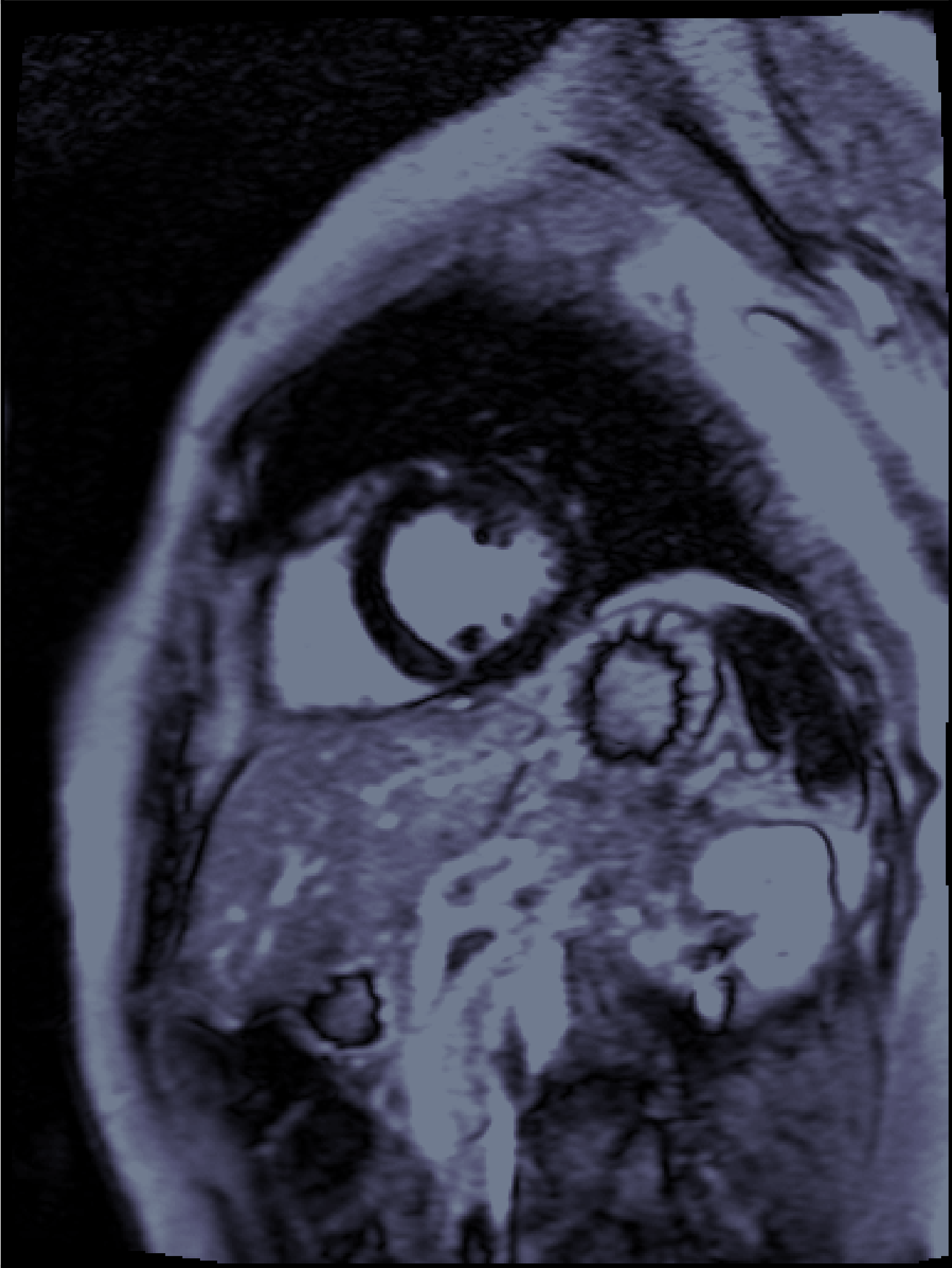

Supplement: S1 Dataset — (ZIP) [file pcbi.1007421.s001.zip › supplementary_segmented_lgemri_data/raw_data/05_23181/-66_ROW_20100713125736.png]

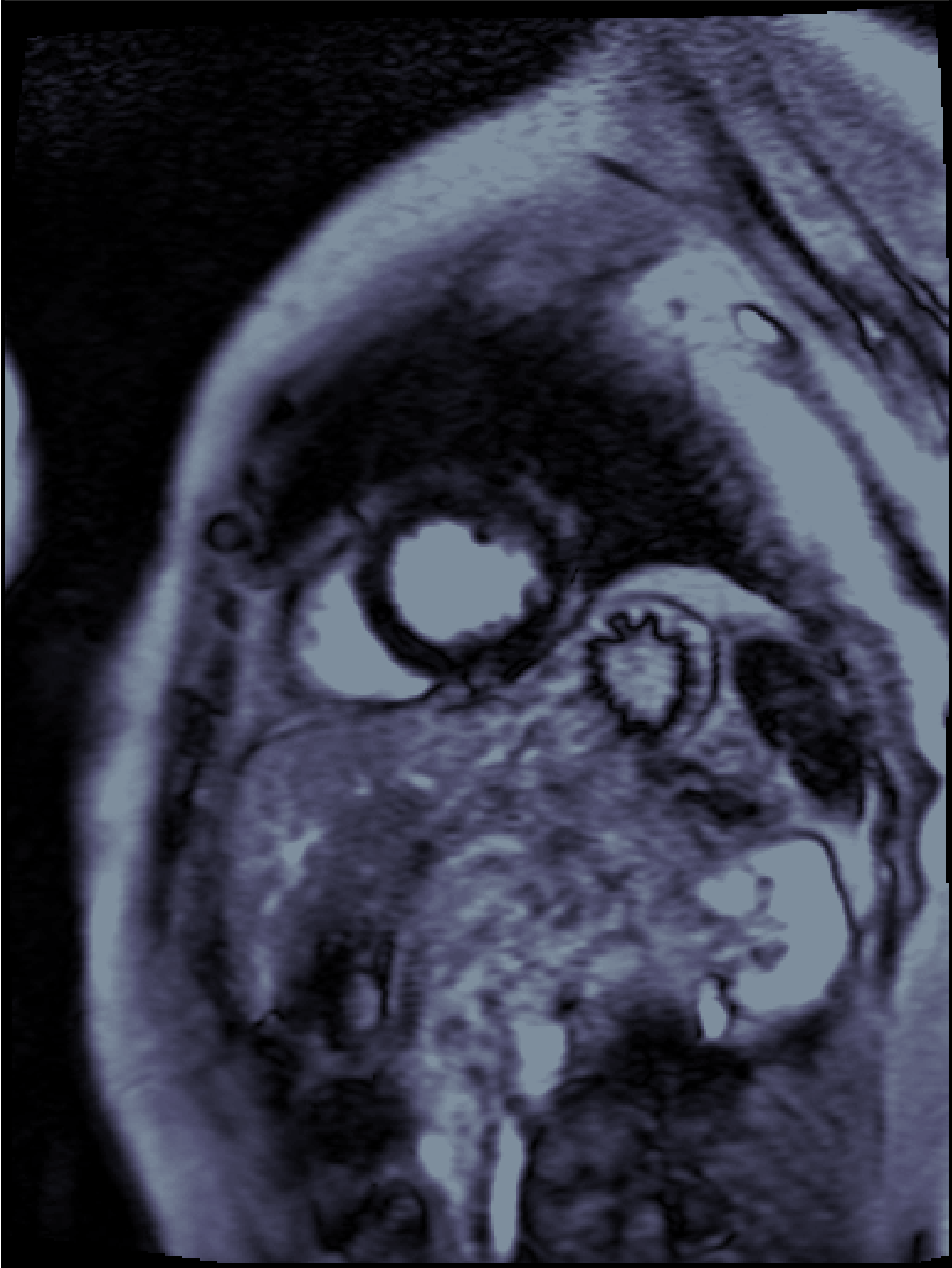

Supplement: S1 Dataset — (ZIP) [file pcbi.1007421.s001.zip › supplementary_segmented_lgemri_data/raw_data/05_23181/-76_ROW_20100713125802.png]

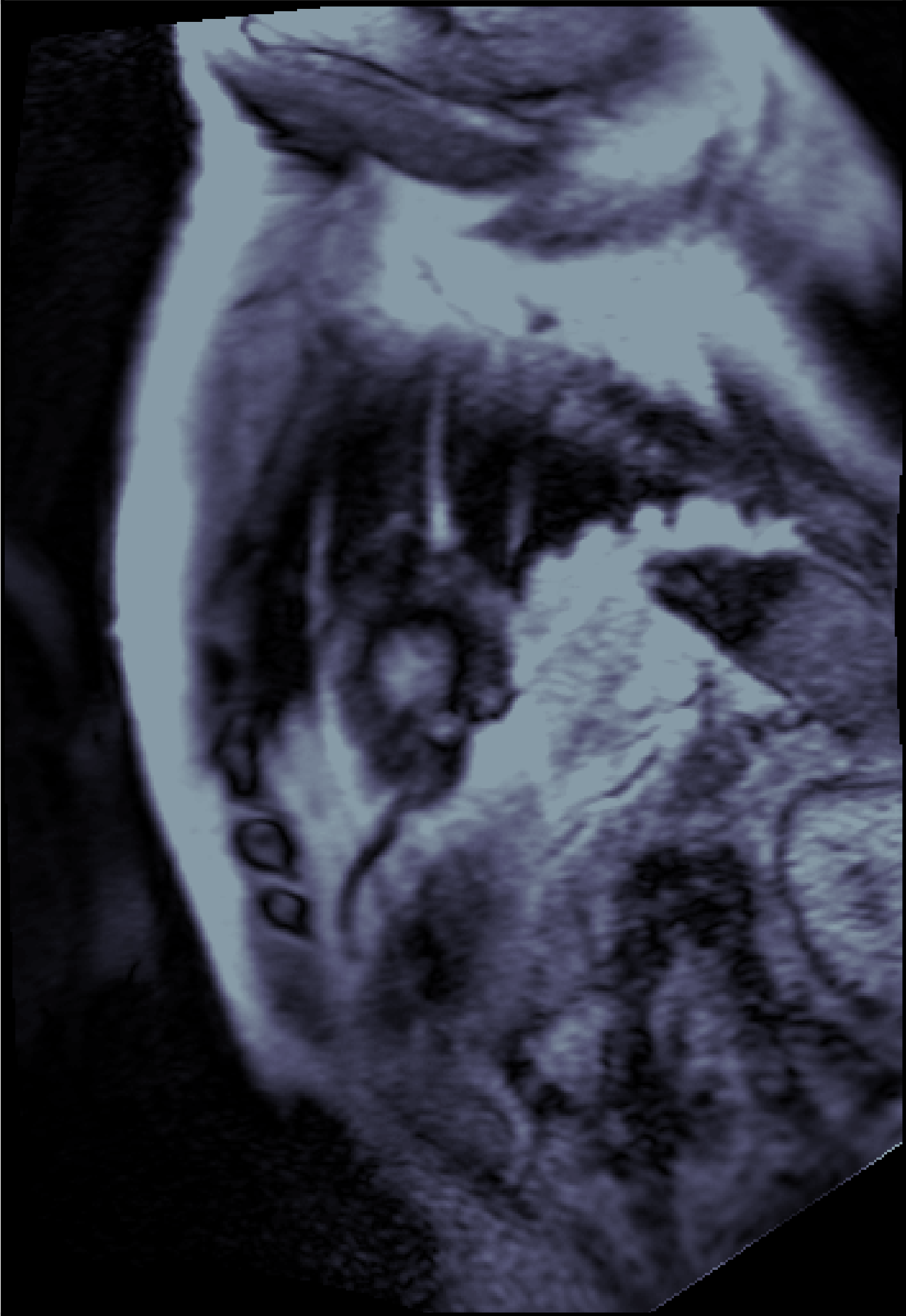

Supplement: S1 Dataset — (ZIP) [file pcbi.1007421.s001.zip › supplementary_segmented_lgemri_data/raw_data/05_14699/132_ROW_20091106154439.png]

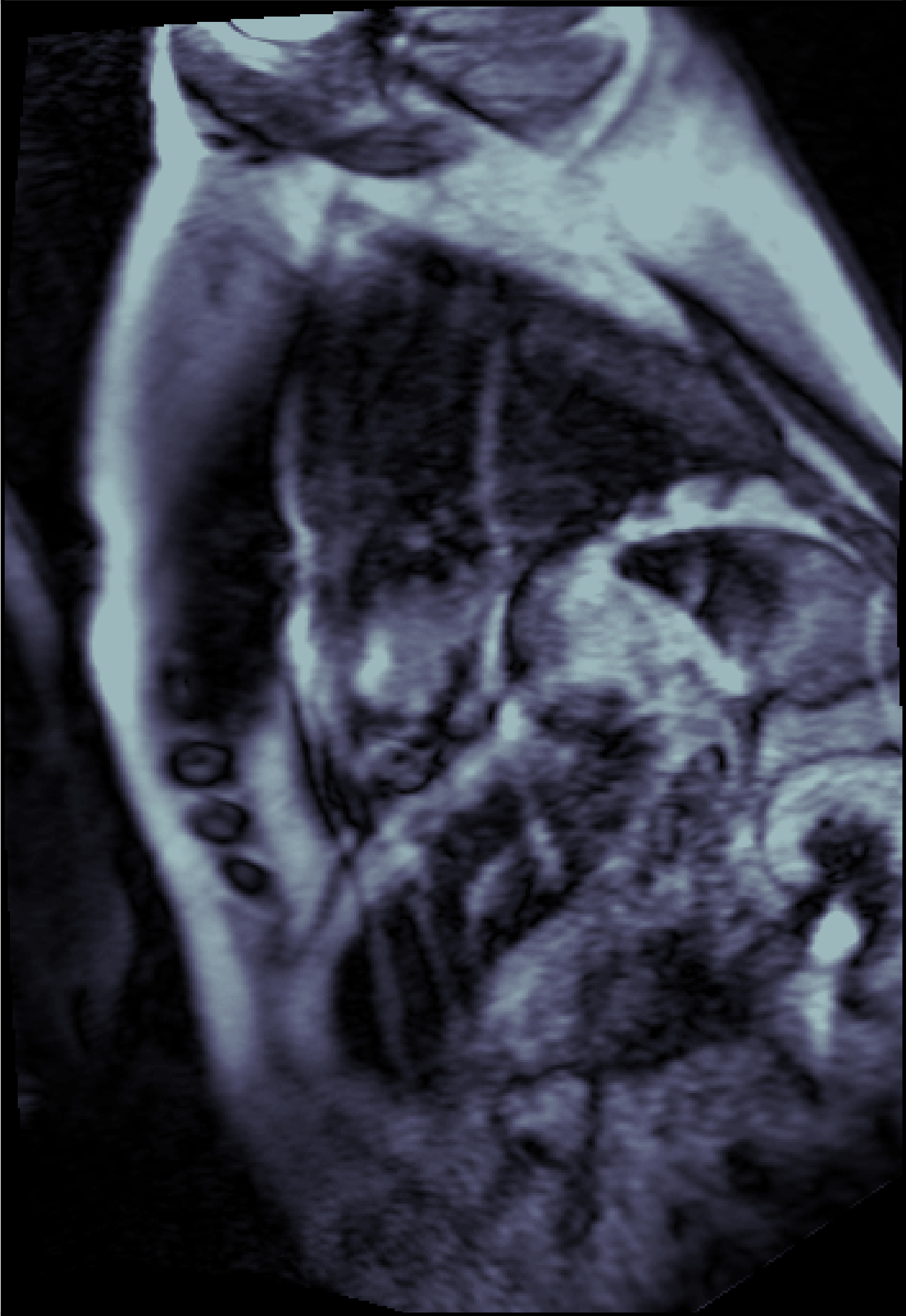

Supplement: S1 Dataset — (ZIP) [file pcbi.1007421.s001.zip › supplementary_segmented_lgemri_data/raw_data/05_14699/112_ROW_20091106154328.png]

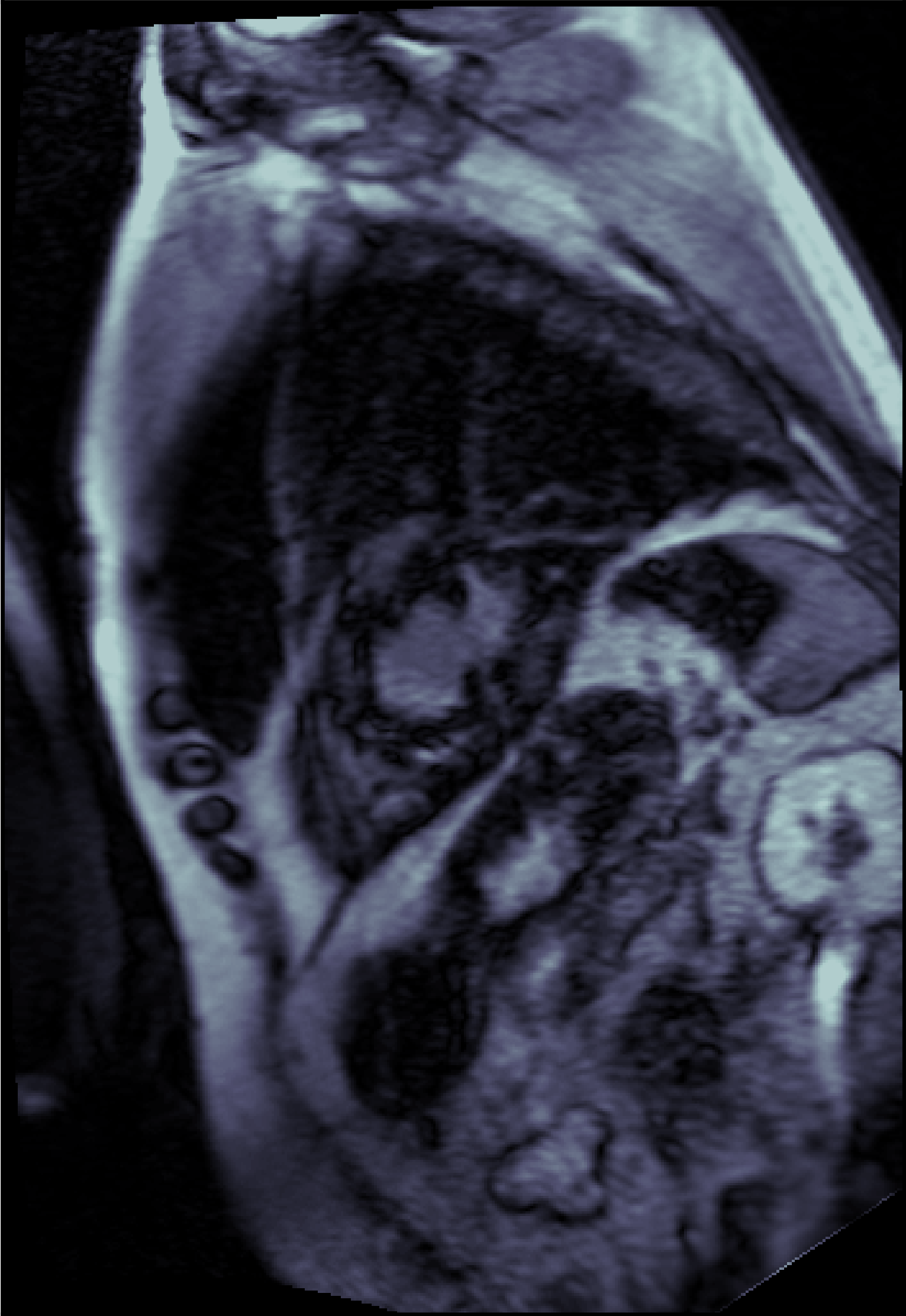

Supplement: S1 Dataset — (ZIP) [file pcbi.1007421.s001.zip › supplementary_segmented_lgemri_data/raw_data/05_14699/102_ROW_20091106154246.png]

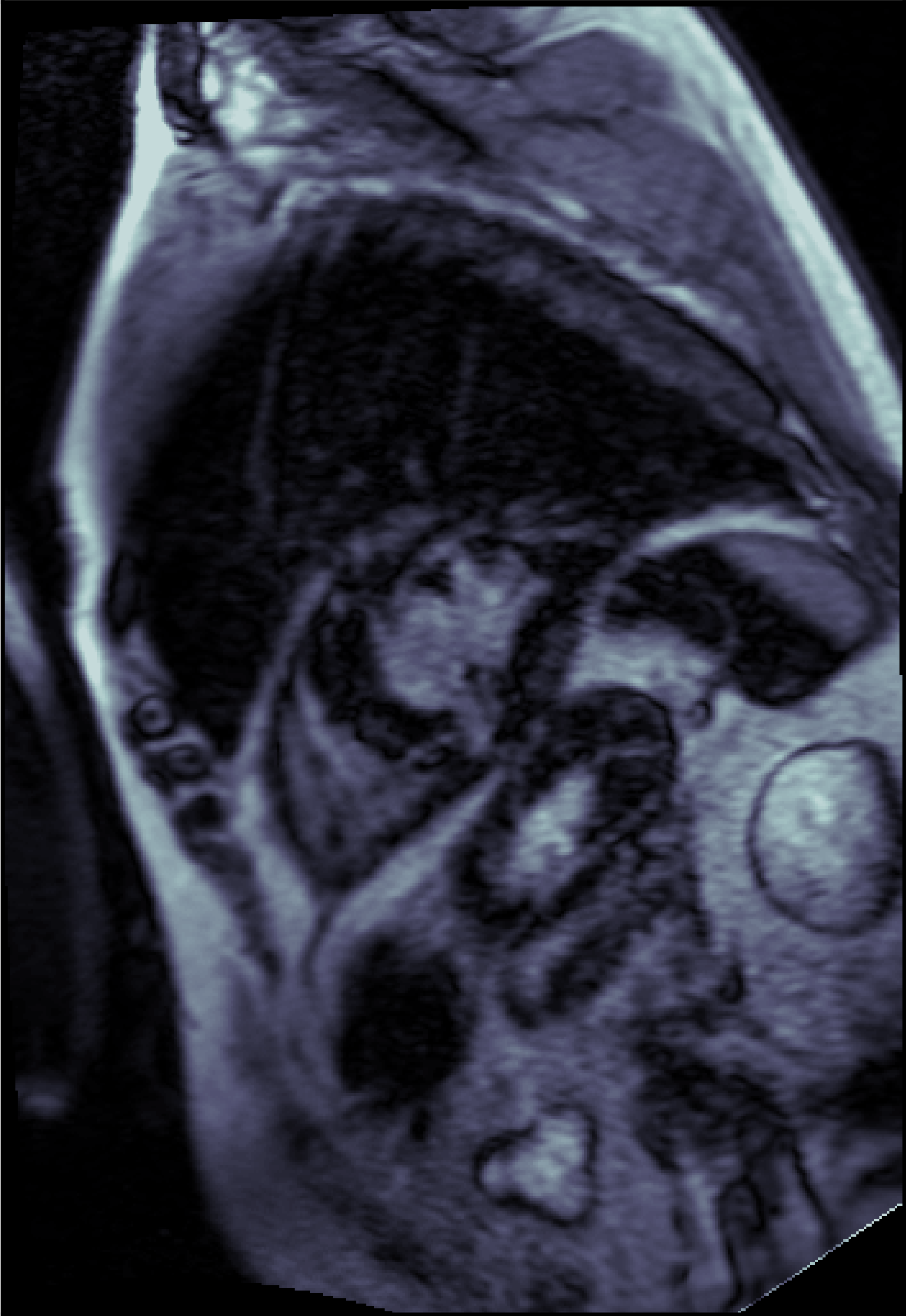

Supplement: S1 Dataset — (ZIP) [file pcbi.1007421.s001.zip › supplementary_segmented_lgemri_data/raw_data/05_14699/92_ROW_20091106154206.png]

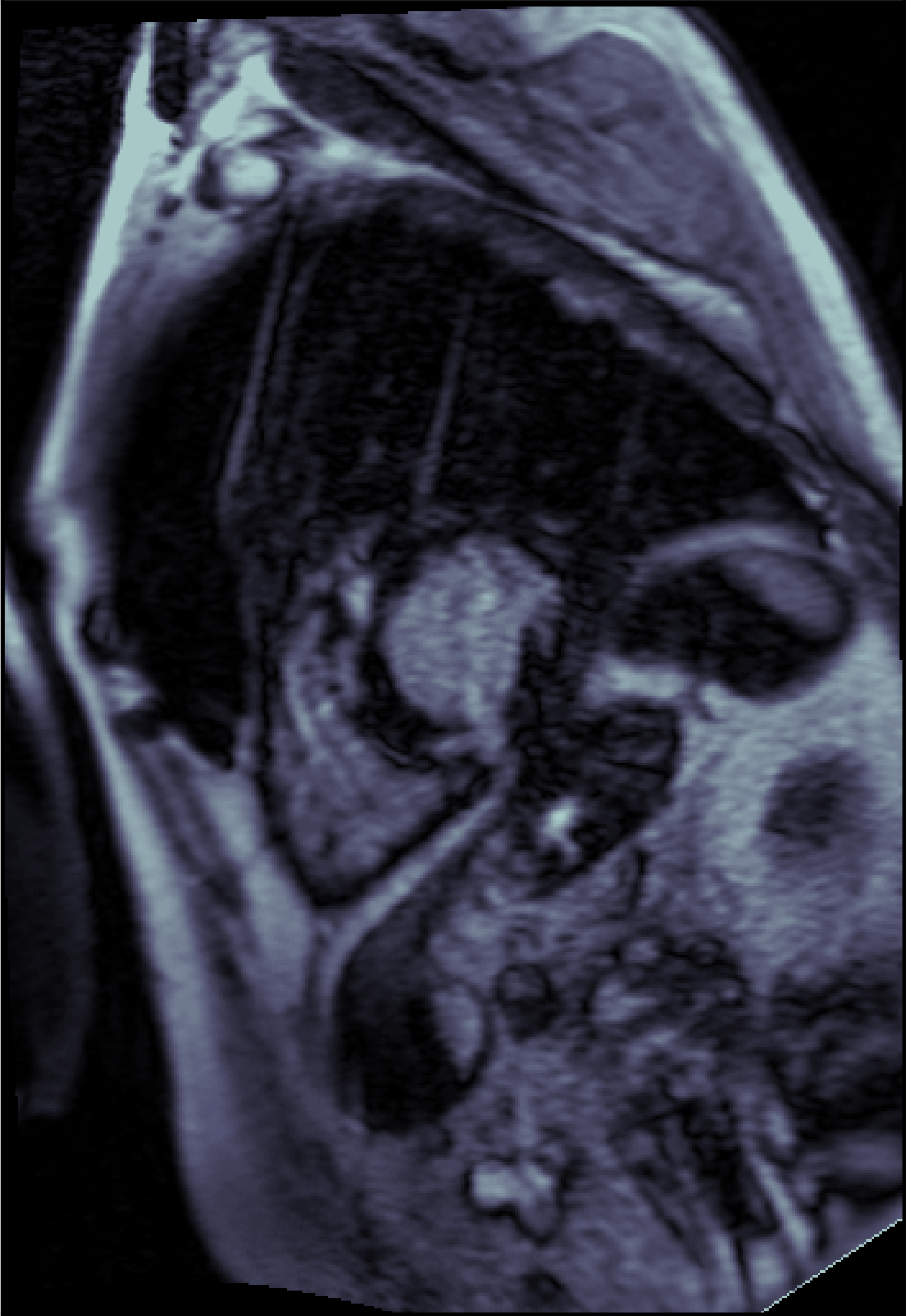

Supplement: S1 Dataset — (ZIP) [file pcbi.1007421.s001.zip › supplementary_segmented_lgemri_data/raw_data/05_14699/82_ROW_20091106154143.png]

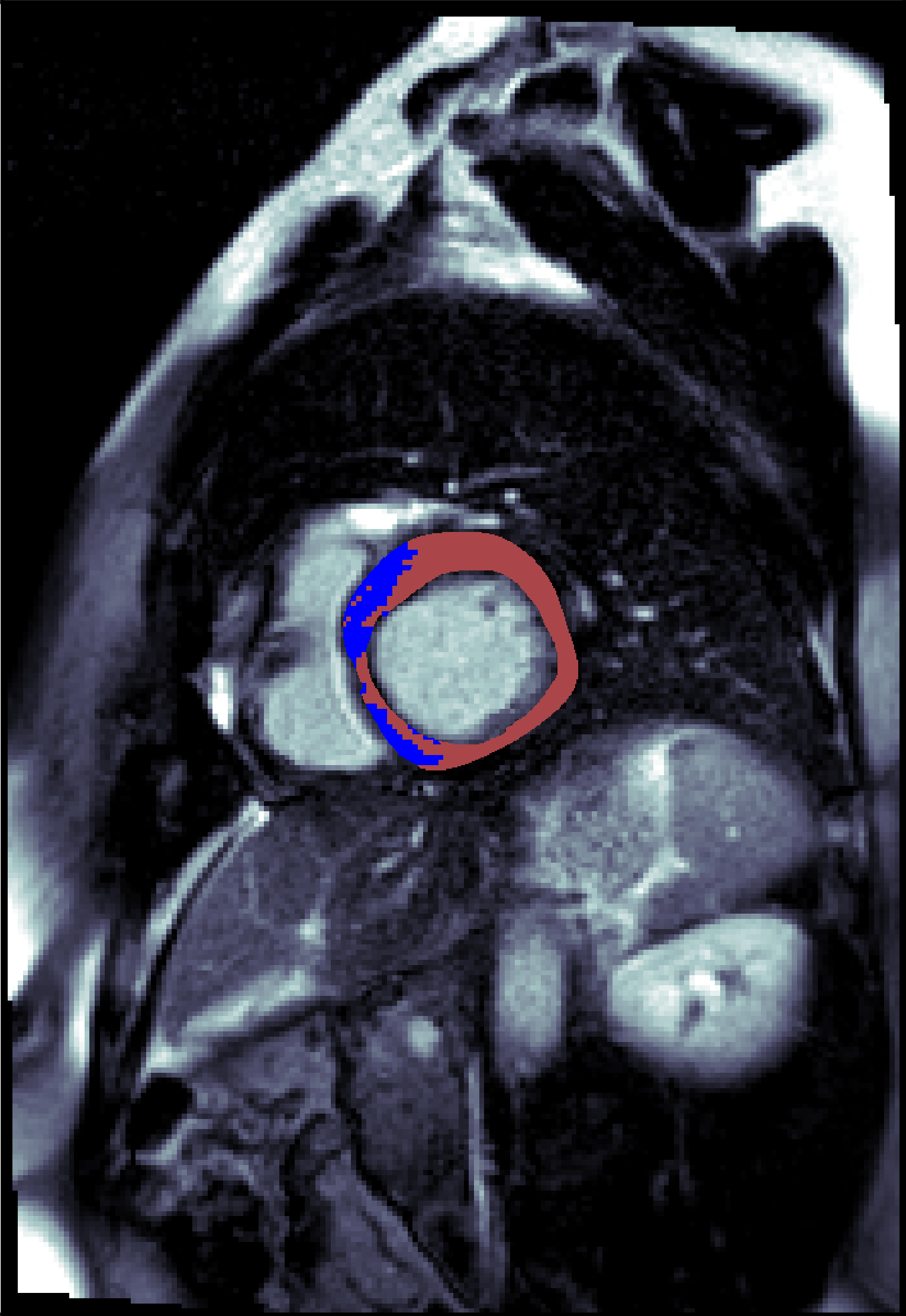

Supplement: S1 Dataset — (ZIP) [file pcbi.1007421.s001.zip › supplementary_segmented_lgemri_data/segmentations/10_00409/39_ROW_16000101124611.png]

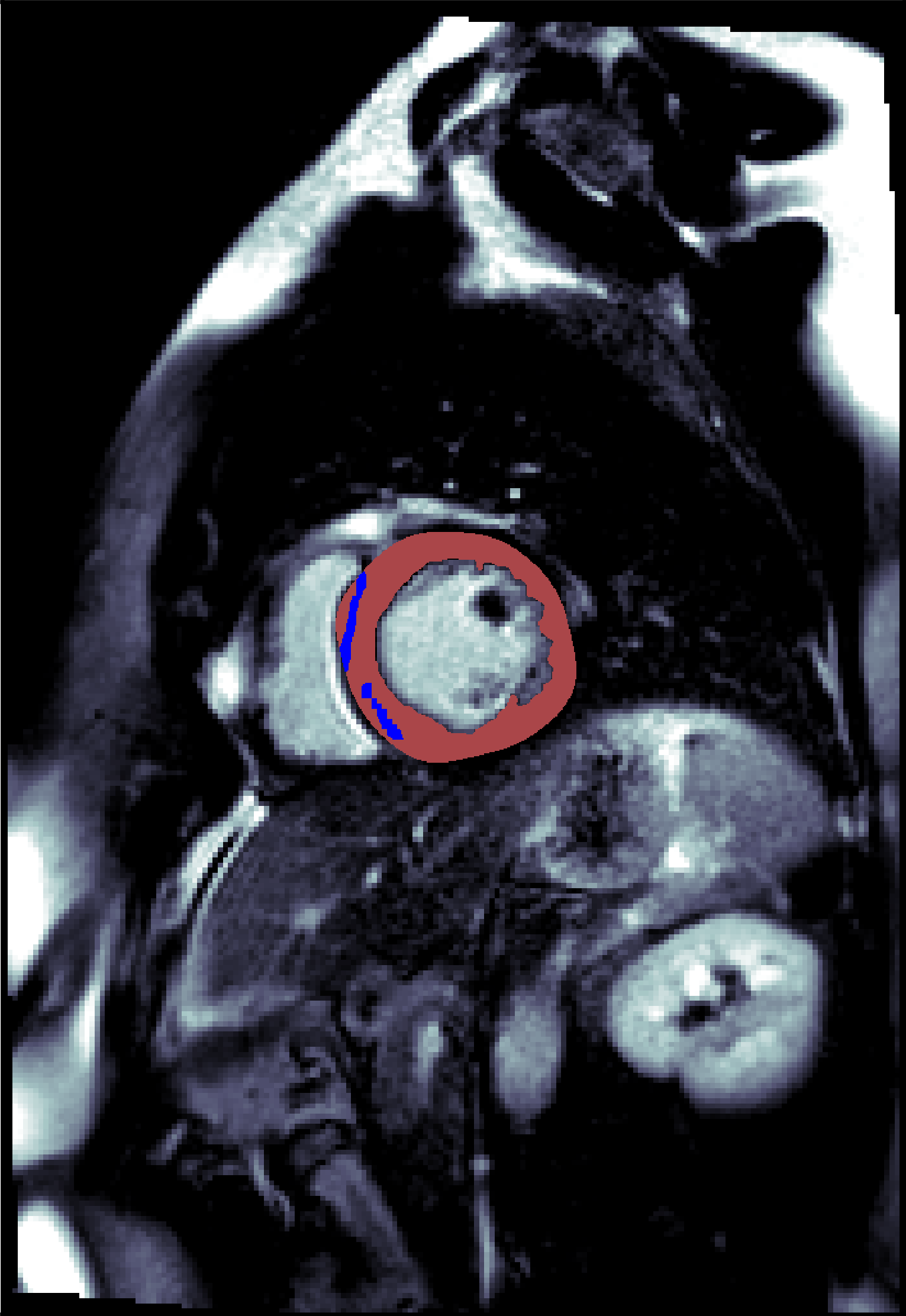

Supplement: S1 Dataset — (ZIP) [file pcbi.1007421.s001.zip › supplementary_segmented_lgemri_data/segmentations/10_00409/49_ROW_16000101124615.png]

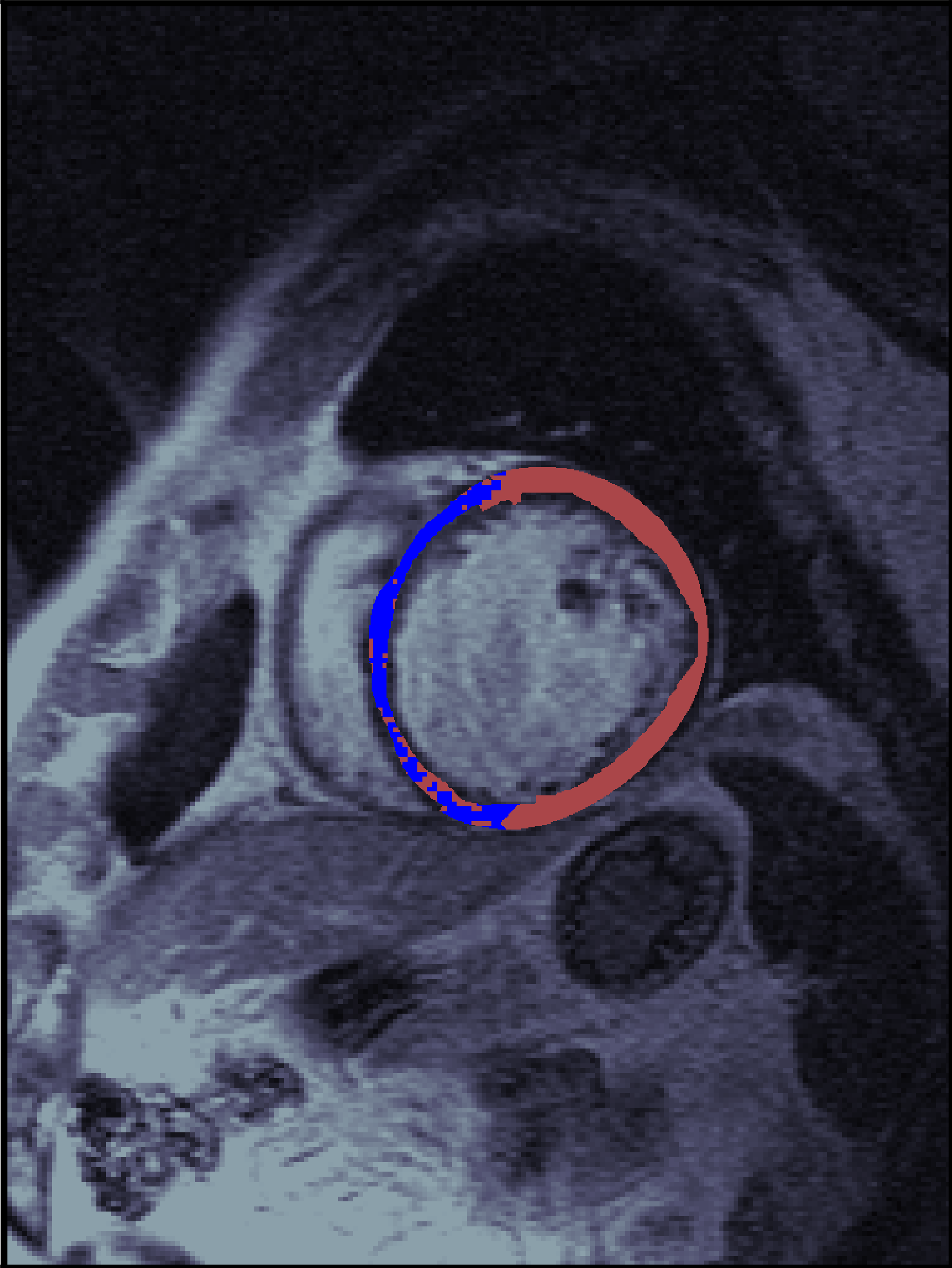

Supplement: S1 Dataset — (ZIP) [file pcbi.1007421.s001.zip › supplementary_segmented_lgemri_data/segmentations/07_18651/-104_ROW_20070822084727.png]

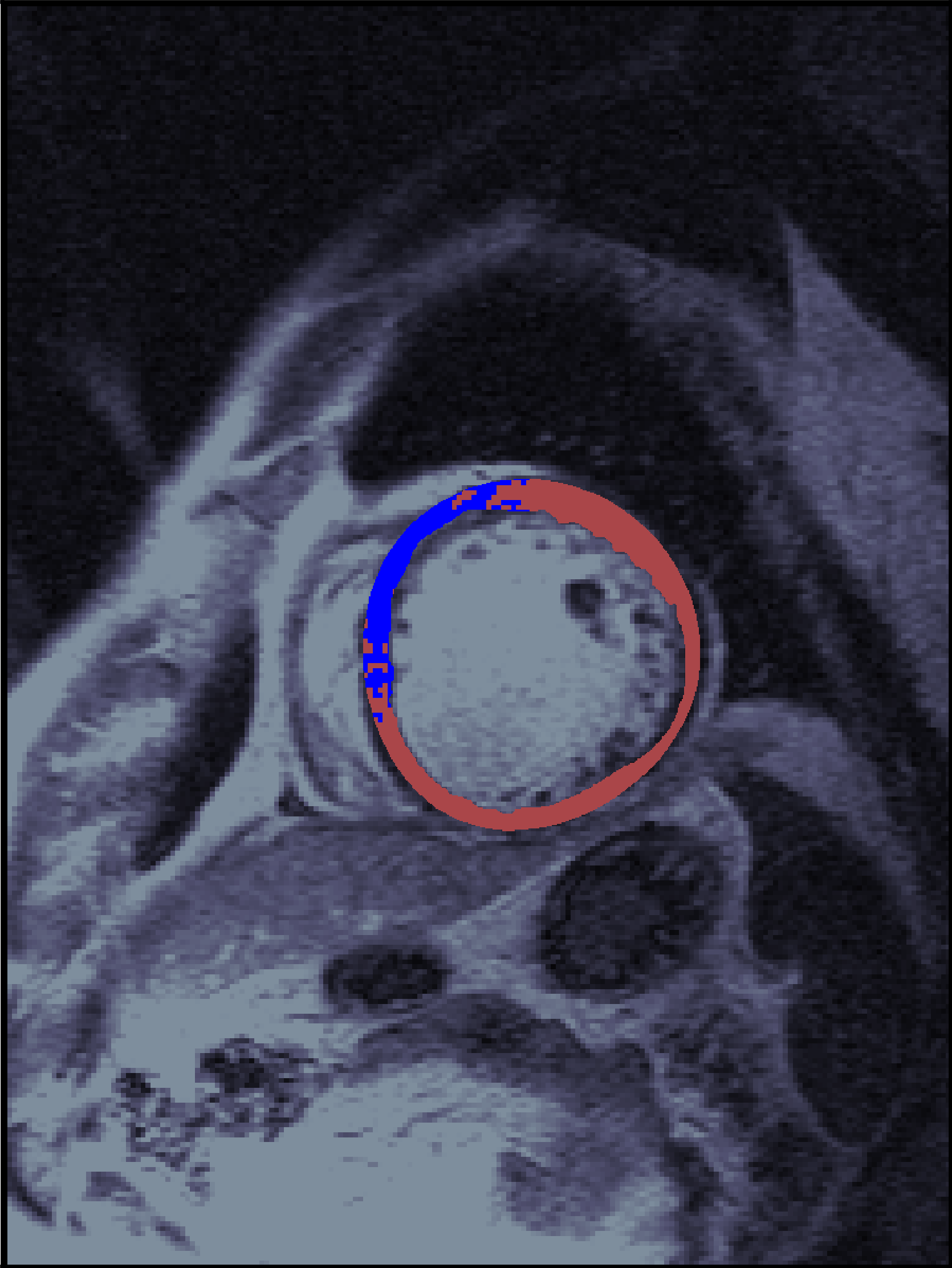

Supplement: S1 Dataset — (ZIP) [file pcbi.1007421.s001.zip › supplementary_segmented_lgemri_data/segmentations/07_18651/-114_ROW_20070822084753.png]

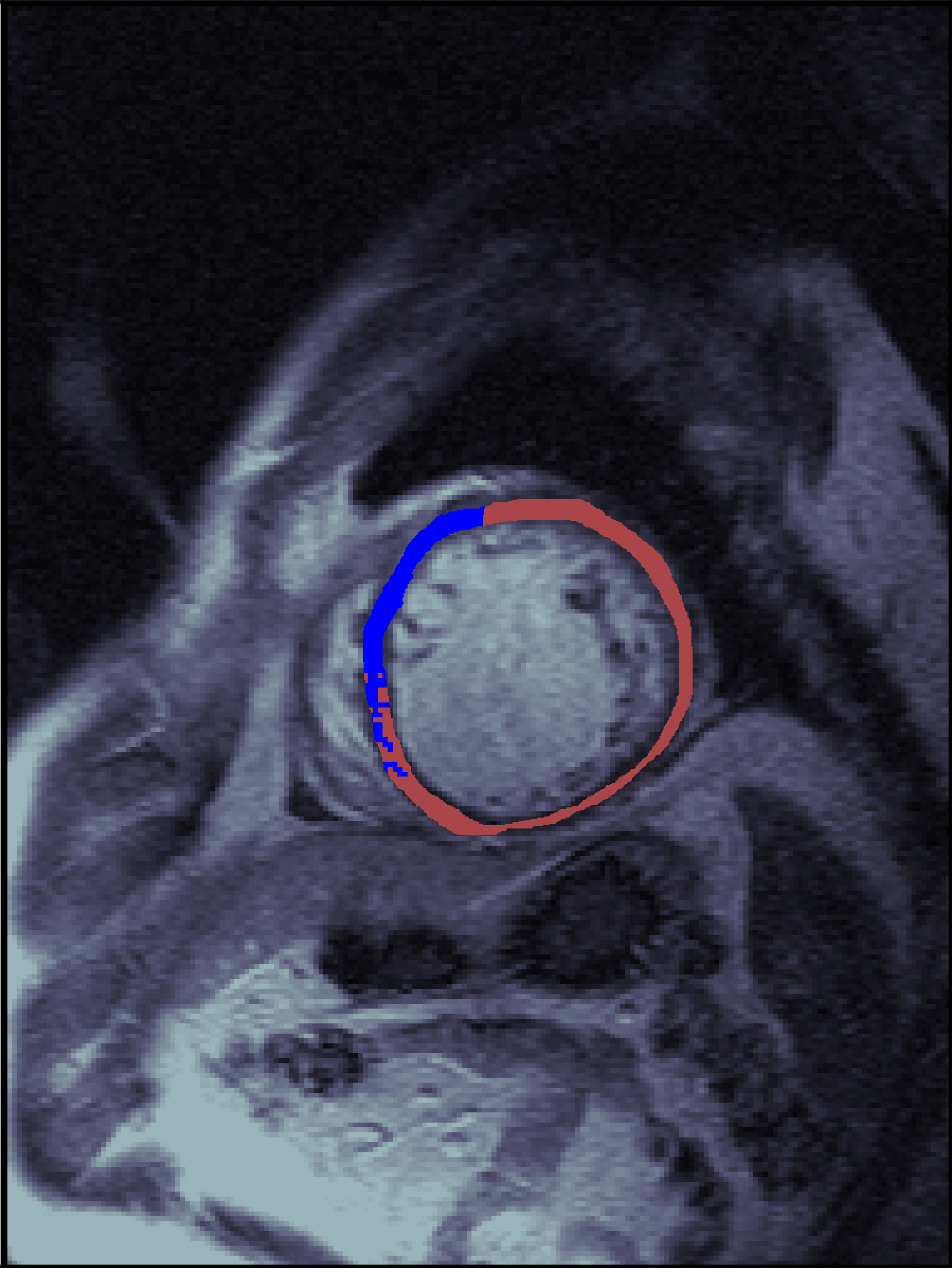

Supplement: S1 Dataset — (ZIP) [file pcbi.1007421.s001.zip › supplementary_segmented_lgemri_data/segmentations/07_18651/-124_ROW_20070822084820.png]

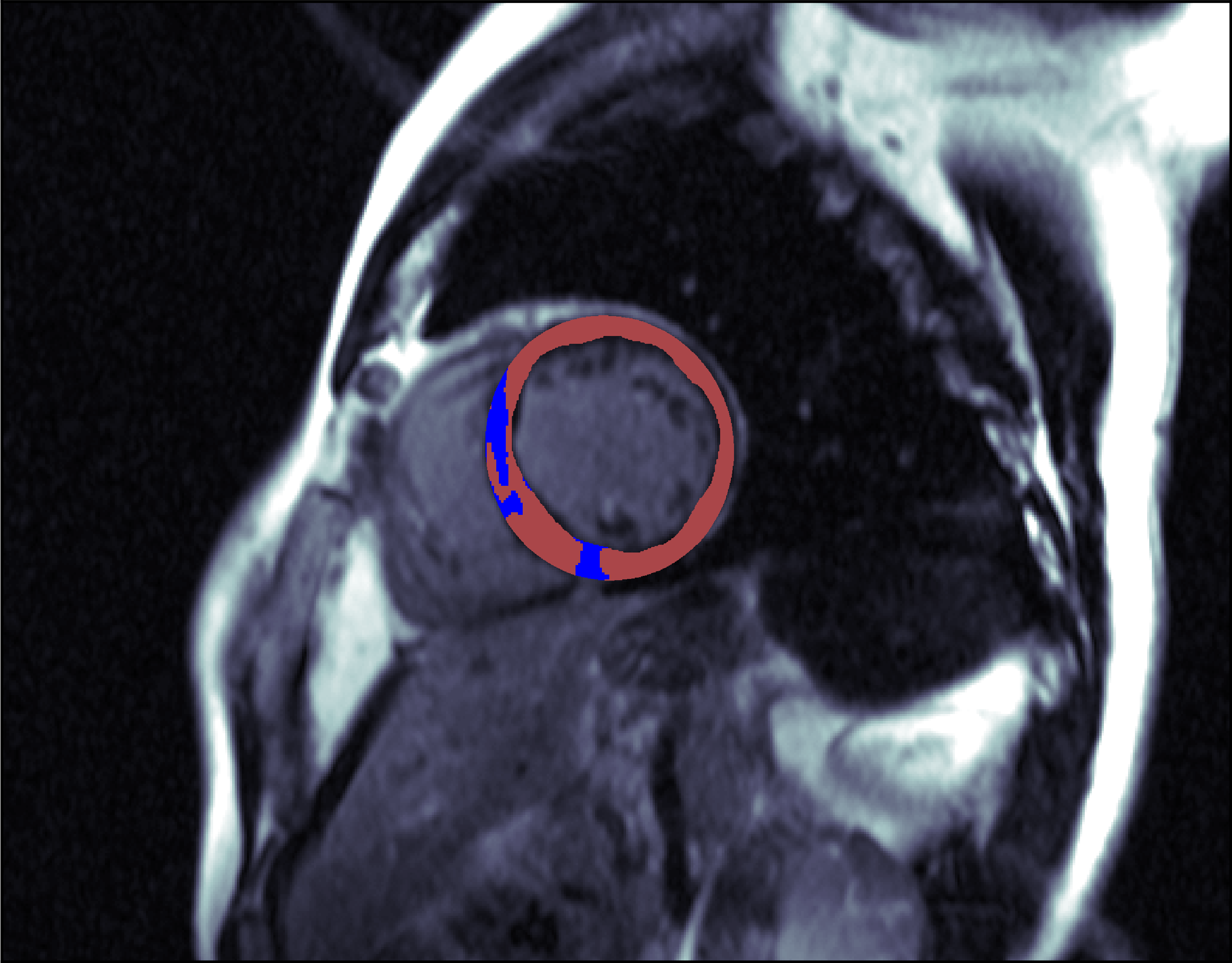

Supplement: S1 Dataset — (ZIP) [file pcbi.1007421.s001.zip › supplementary_segmented_lgemri_data/segmentations/07_18643/70_COL_20071005130111.png]

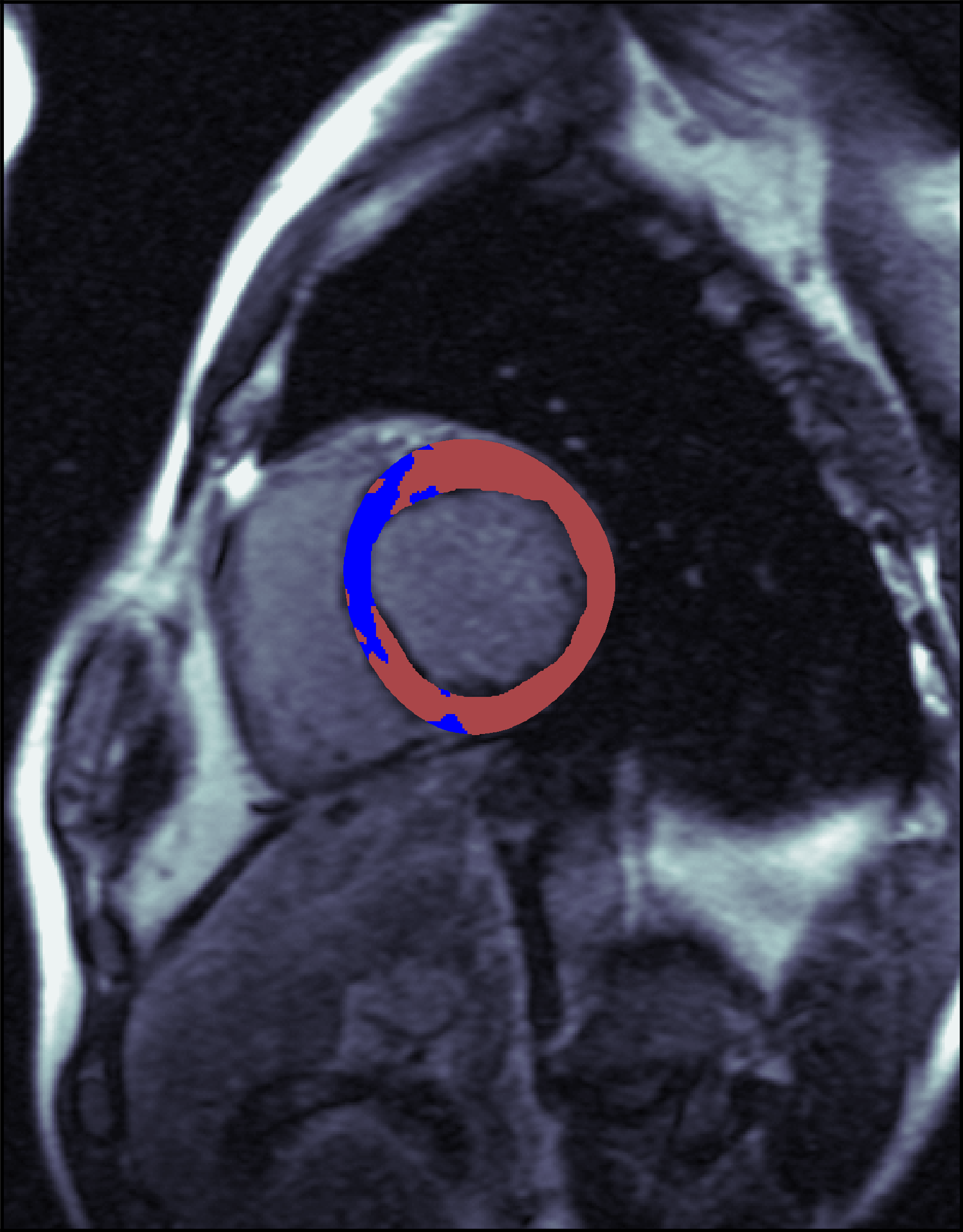

Supplement: S1 Dataset — (ZIP) [file pcbi.1007421.s001.zip › supplementary_segmented_lgemri_data/segmentations/07_18643/59_ROW_20071005125548.png]

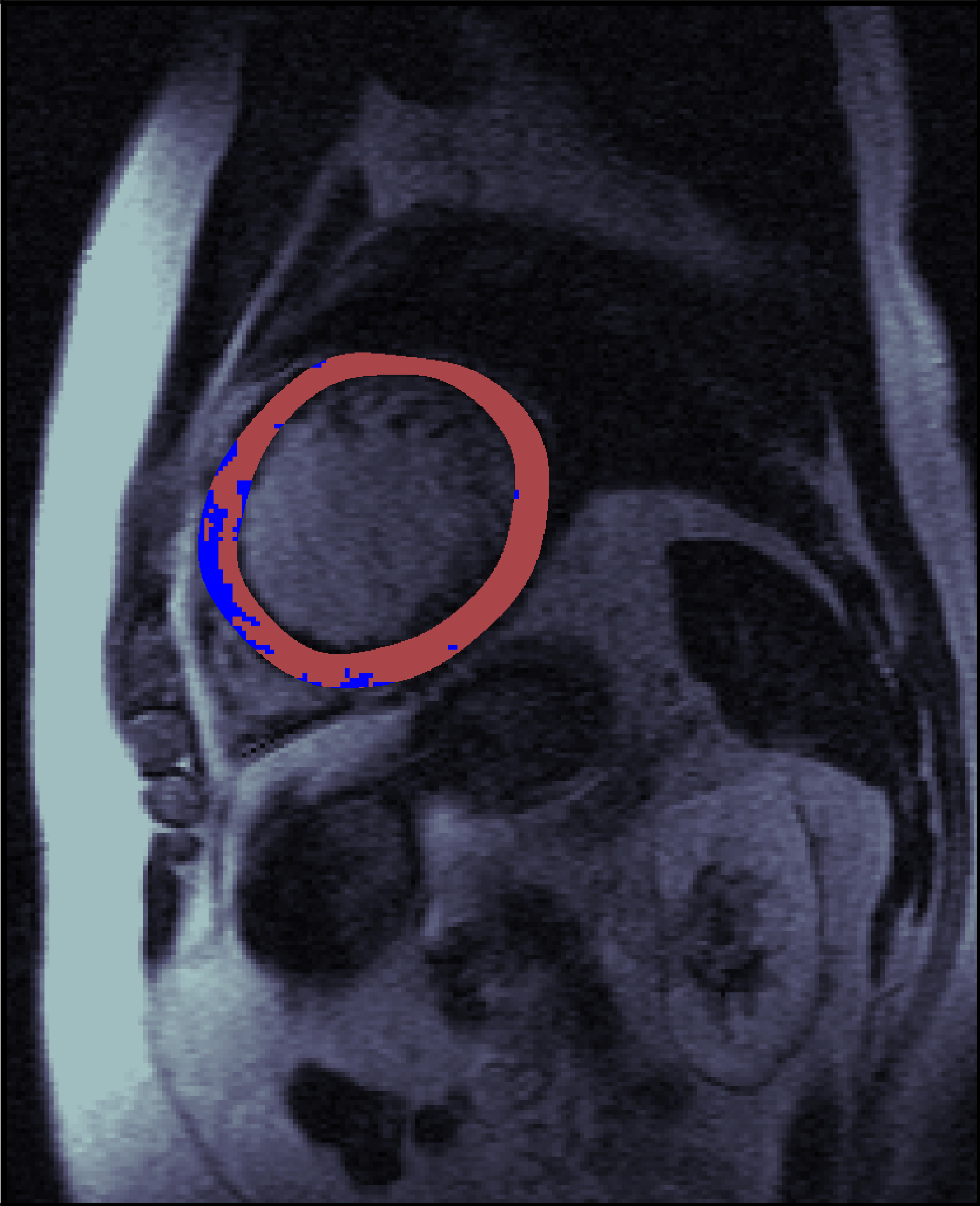

Supplement: S1 Dataset — (ZIP) [file pcbi.1007421.s001.zip › supplementary_segmented_lgemri_data/segmentations/07_14899/95_ROW_20070627154921.png]

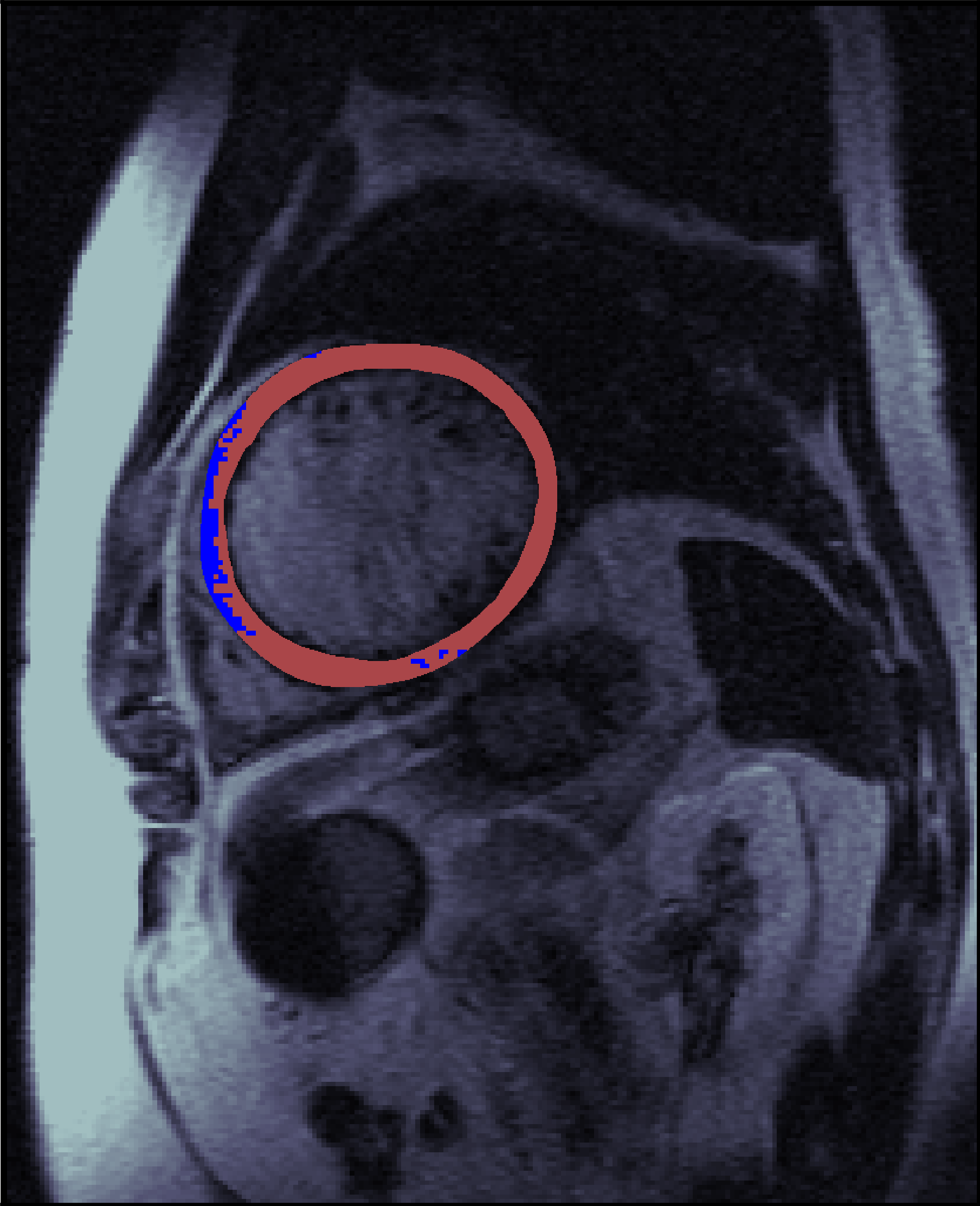

Supplement: S1 Dataset — (ZIP) [file pcbi.1007421.s001.zip › supplementary_segmented_lgemri_data/segmentations/07_14899/85_ROW_20070627154846.png]

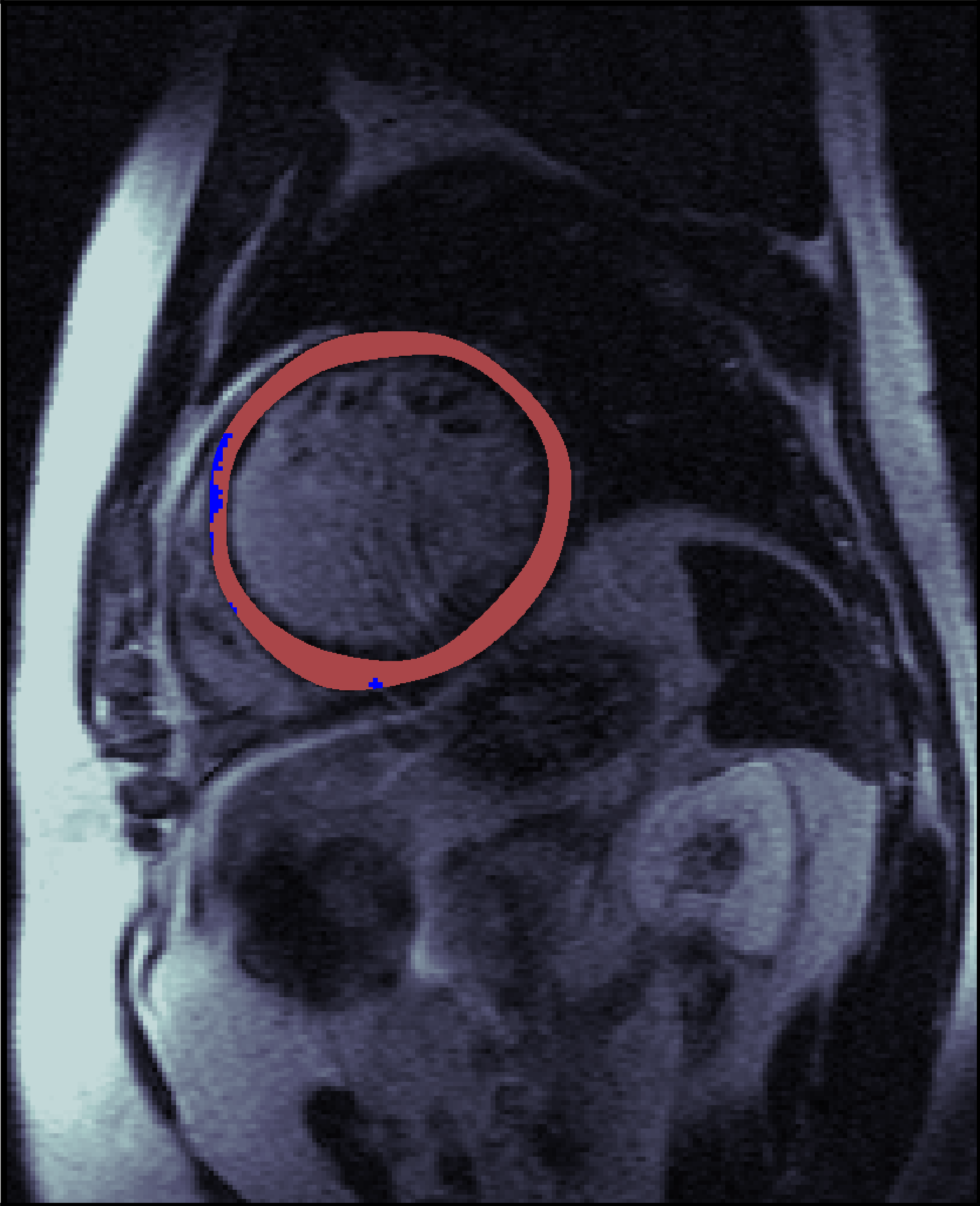

Supplement: S1 Dataset — (ZIP) [file pcbi.1007421.s001.zip › supplementary_segmented_lgemri_data/segmentations/07_14899/75_ROW_20070627154815.png]

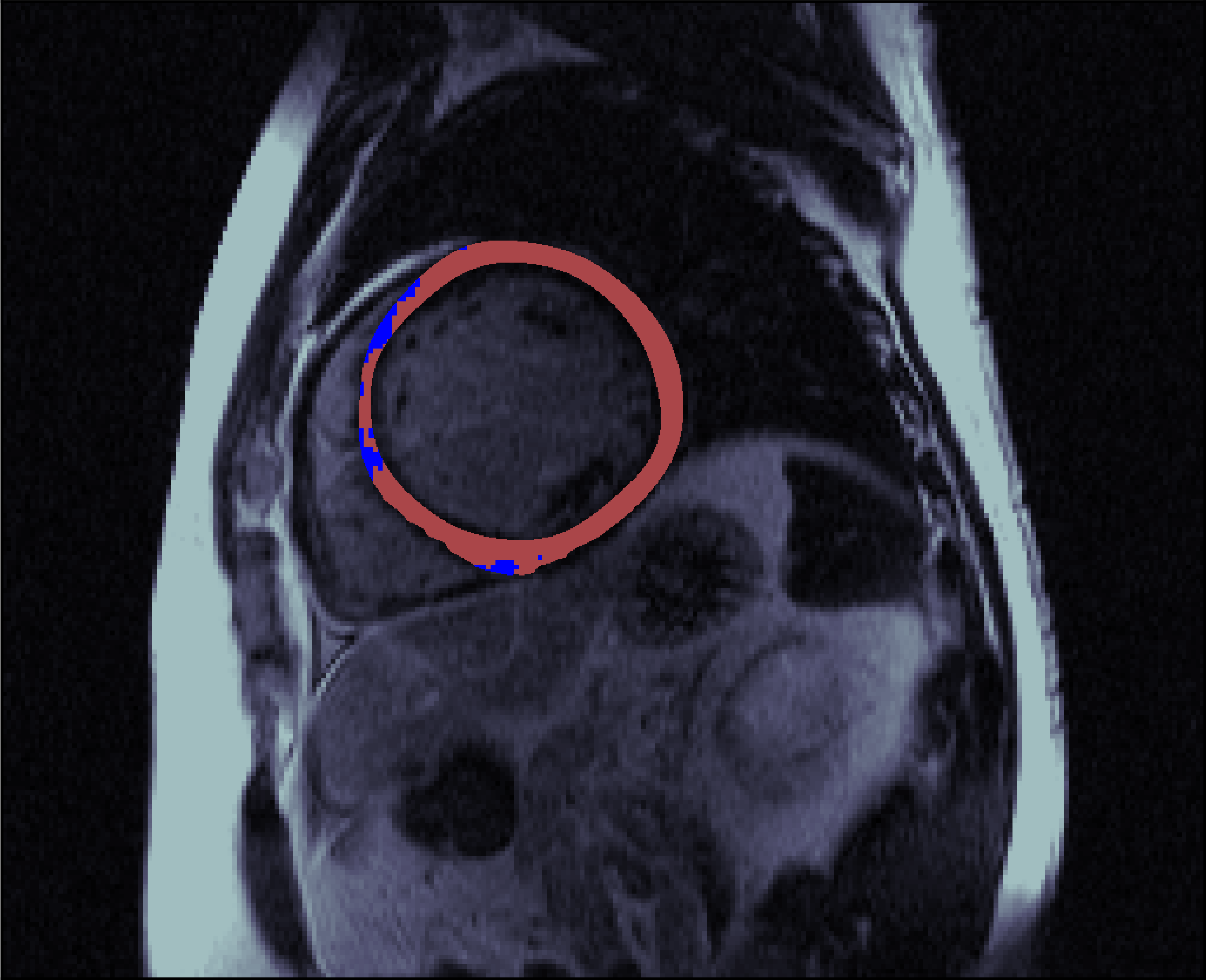

Supplement: S1 Dataset — (ZIP) [file pcbi.1007421.s001.zip › supplementary_segmented_lgemri_data/segmentations/07_14899/55_COL_20070627155347.png]

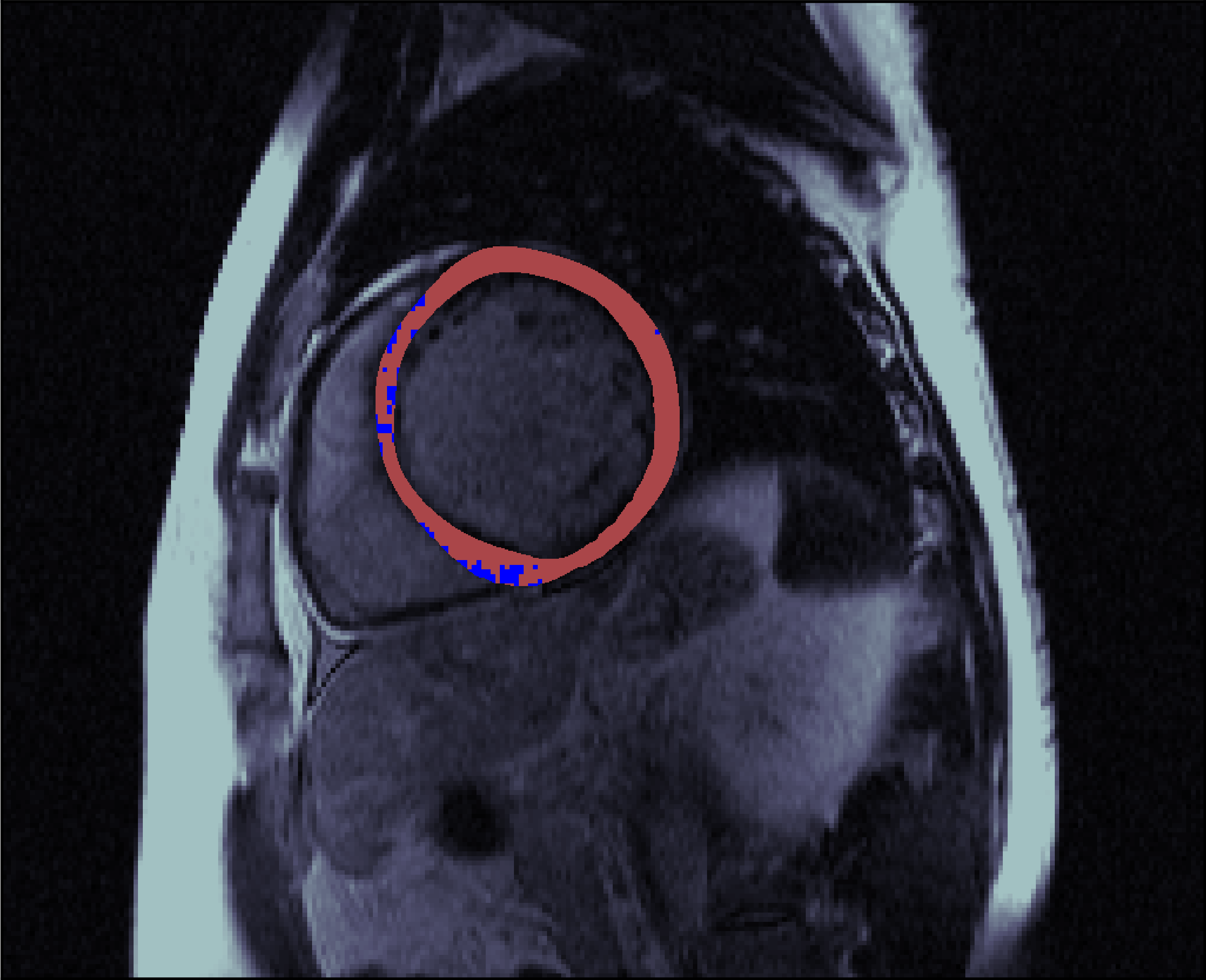

Supplement: S1 Dataset — (ZIP) [file pcbi.1007421.s001.zip › supplementary_segmented_lgemri_data/segmentations/07_14899/45_COL_20070627155251.png]

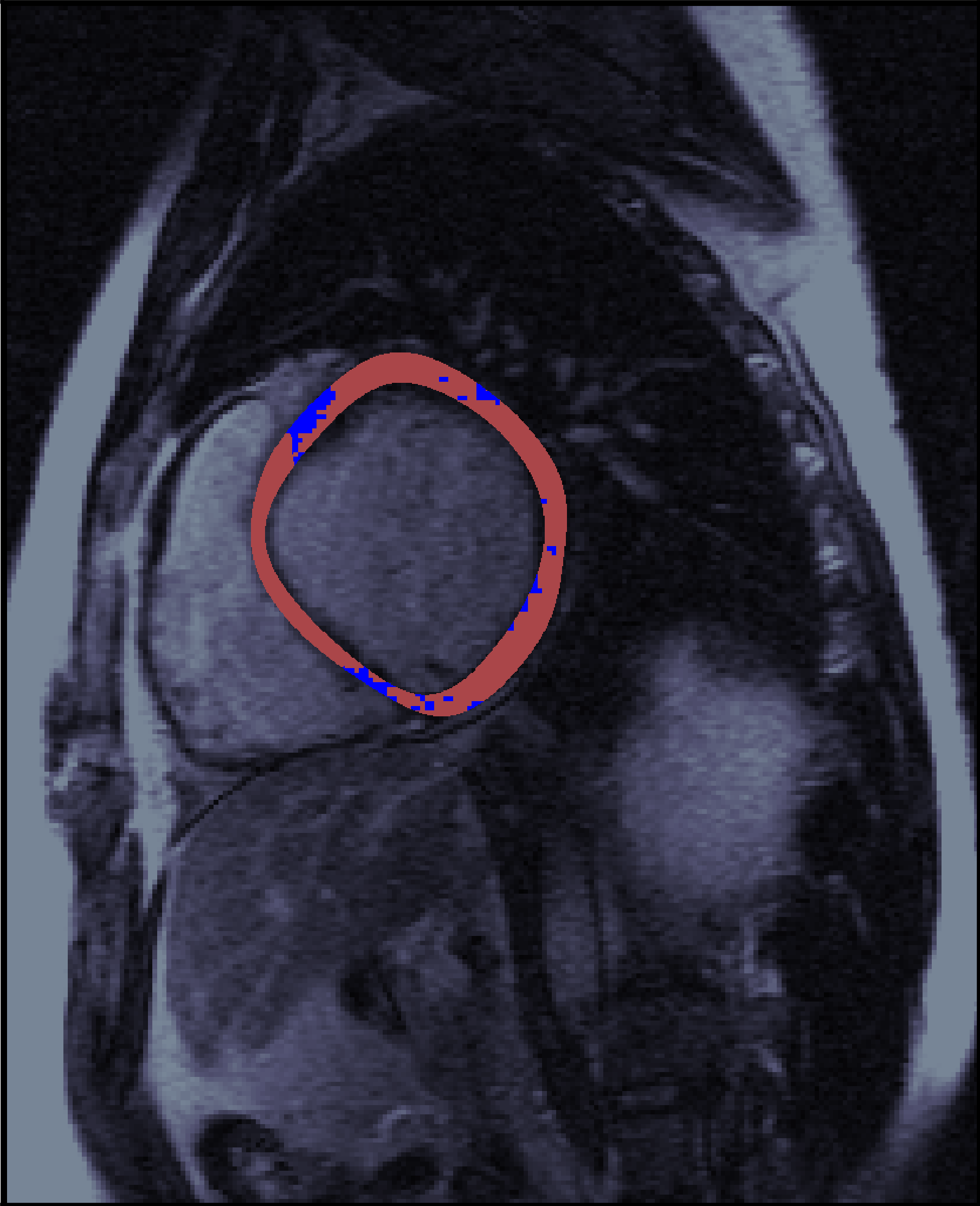

Supplement: S1 Dataset — (ZIP) [file pcbi.1007421.s001.zip › supplementary_segmented_lgemri_data/segmentations/07_14899/35_ROW_20070627154437.png]

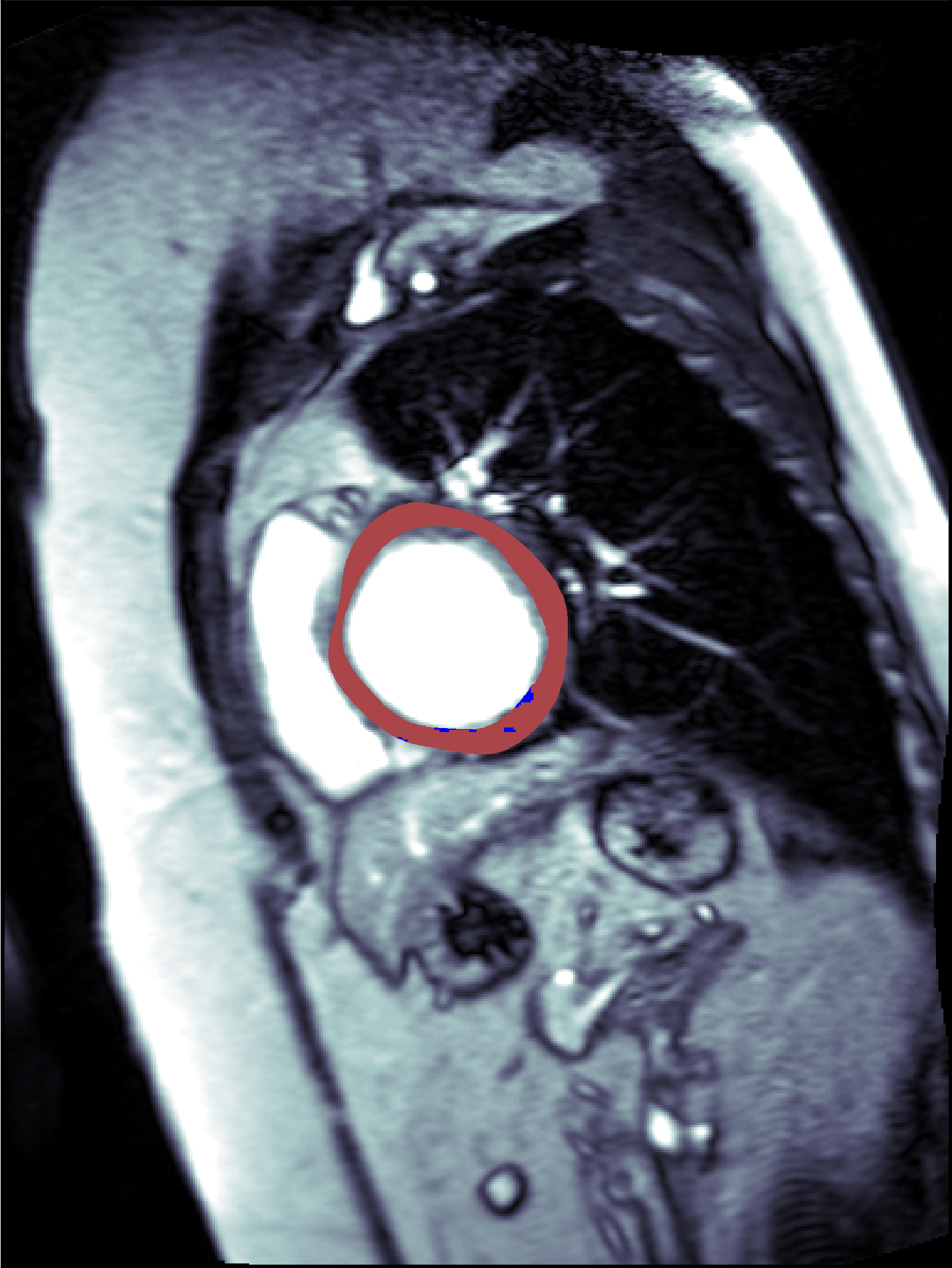

Supplement: S1 Dataset — (ZIP) [file pcbi.1007421.s001.zip › supplementary_segmented_lgemri_data/segmentations/07_14321/30_ROW_20121211122344.png]

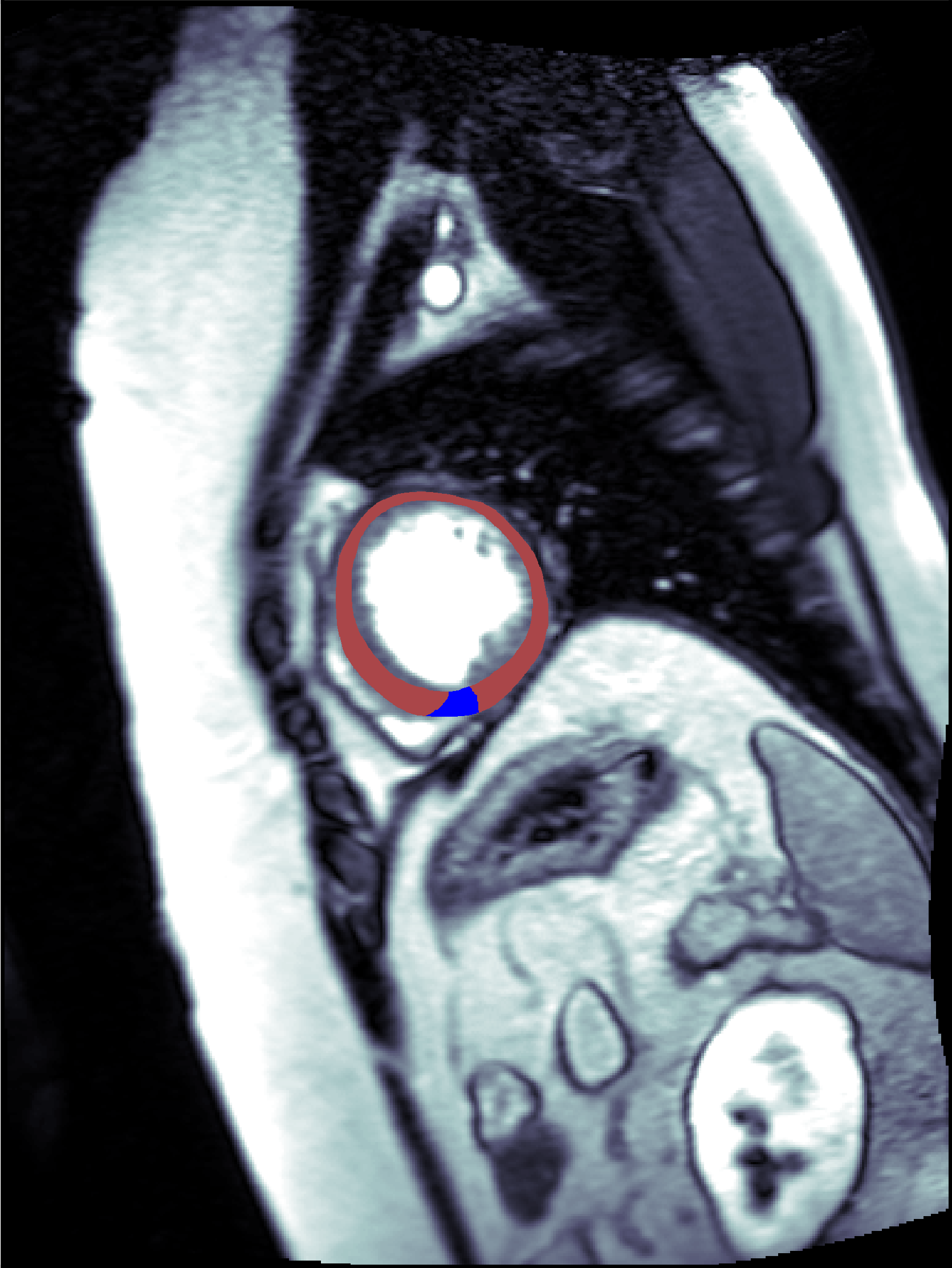

Supplement: S1 Dataset — (ZIP) [file pcbi.1007421.s001.zip › supplementary_segmented_lgemri_data/segmentations/07_14321/80_ROW_20121211122552.png]

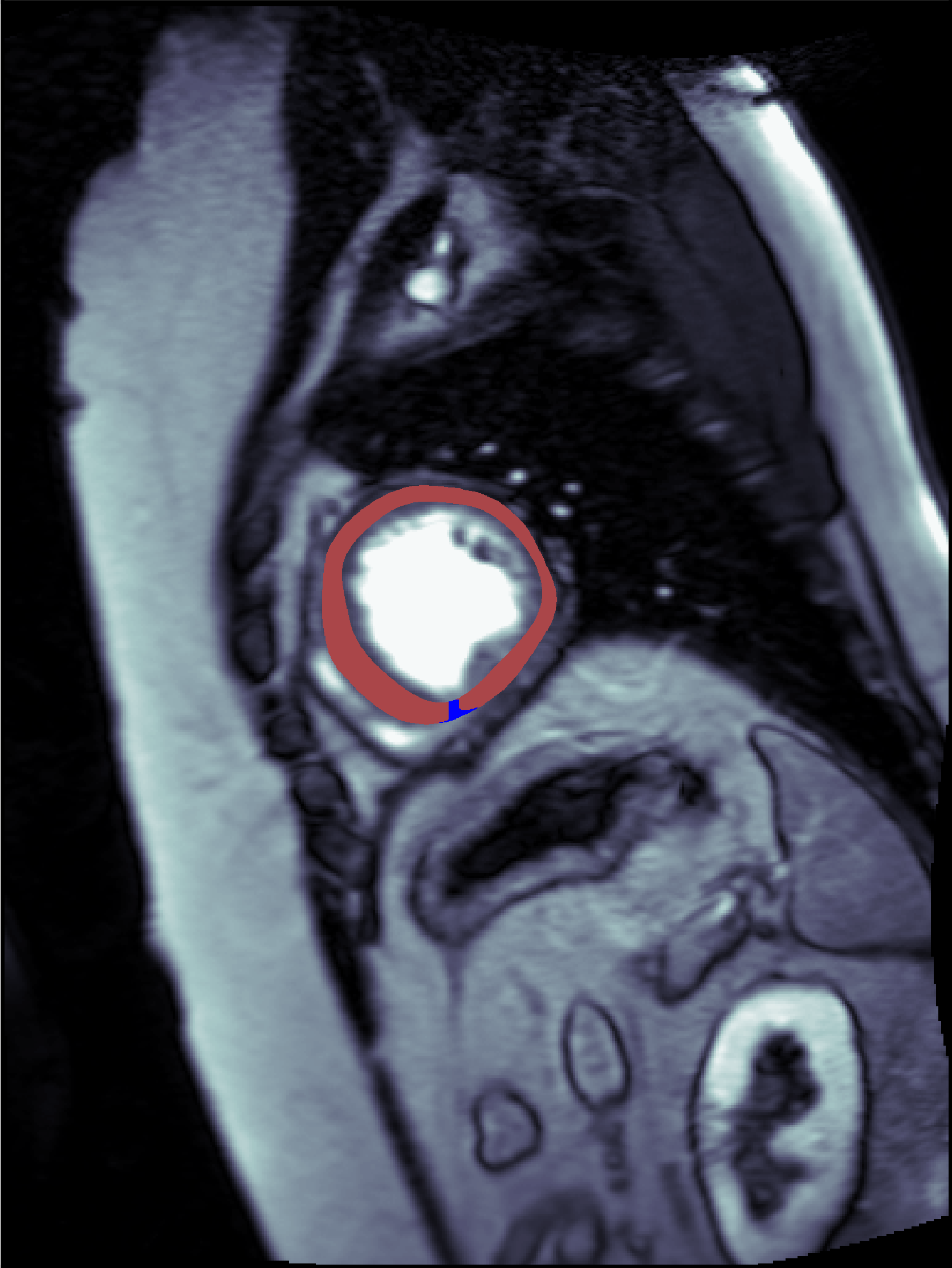

Supplement: S1 Dataset — (ZIP) [file pcbi.1007421.s001.zip › supplementary_segmented_lgemri_data/segmentations/07_14321/70_ROW_20121211122531.png]

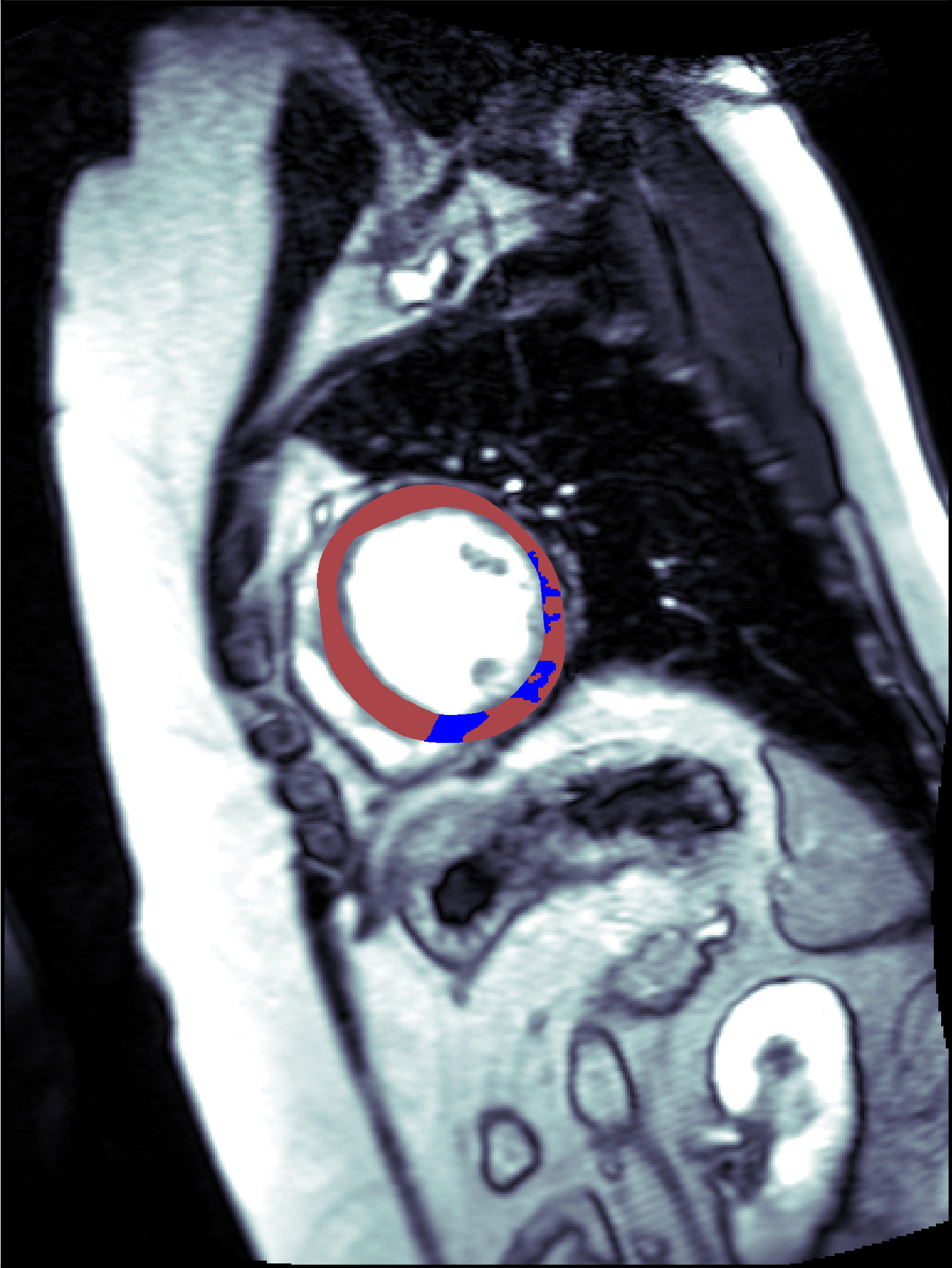

Supplement: S1 Dataset — (ZIP) [file pcbi.1007421.s001.zip › supplementary_segmented_lgemri_data/segmentations/07_14321/60_ROW_20121211122512.png]

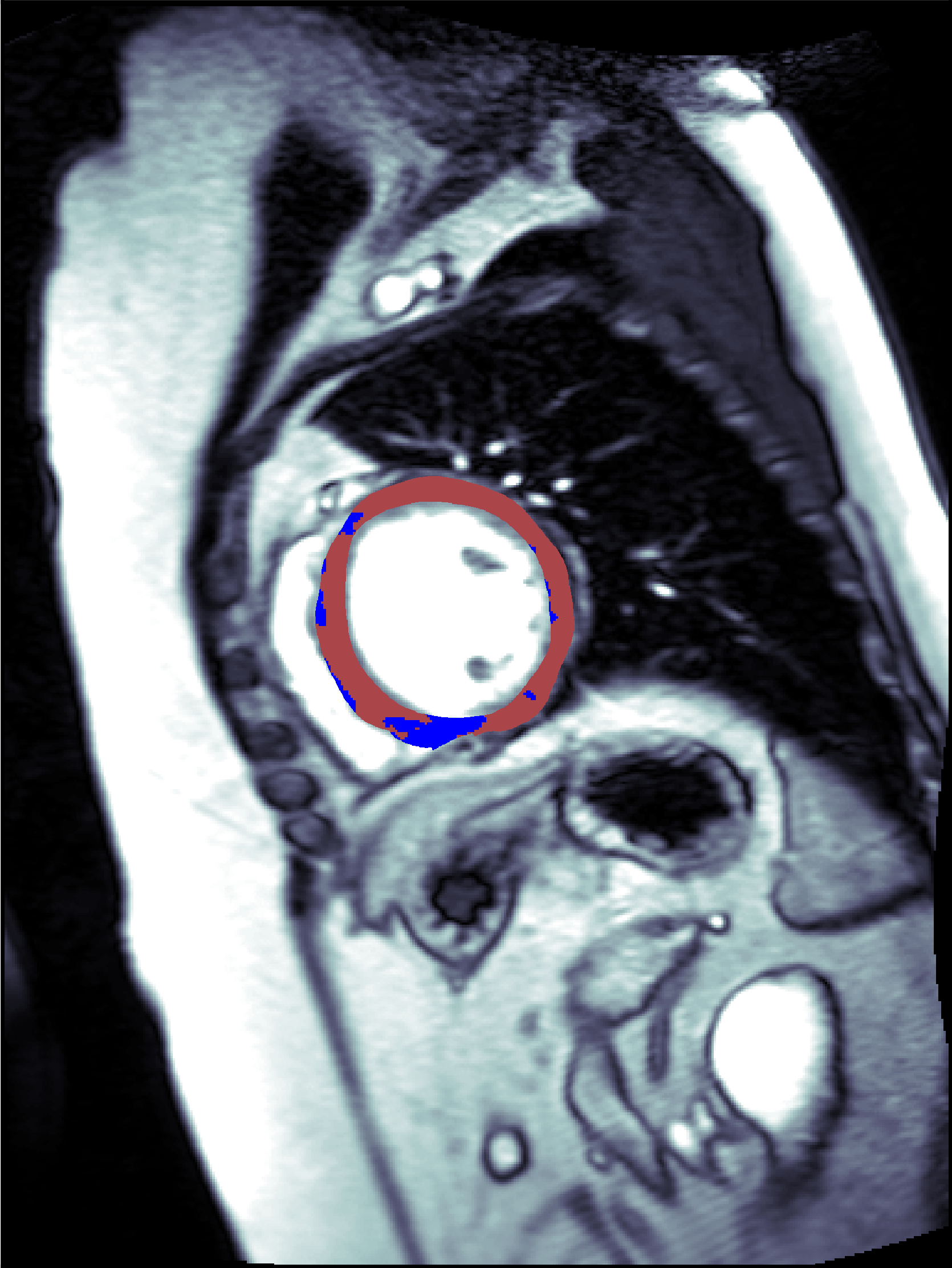

Supplement: S1 Dataset — (ZIP) [file pcbi.1007421.s001.zip › supplementary_segmented_lgemri_data/segmentations/07_14321/50_ROW_20121211122454.png]

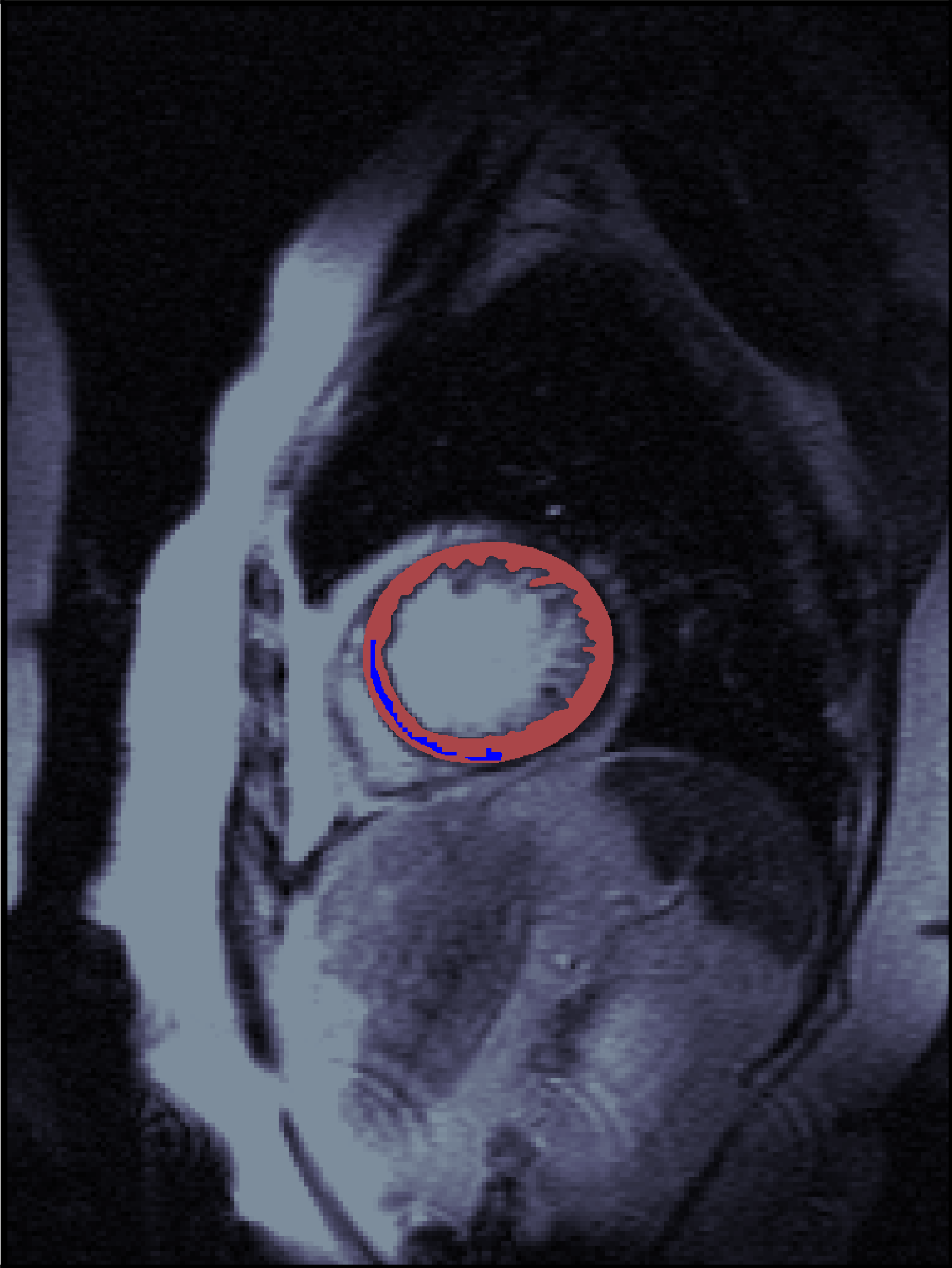

Supplement: S1 Dataset — (ZIP) [file pcbi.1007421.s001.zip › supplementary_segmented_lgemri_data/segmentations/07_10248/109_ROW_20070605125451.png]

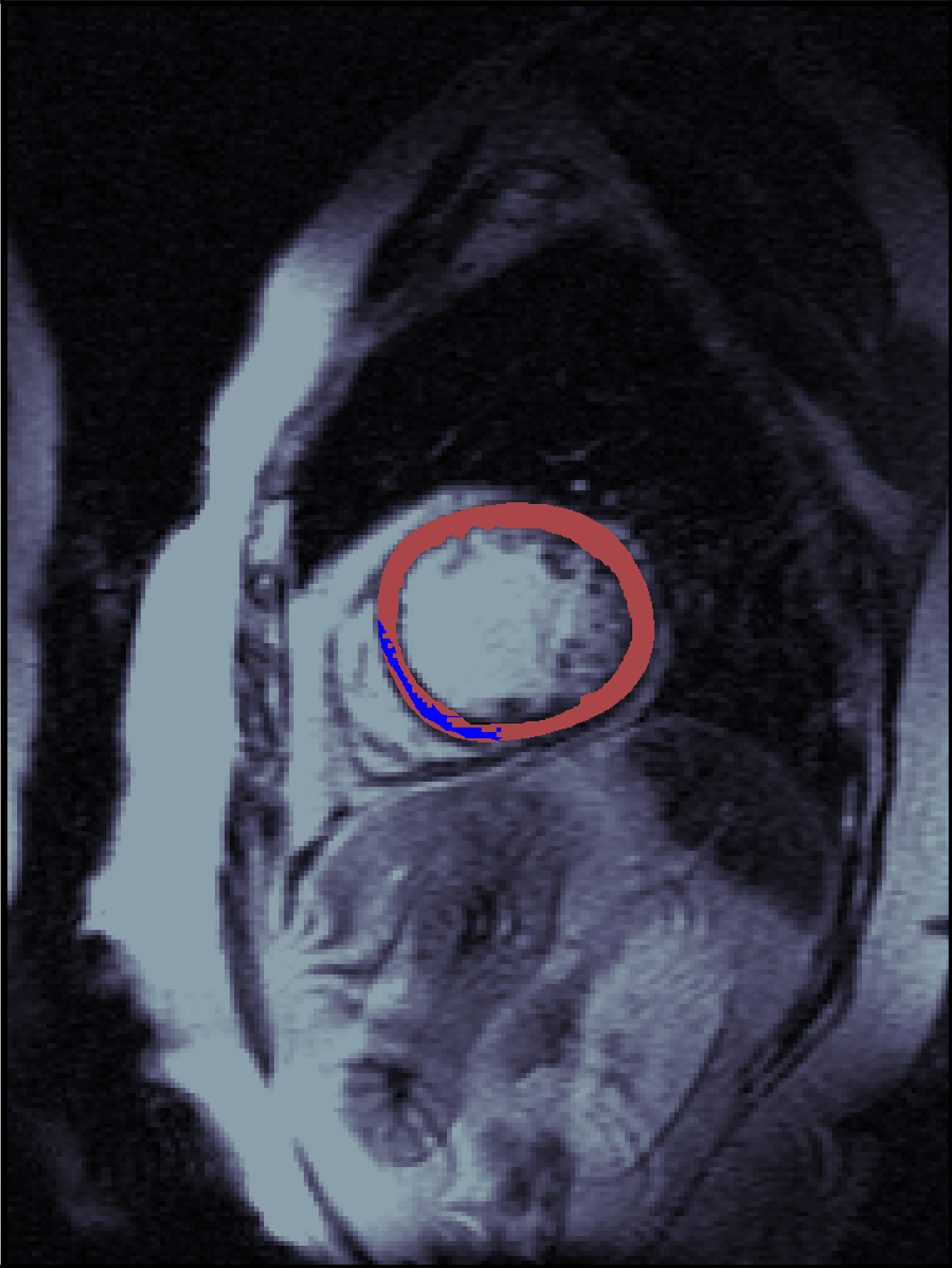

Supplement: S1 Dataset — (ZIP) [file pcbi.1007421.s001.zip › supplementary_segmented_lgemri_data/segmentations/07_10248/100_ROW_20070605125426.png]

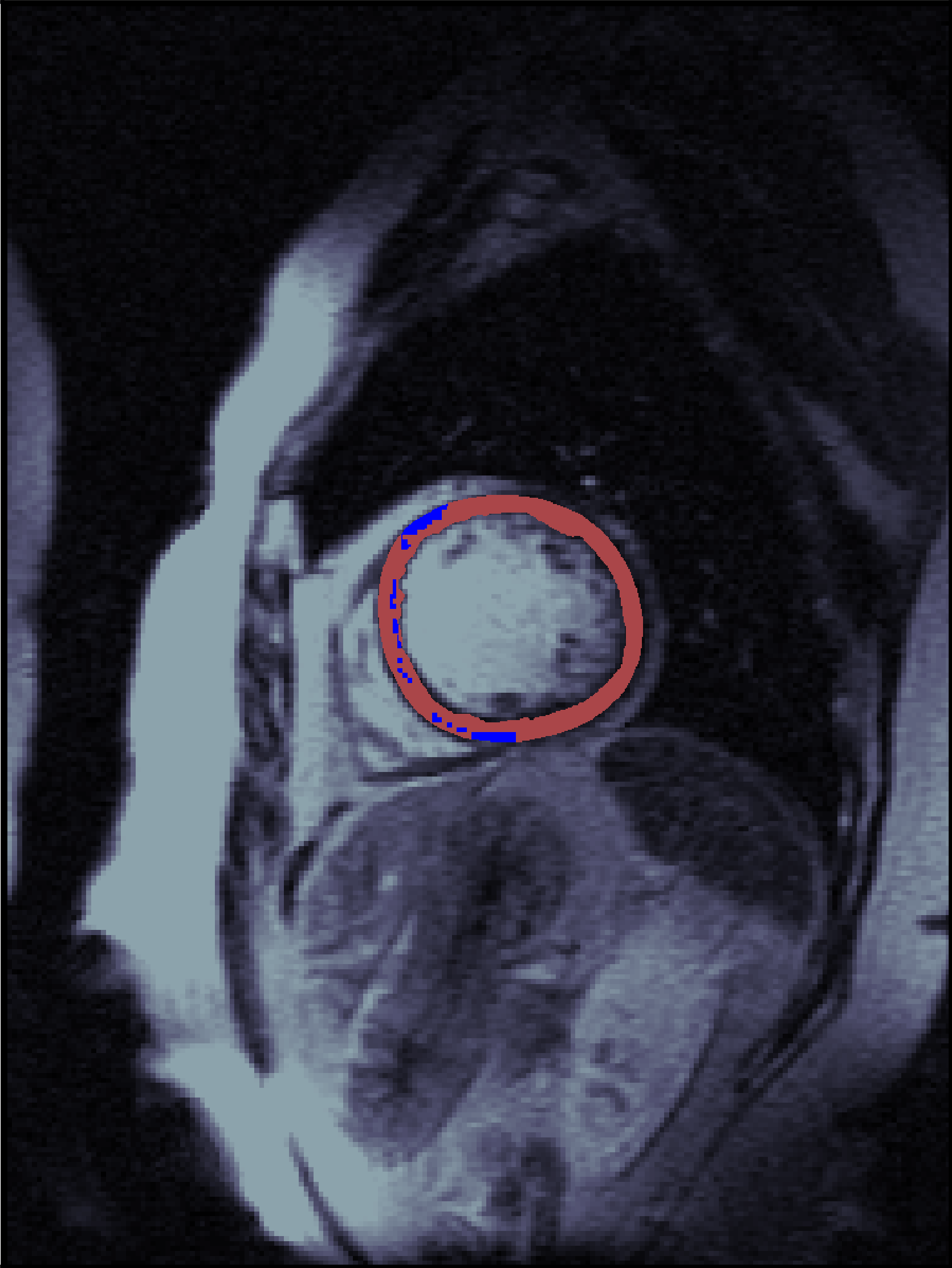

Supplement: S1 Dataset — (ZIP) [file pcbi.1007421.s001.zip › supplementary_segmented_lgemri_data/segmentations/07_10248/100_ROW_20070605125359.png]

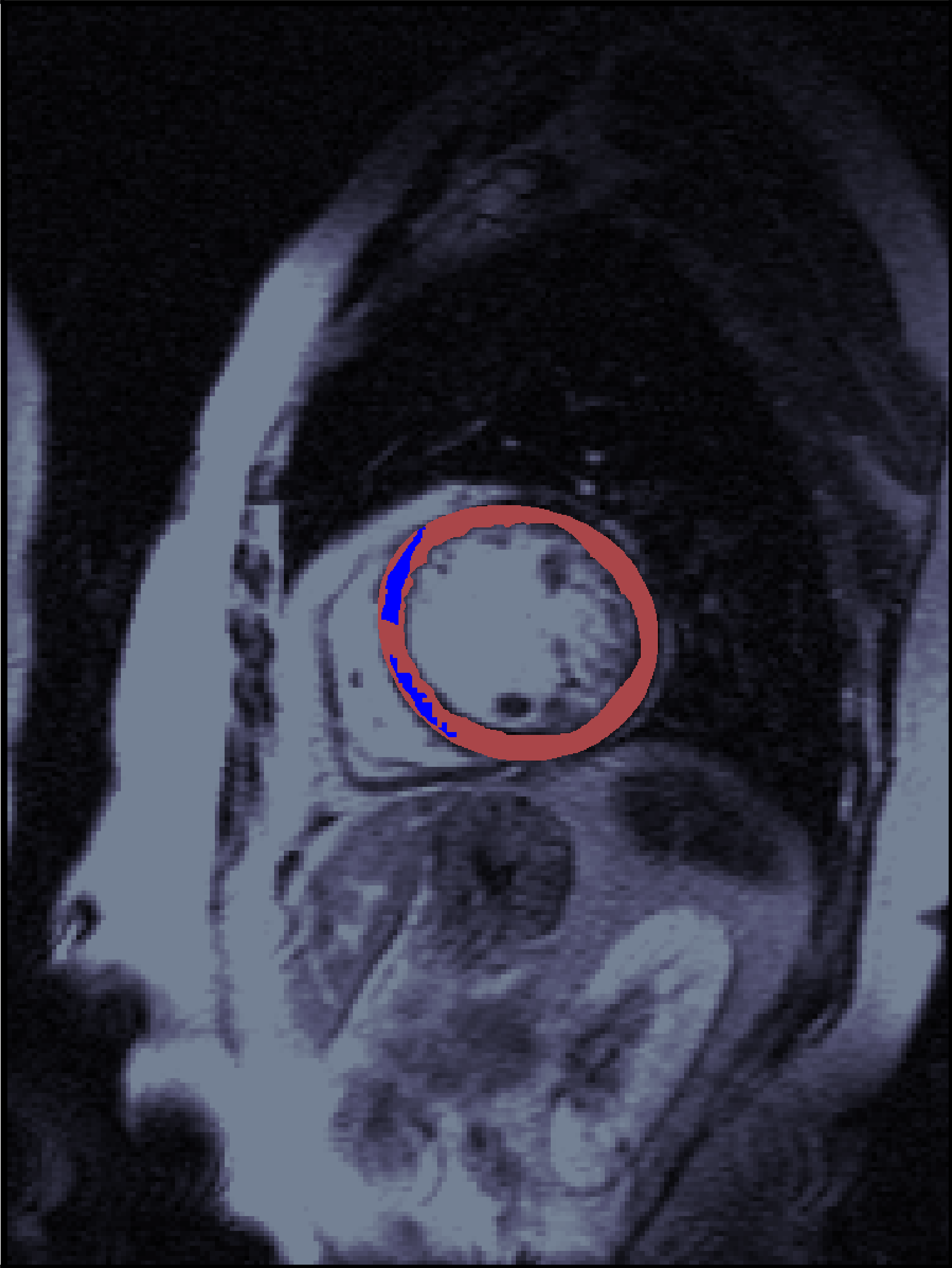

Supplement: S1 Dataset — (ZIP) [file pcbi.1007421.s001.zip › supplementary_segmented_lgemri_data/segmentations/07_10248/90_ROW_20070605125335.png]

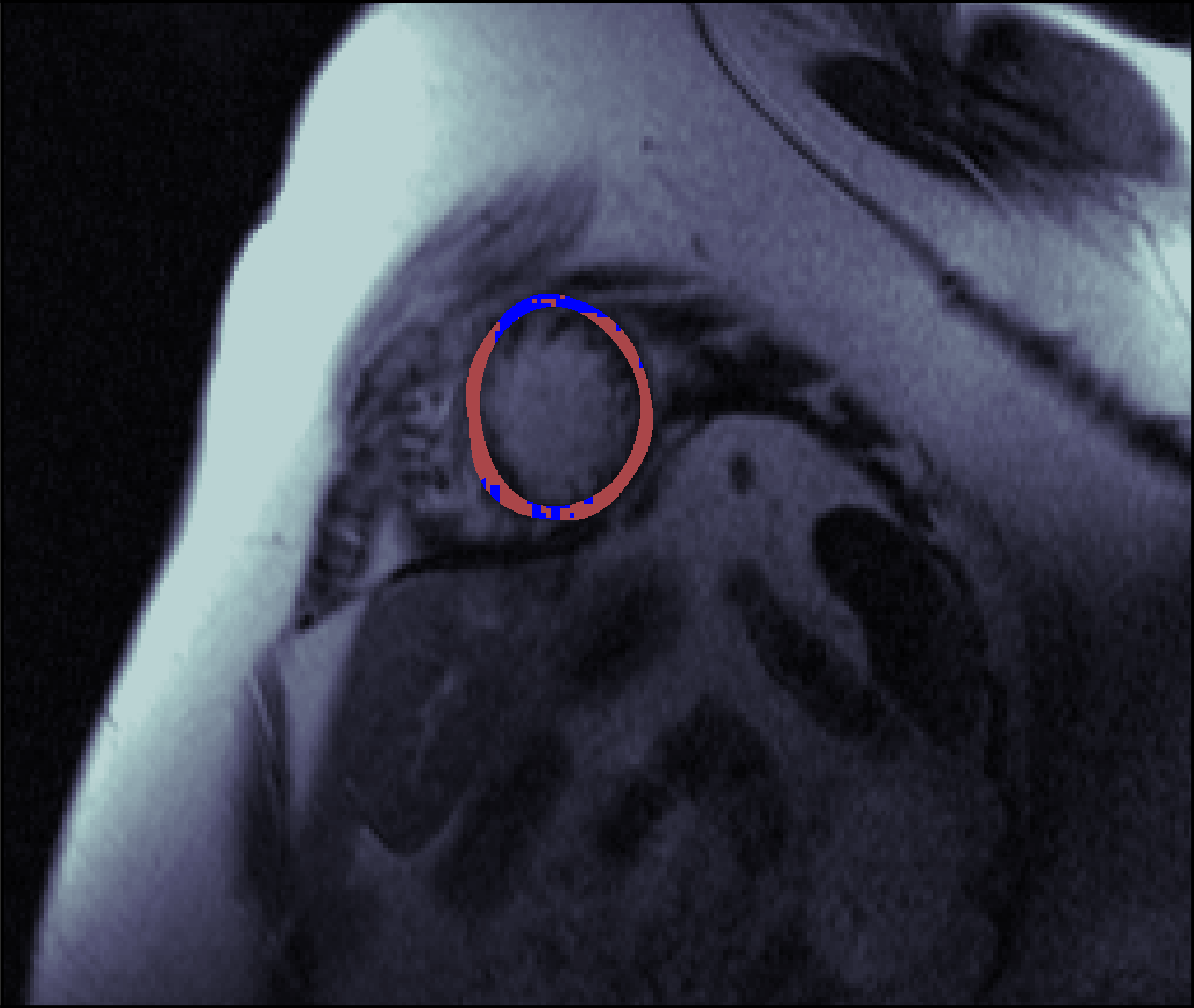

Supplement: S1 Dataset — (ZIP) [file pcbi.1007421.s001.zip › supplementary_segmented_lgemri_data/segmentations/07_04952/126_COL_20070515111212.png]

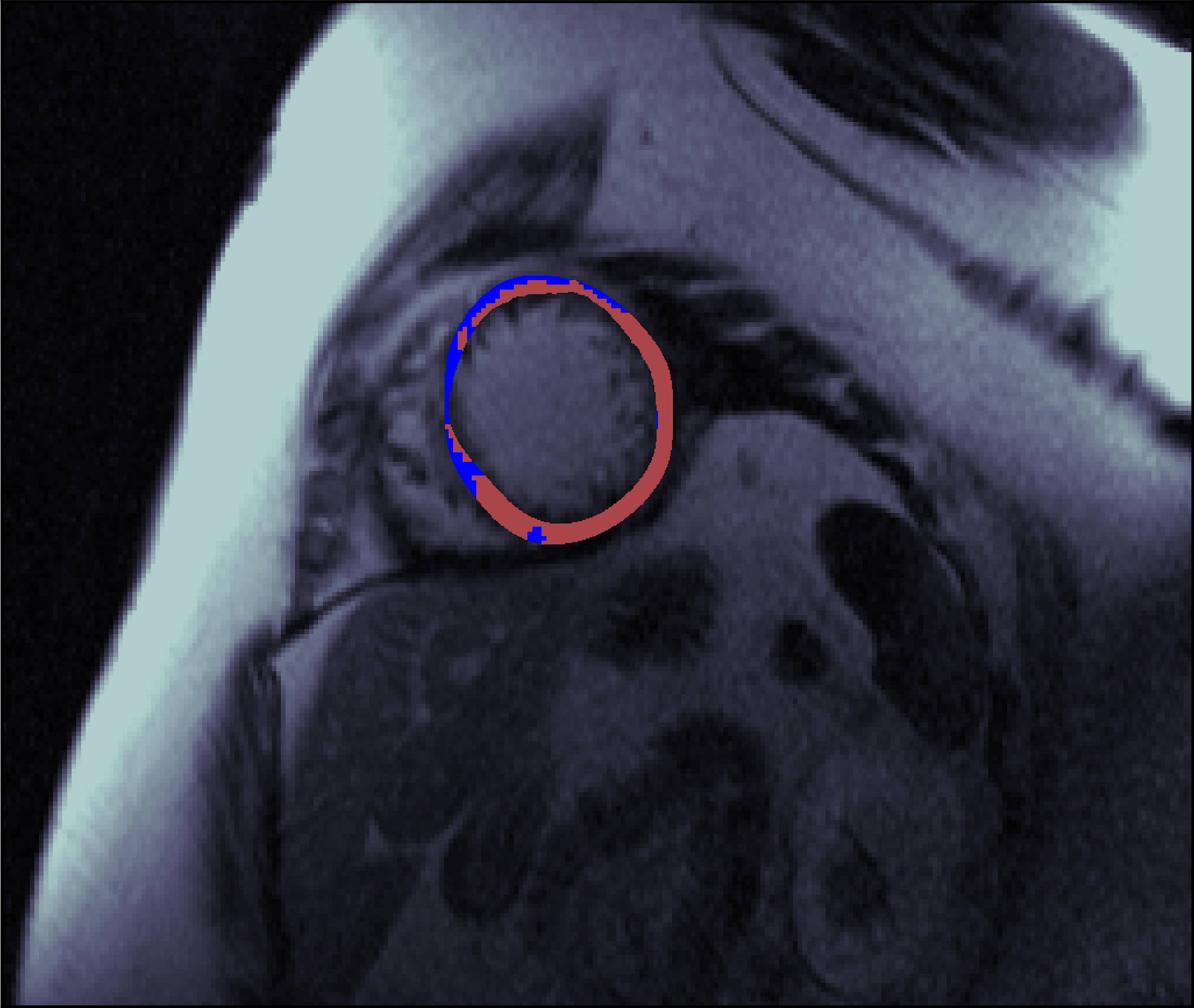

Supplement: S1 Dataset — (ZIP) [file pcbi.1007421.s001.zip › supplementary_segmented_lgemri_data/segmentations/07_04952/116_COL_20070515111120.png]

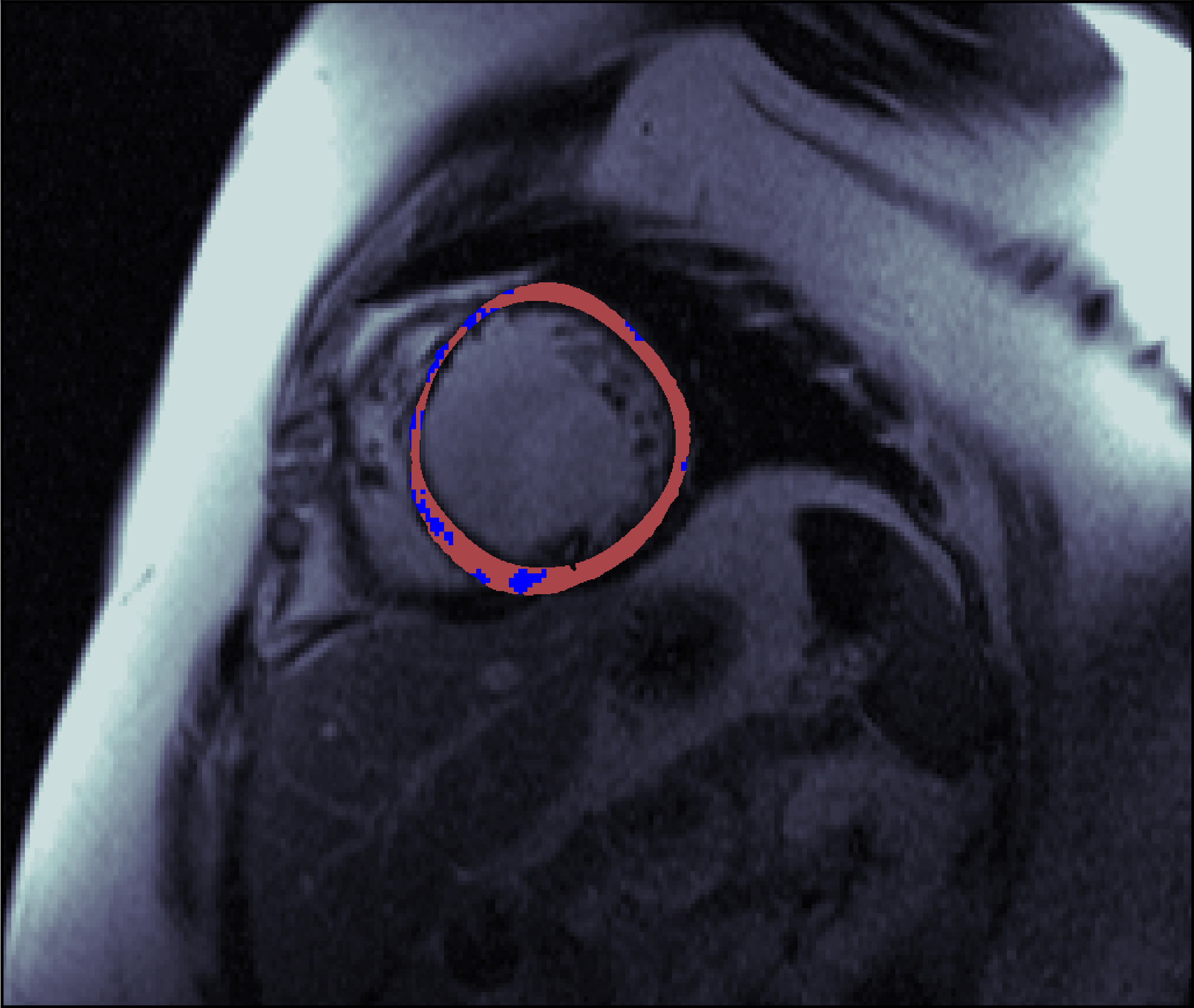

Supplement: S1 Dataset — (ZIP) [file pcbi.1007421.s001.zip › supplementary_segmented_lgemri_data/segmentations/07_04952/106_COL_20070515111044.png]

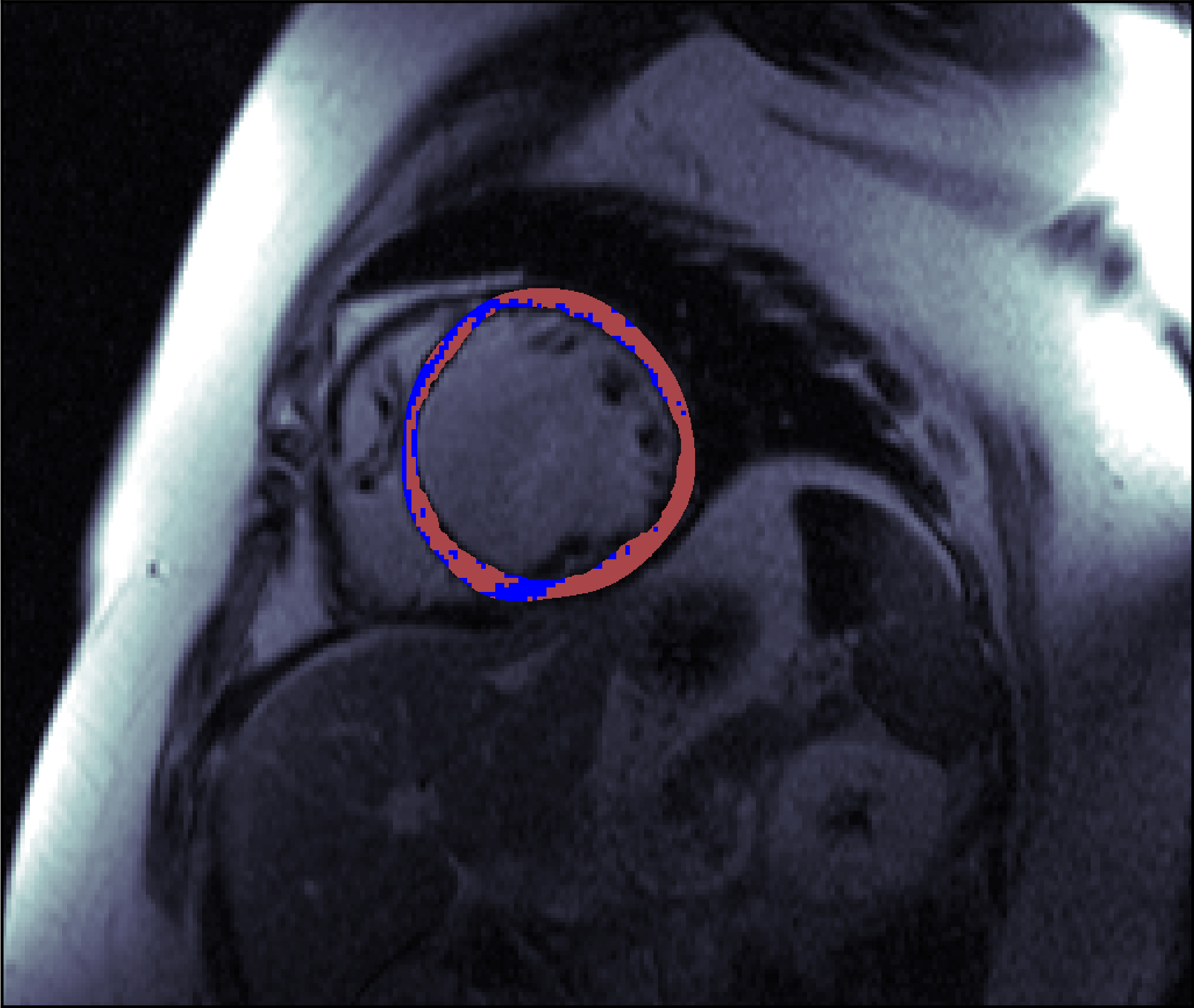

Supplement: S1 Dataset — (ZIP) [file pcbi.1007421.s001.zip › supplementary_segmented_lgemri_data/segmentations/07_04952/96_COL_20070515110959.png]

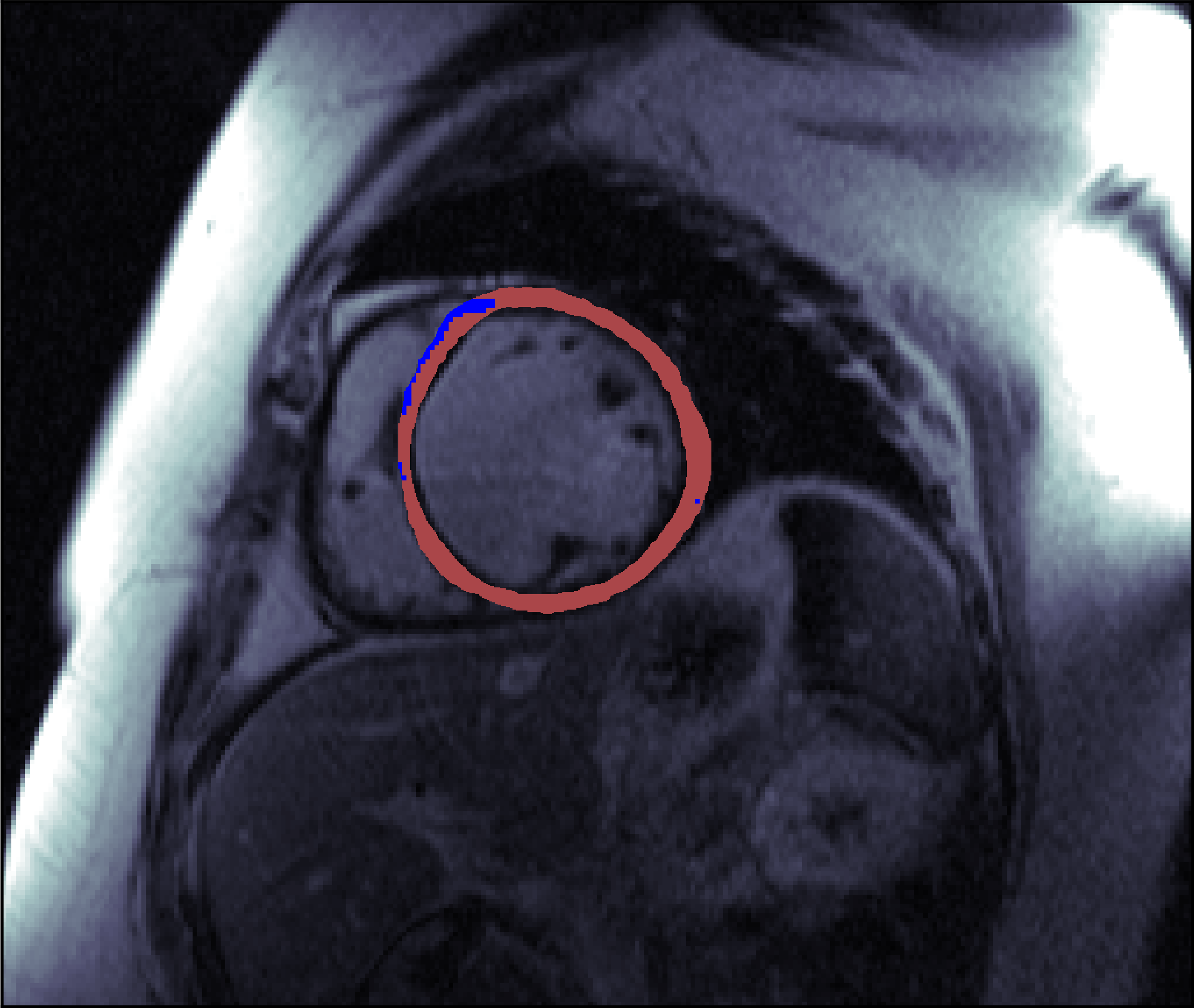

Supplement: S1 Dataset — (ZIP) [file pcbi.1007421.s001.zip › supplementary_segmented_lgemri_data/segmentations/07_04952/87_COL_20070515110910.png]

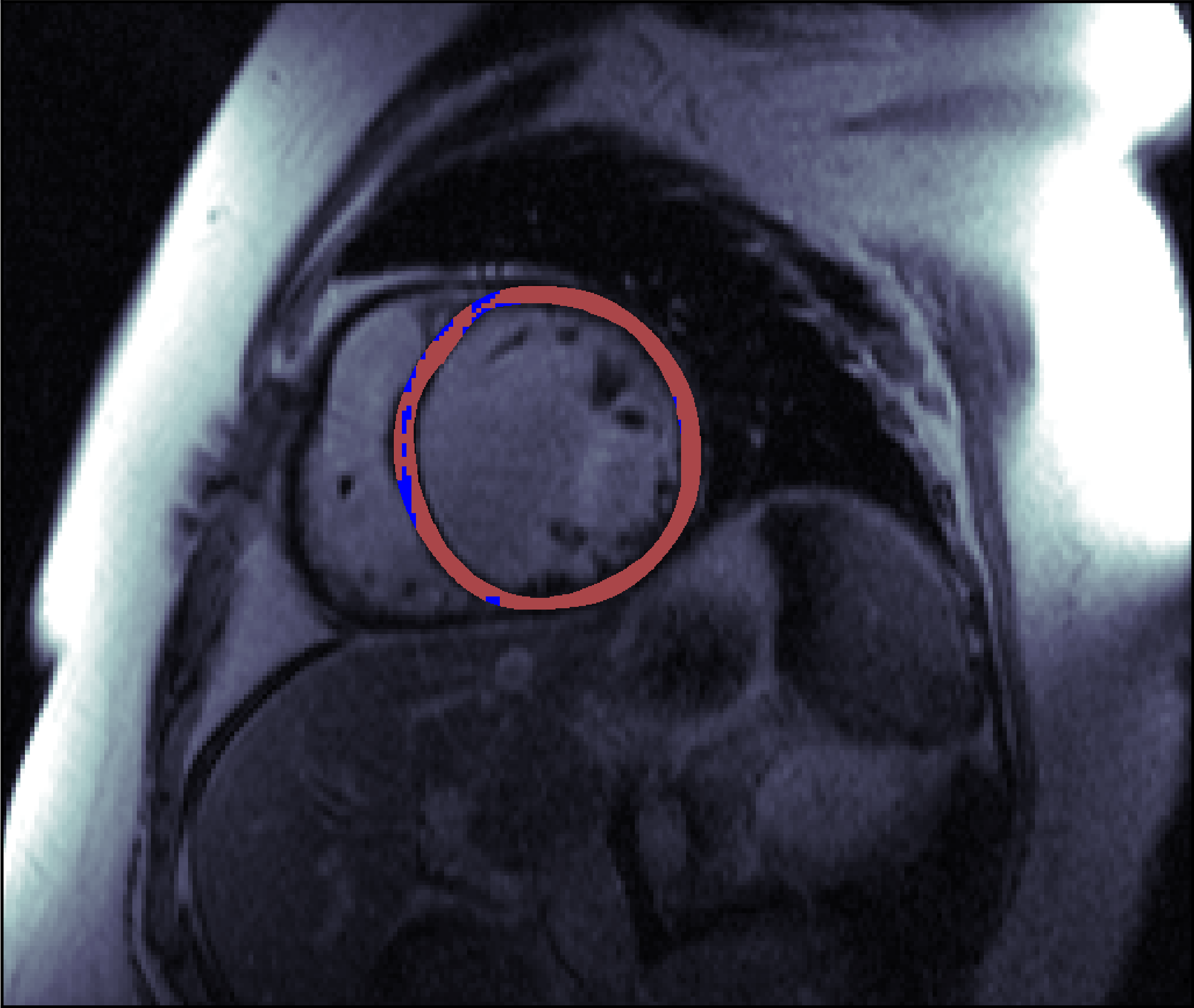

Supplement: S1 Dataset — (ZIP) [file pcbi.1007421.s001.zip › supplementary_segmented_lgemri_data/segmentations/07_04952/76_COL_20070515110821.png]

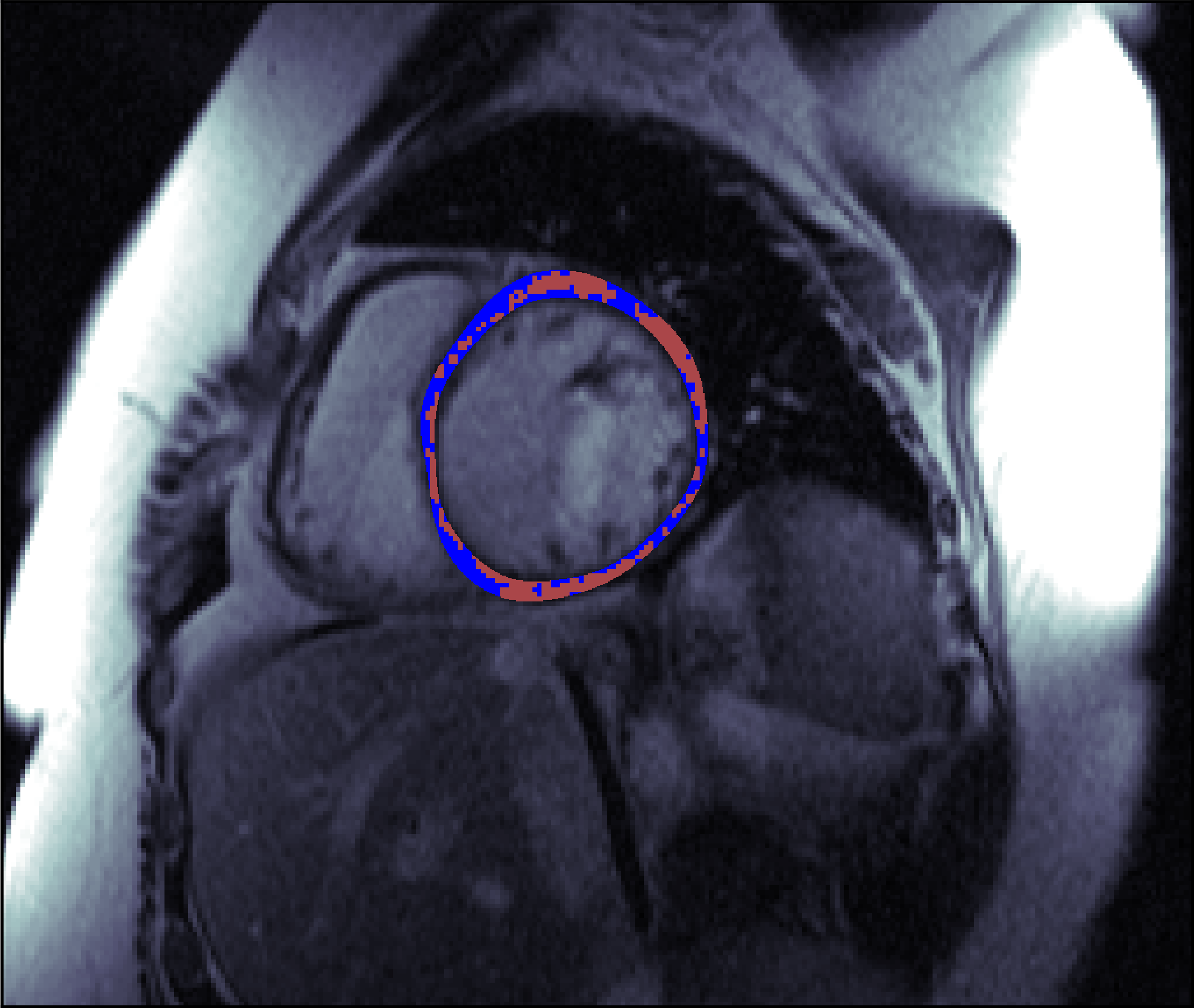

Supplement: S1 Dataset — (ZIP) [file pcbi.1007421.s001.zip › supplementary_segmented_lgemri_data/segmentations/07_04952/56_COL_20070515110706.png]

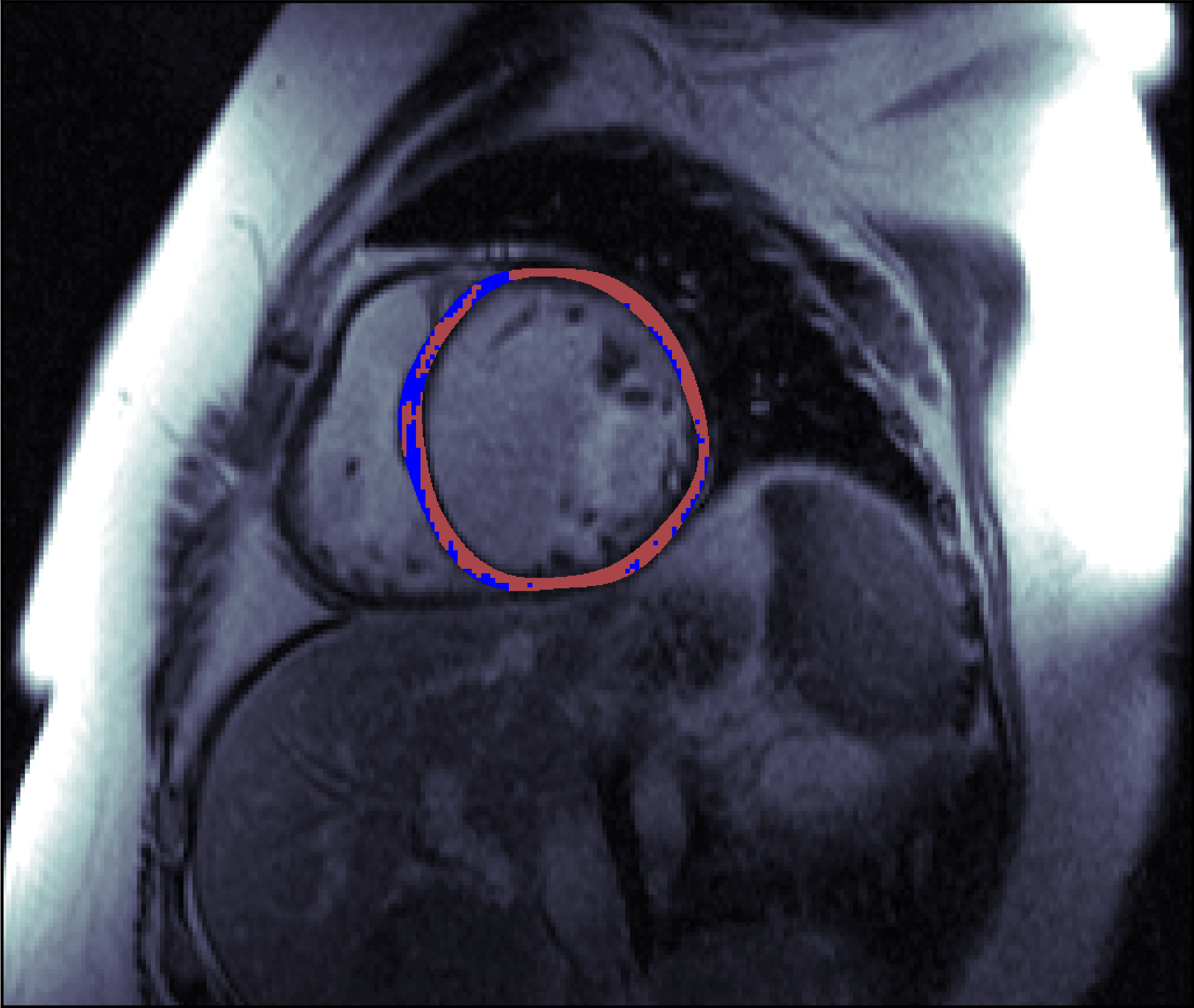

Supplement: S1 Dataset — (ZIP) [file pcbi.1007421.s001.zip › supplementary_segmented_lgemri_data/segmentations/07_04952/66_COL_20070515110747.png]

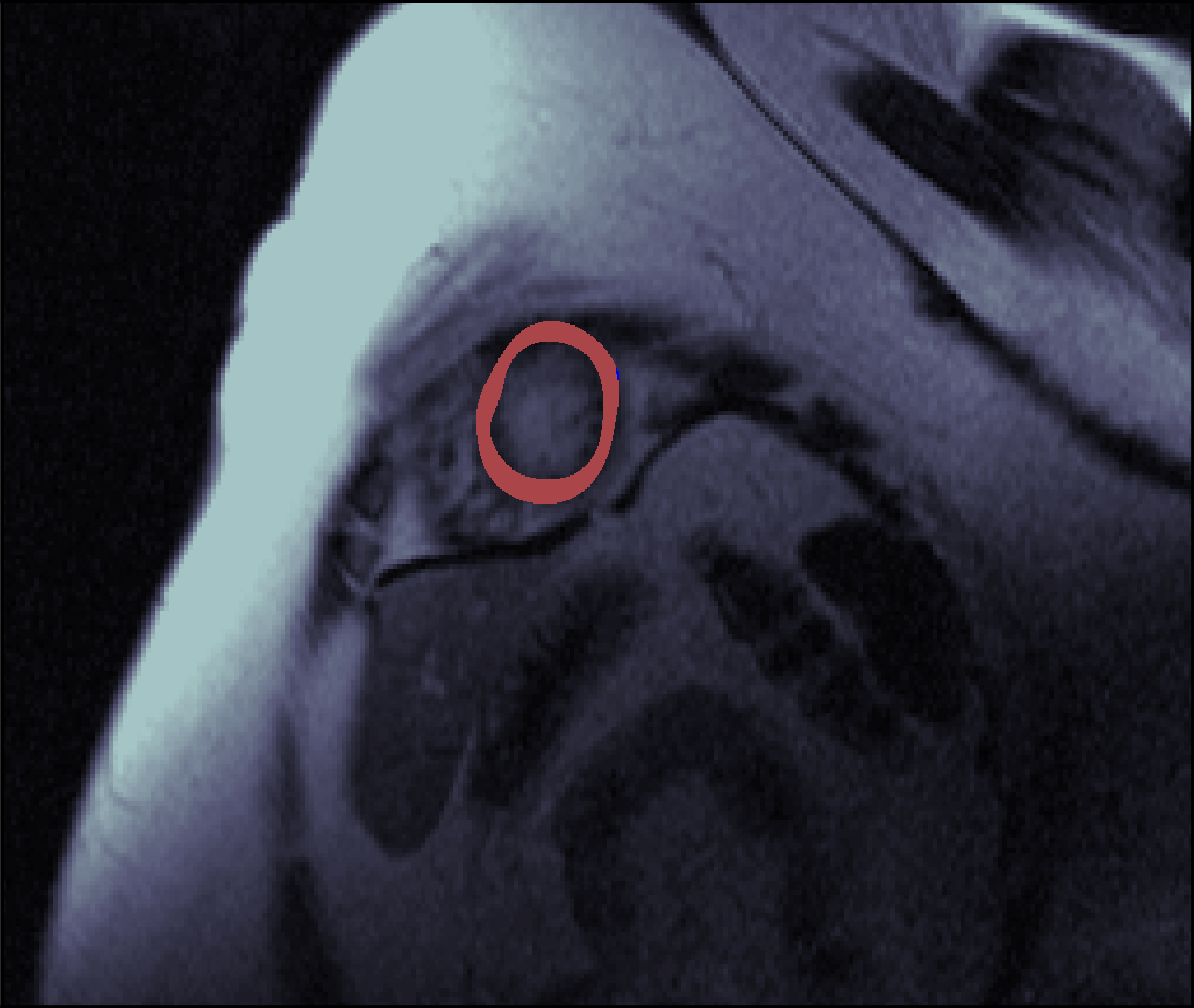

Supplement: S1 Dataset — (ZIP) [file pcbi.1007421.s001.zip › supplementary_segmented_lgemri_data/segmentations/07_04952/137_COL_20070515111245.png]

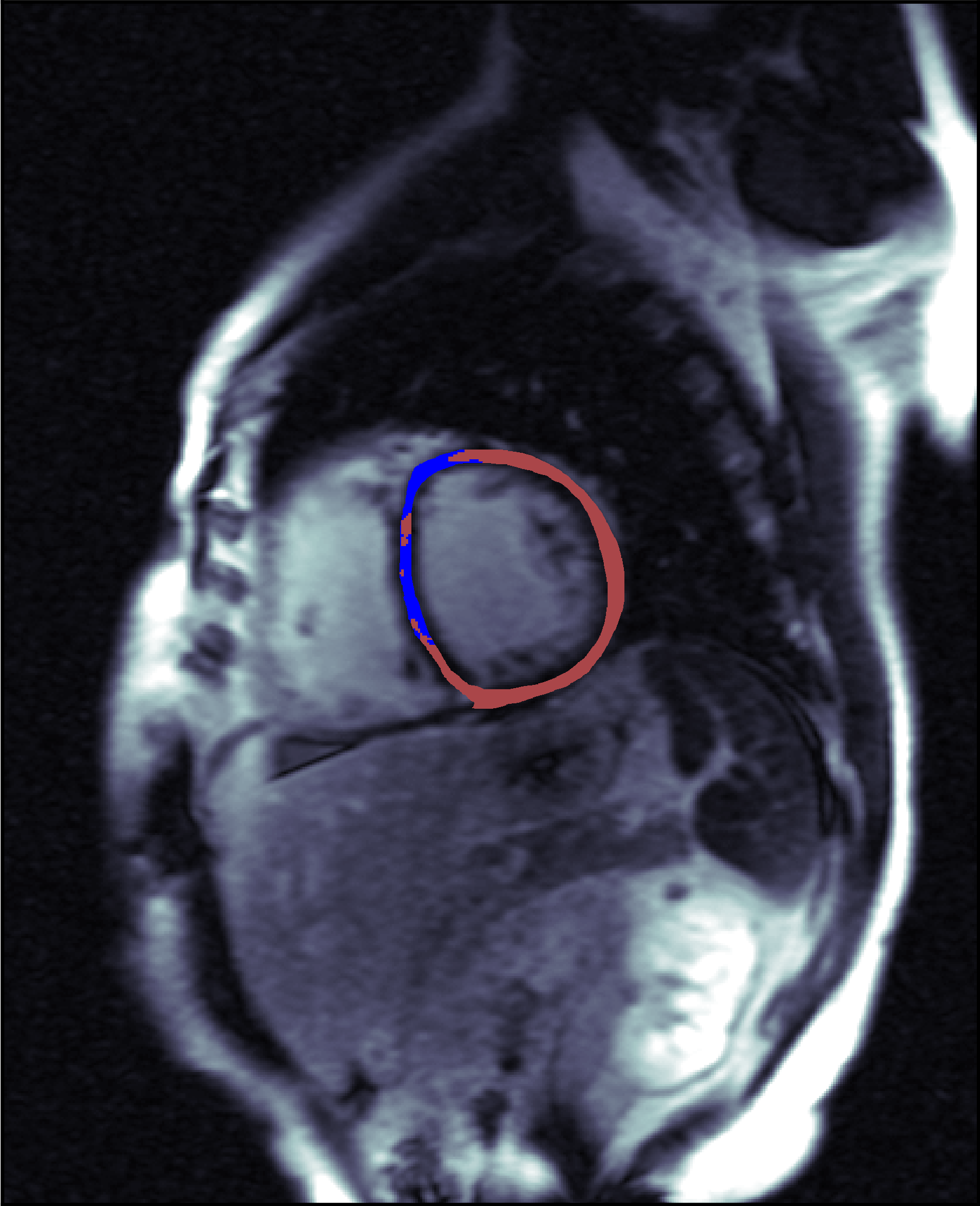

Supplement: S1 Dataset — (ZIP) [file pcbi.1007421.s001.zip › supplementary_segmented_lgemri_data/segmentations/07_01148/84_ROW_20070717091917.png]

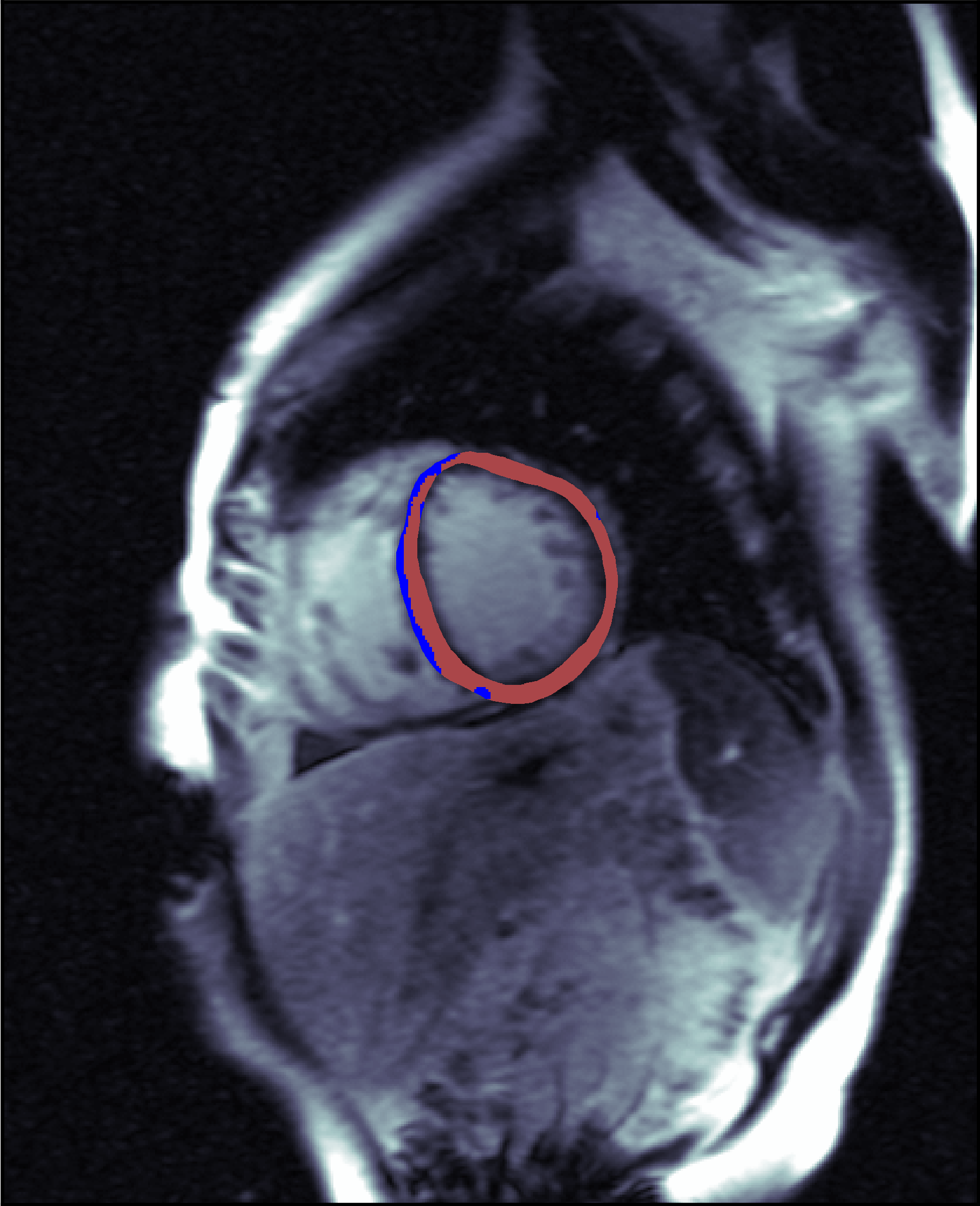

Supplement: S1 Dataset — (ZIP) [file pcbi.1007421.s001.zip › supplementary_segmented_lgemri_data/segmentations/07_01148/95_ROW_20070717092017.png]

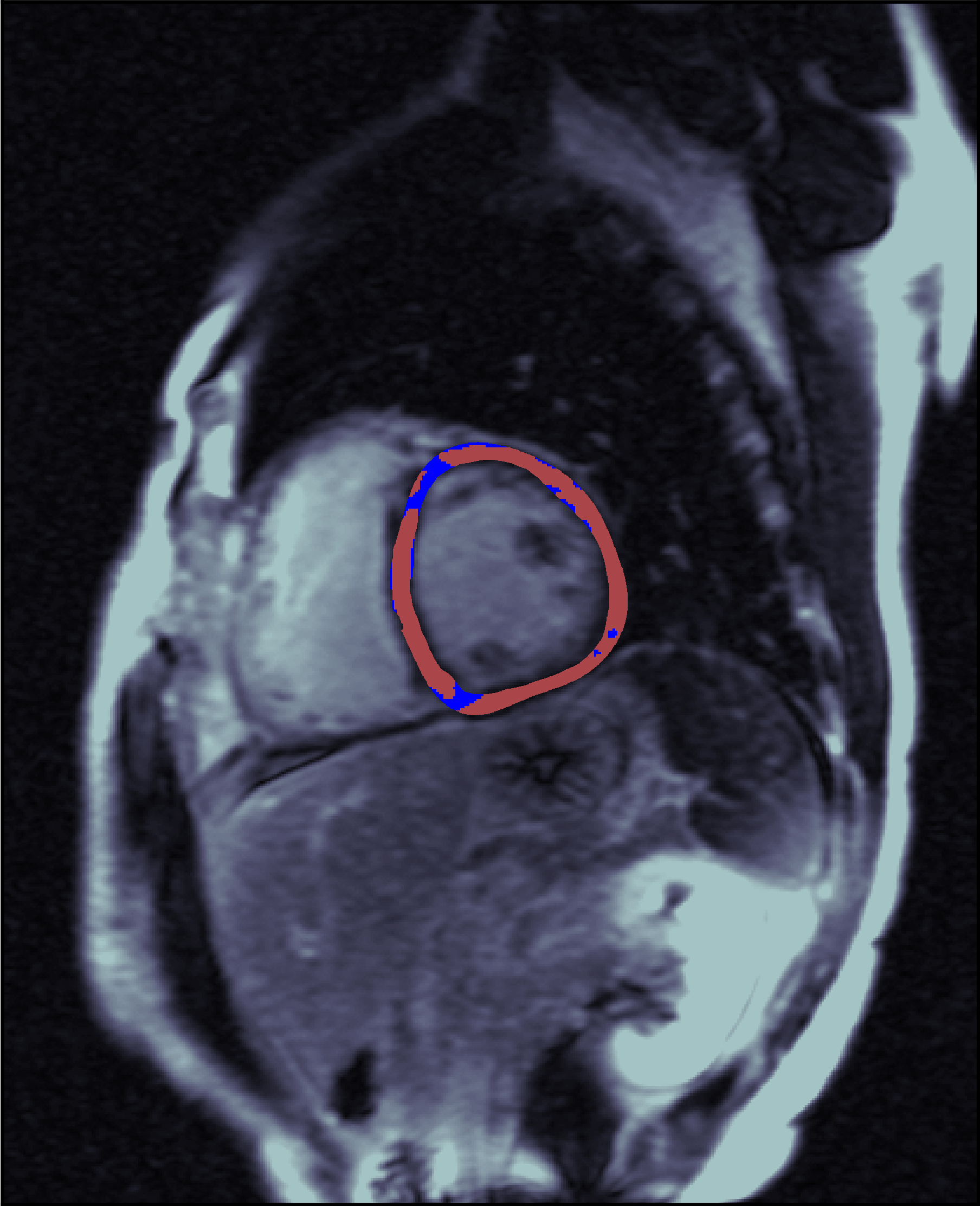

Supplement: S1 Dataset — (ZIP) [file pcbi.1007421.s001.zip › supplementary_segmented_lgemri_data/segmentations/07_01148/74_ROW_20070717091851.png]

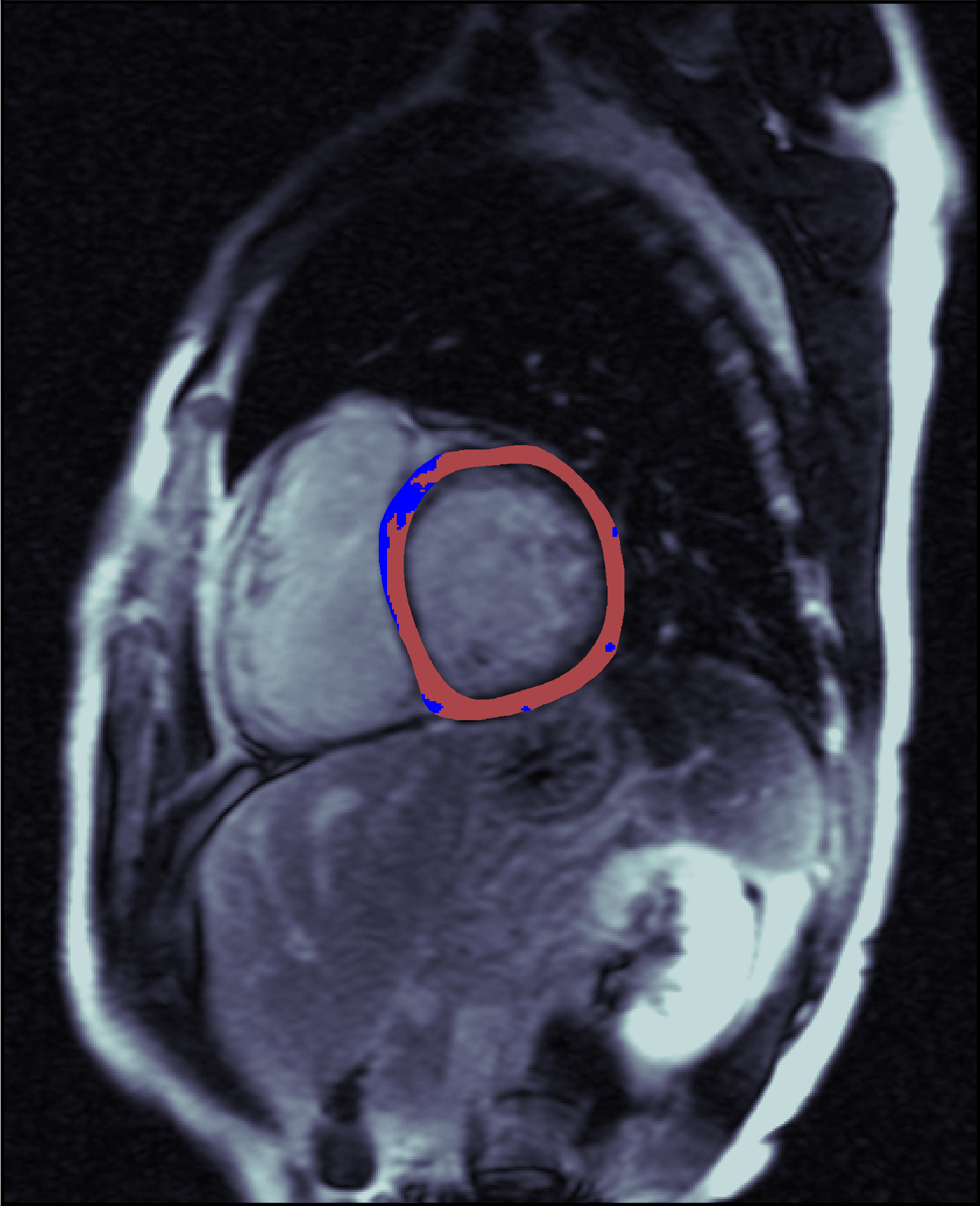

Supplement: S1 Dataset — (ZIP) [file pcbi.1007421.s001.zip › supplementary_segmented_lgemri_data/segmentations/07_01148/65_ROW_20070717091821.png]

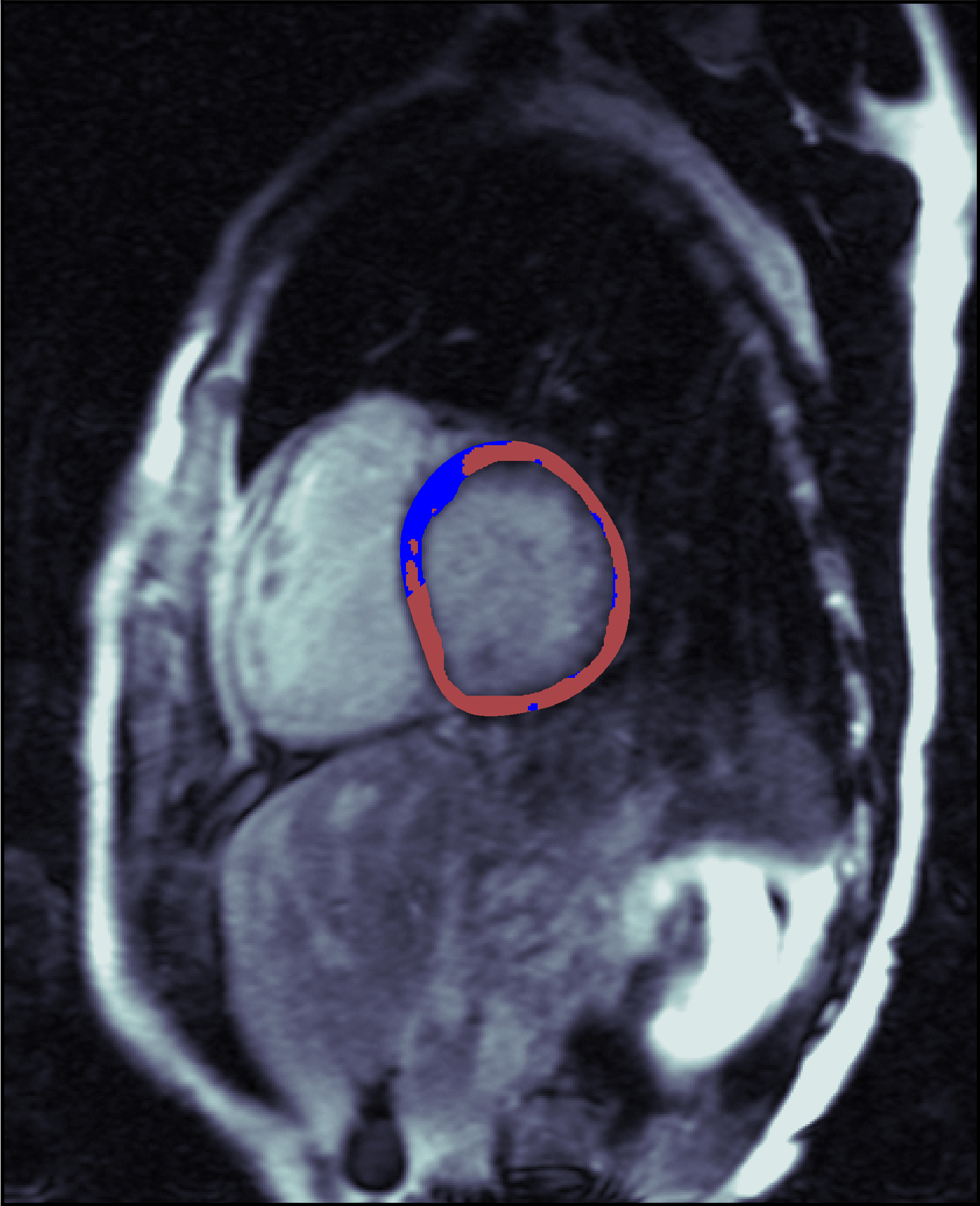

Supplement: S1 Dataset — (ZIP) [file pcbi.1007421.s001.zip › supplementary_segmented_lgemri_data/segmentations/07_01148/65_ROW_20070717091754.png]

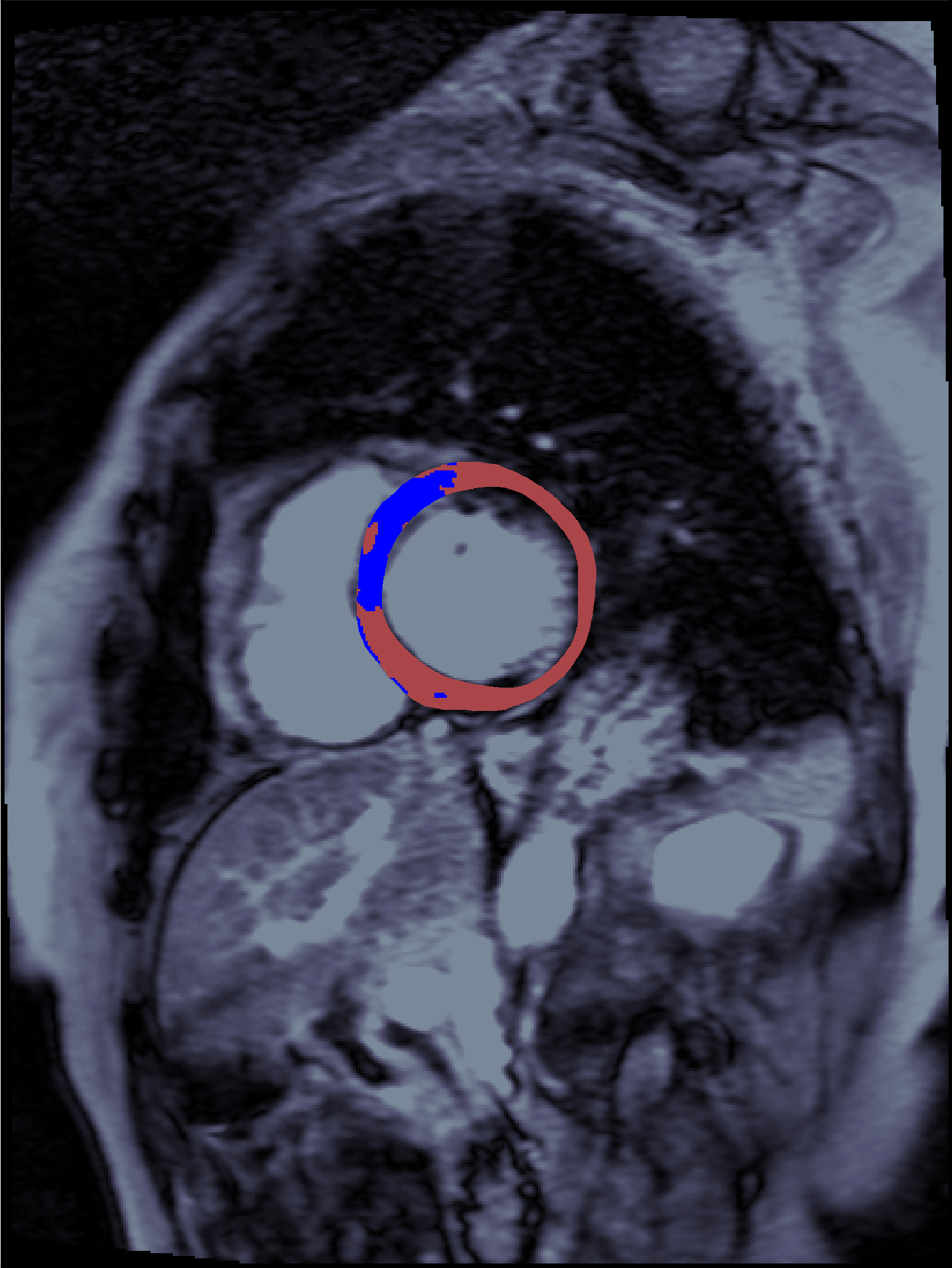

Supplement: S1 Dataset — (ZIP) [file pcbi.1007421.s001.zip › supplementary_segmented_lgemri_data/segmentations/05_23181/-36_ROW_20100713125610.png]

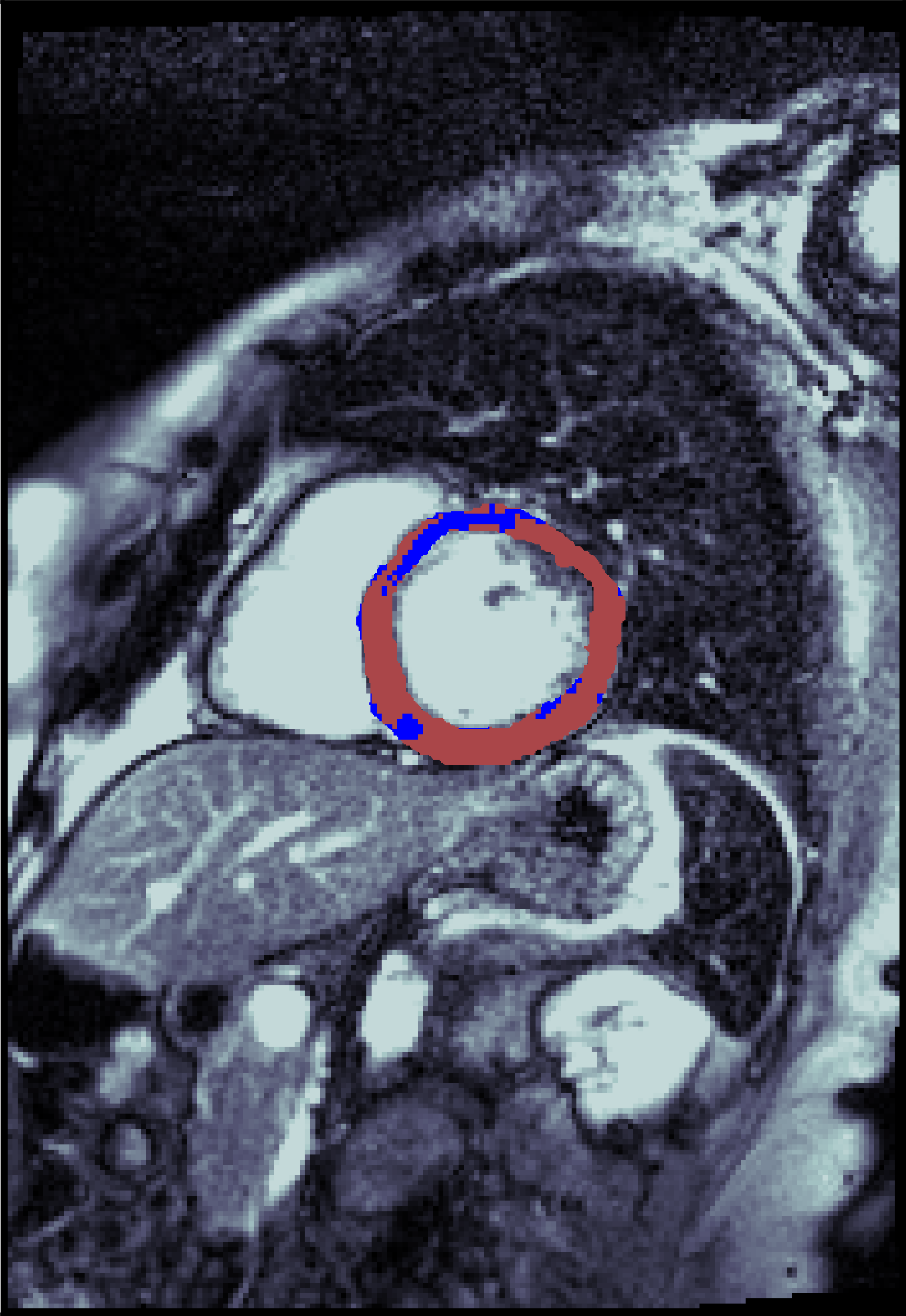

Supplement: S1 Dataset — (ZIP) [file pcbi.1007421.s001.zip › supplementary_segmented_lgemri_data/segmentations/05_23181/-45_ROW_20100713125212.png]

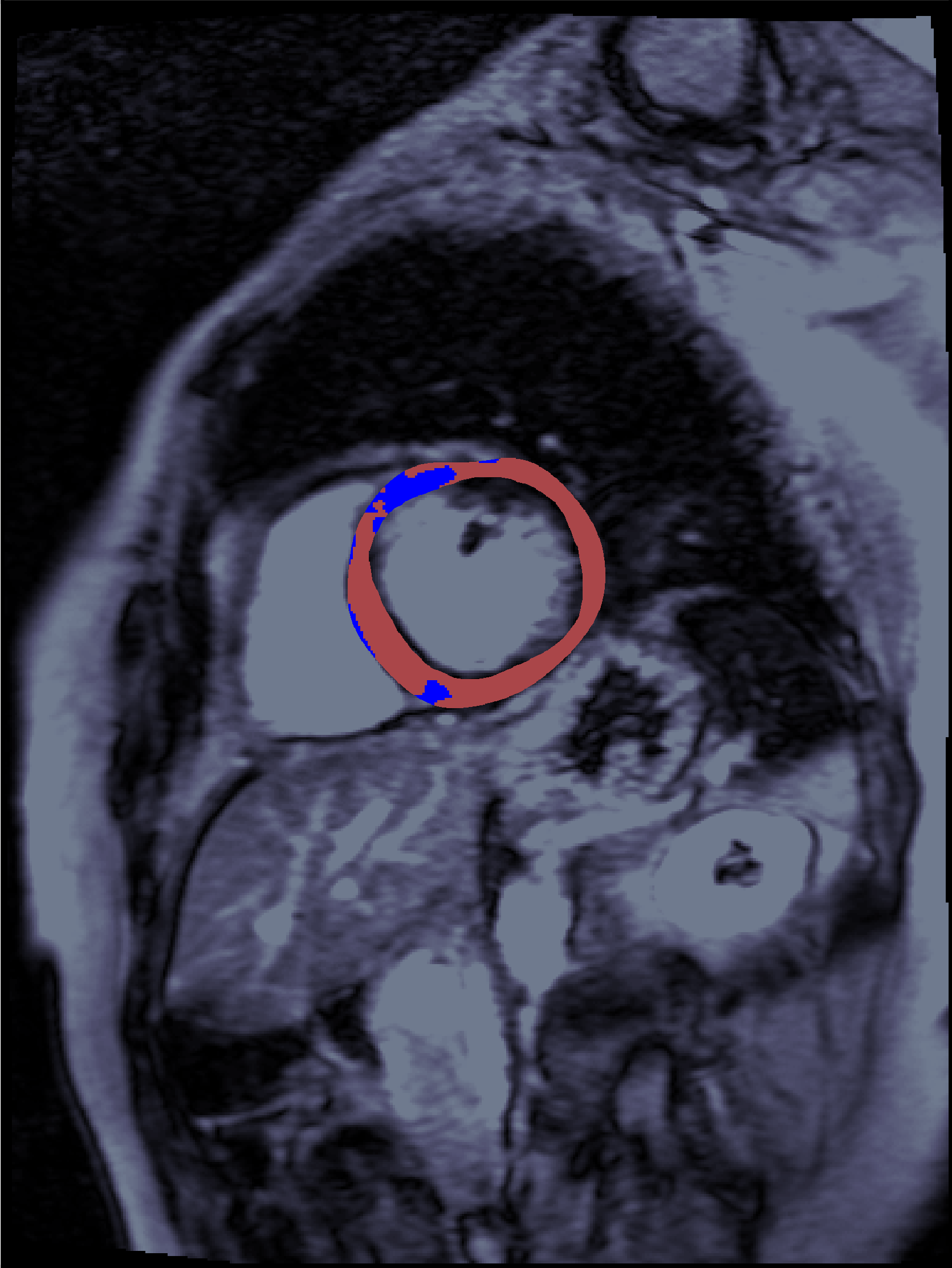

Supplement: S1 Dataset — (ZIP) [file pcbi.1007421.s001.zip › supplementary_segmented_lgemri_data/segmentations/05_23181/-46_ROW_20100713125635.png]

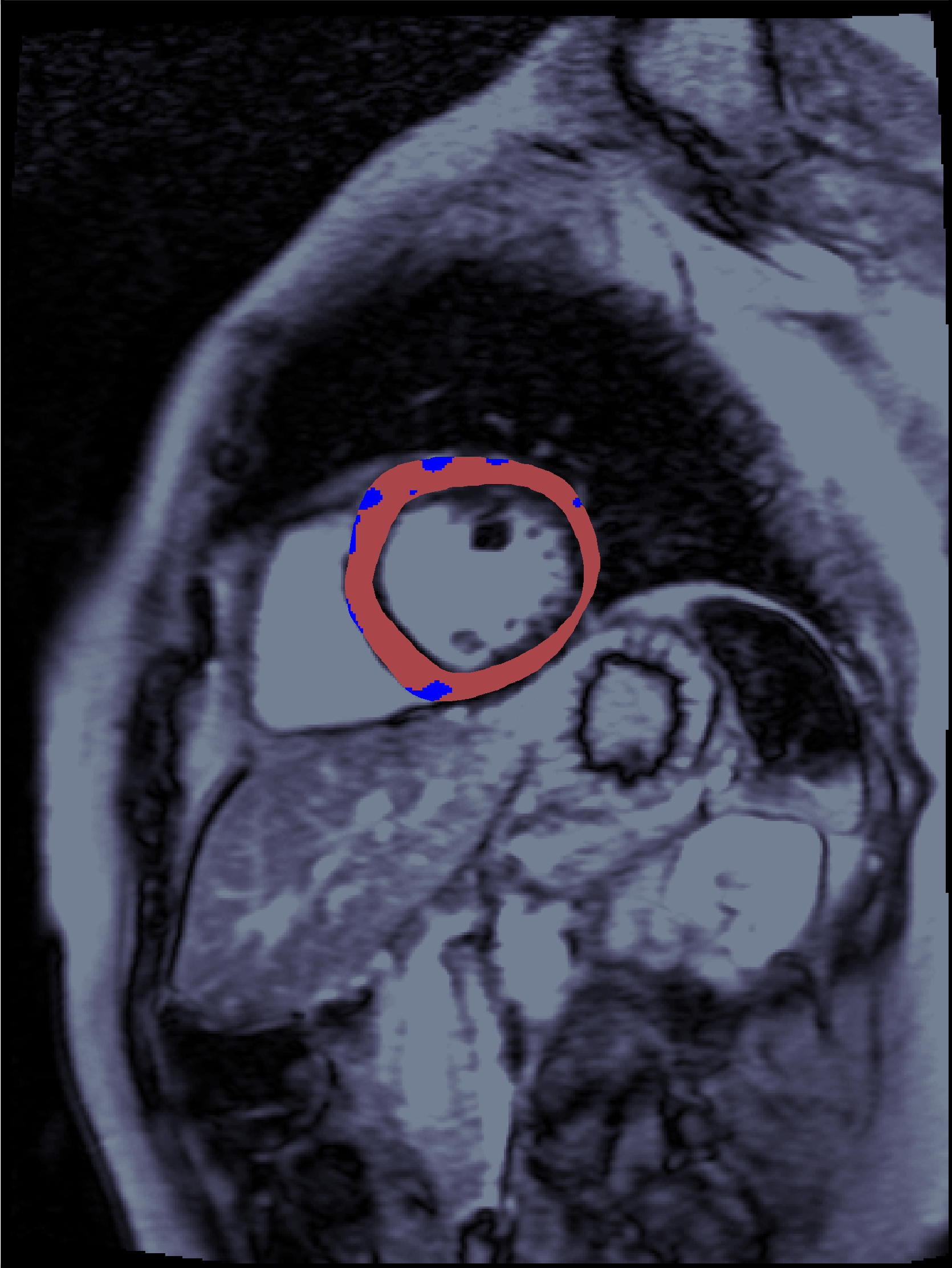

Supplement: S1 Dataset — (ZIP) [file pcbi.1007421.s001.zip › supplementary_segmented_lgemri_data/segmentations/05_23181/-56_ROW_20100713125705.png]

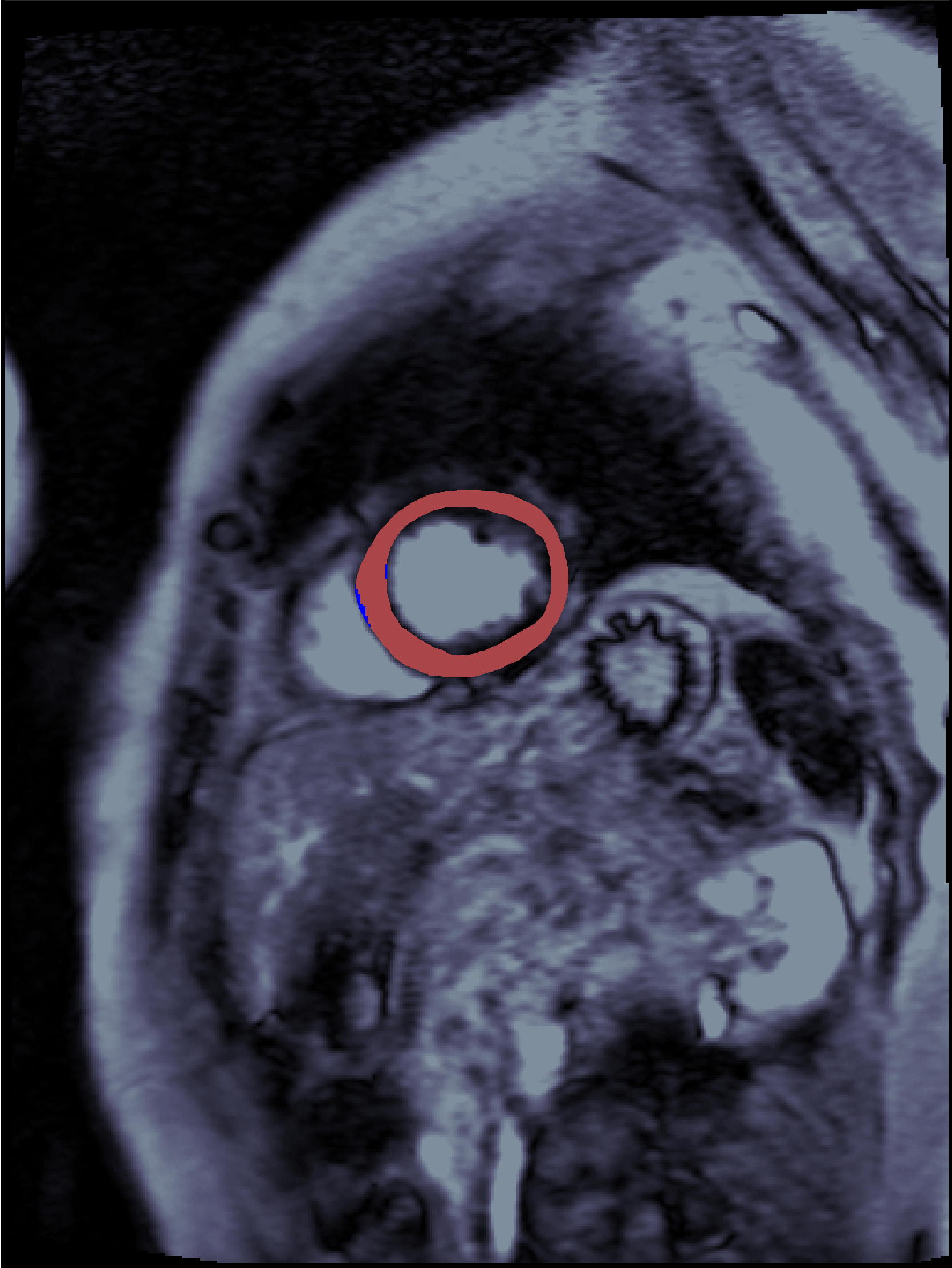

Supplement: S1 Dataset — (ZIP) [file pcbi.1007421.s001.zip › supplementary_segmented_lgemri_data/segmentations/05_23181/-76_ROW_20100713125802.png]

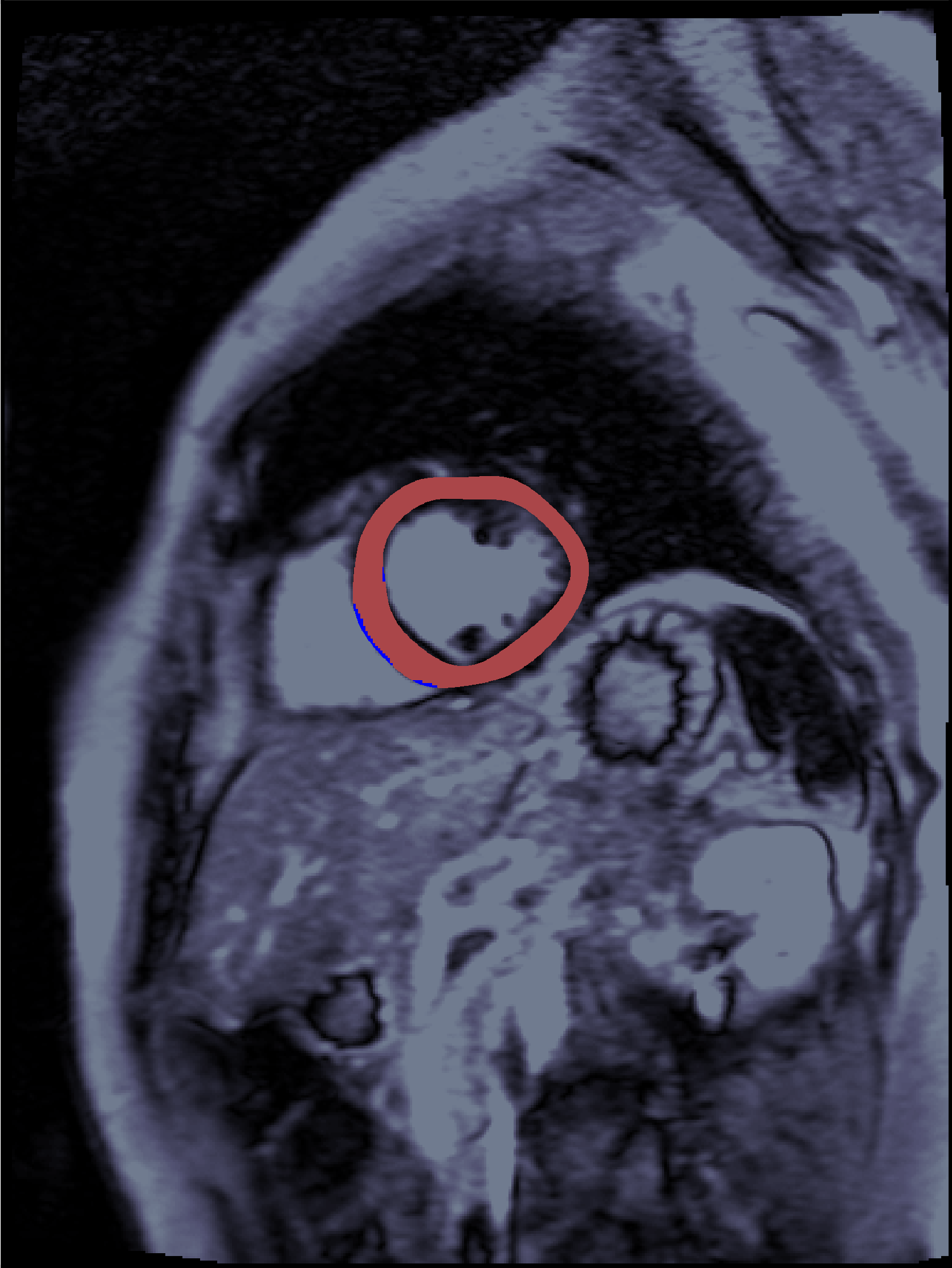

Supplement: S1 Dataset — (ZIP) [file pcbi.1007421.s001.zip › supplementary_segmented_lgemri_data/segmentations/05_23181/-66_ROW_20100713125736.png]

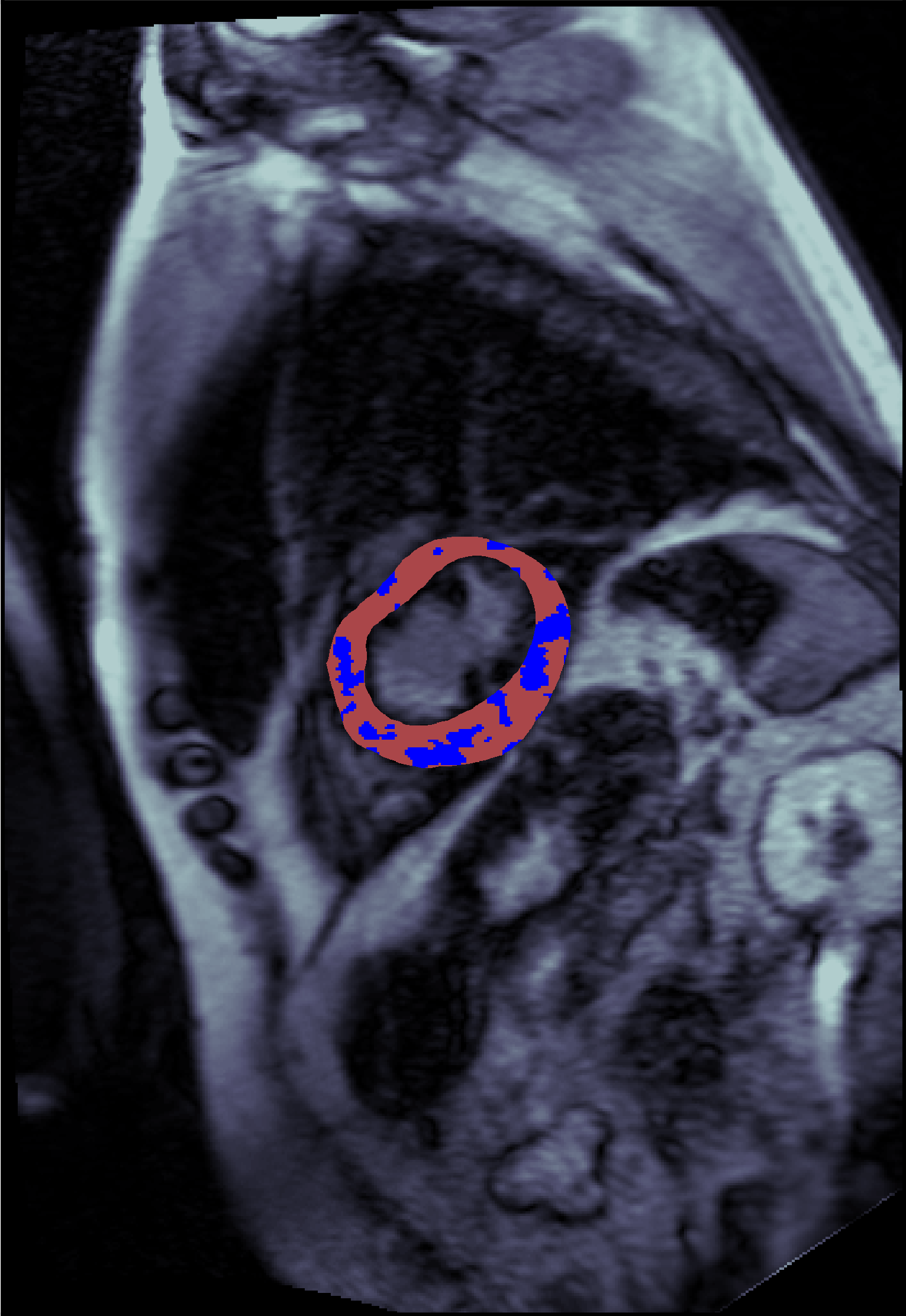

Supplement: S1 Dataset — (ZIP) [file pcbi.1007421.s001.zip › supplementary_segmented_lgemri_data/segmentations/05_14699/102_ROW_20091106154246.png]

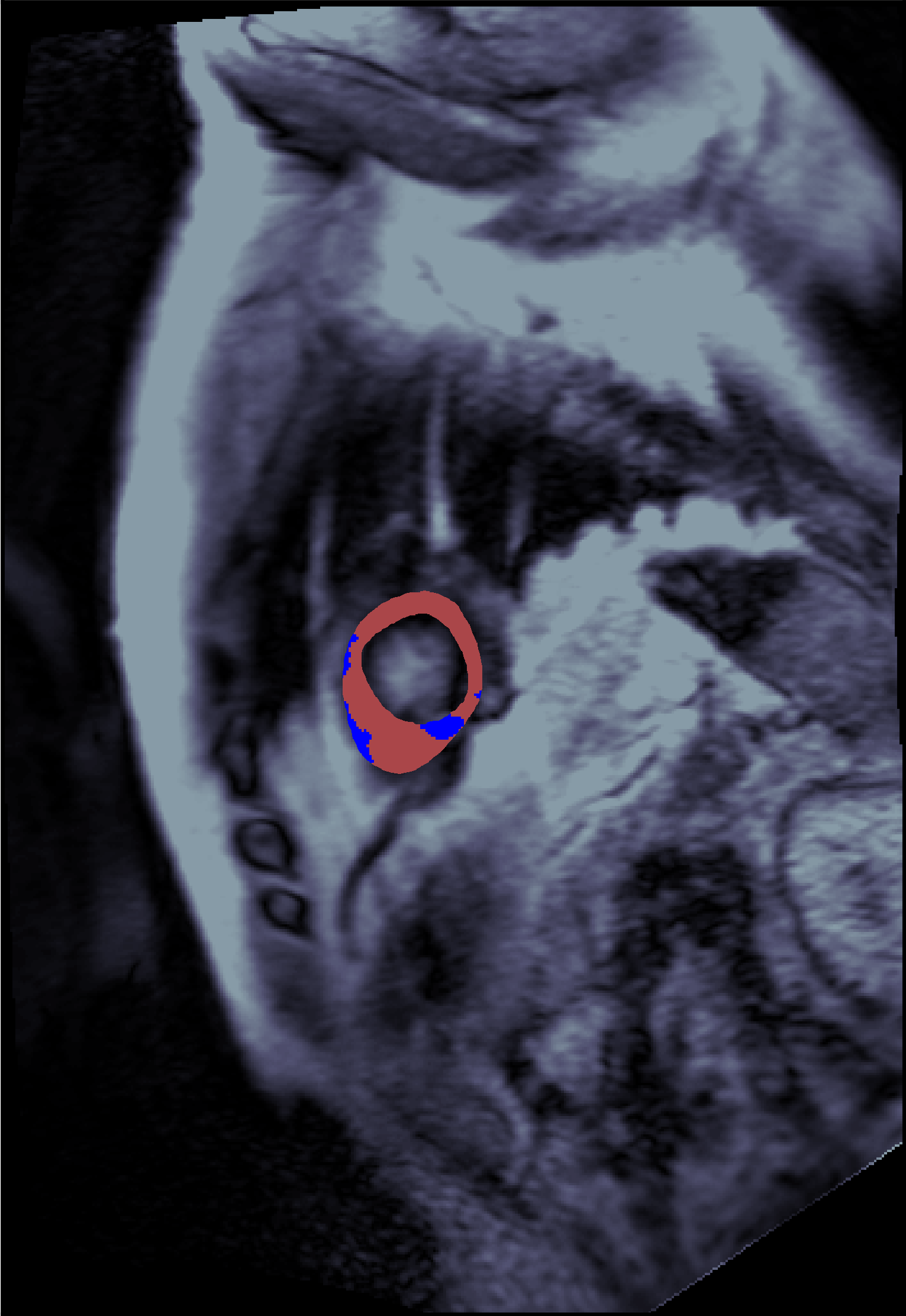

Supplement: S1 Dataset — (ZIP) [file pcbi.1007421.s001.zip › supplementary_segmented_lgemri_data/segmentations/05_14699/132_ROW_20091106154439.png]

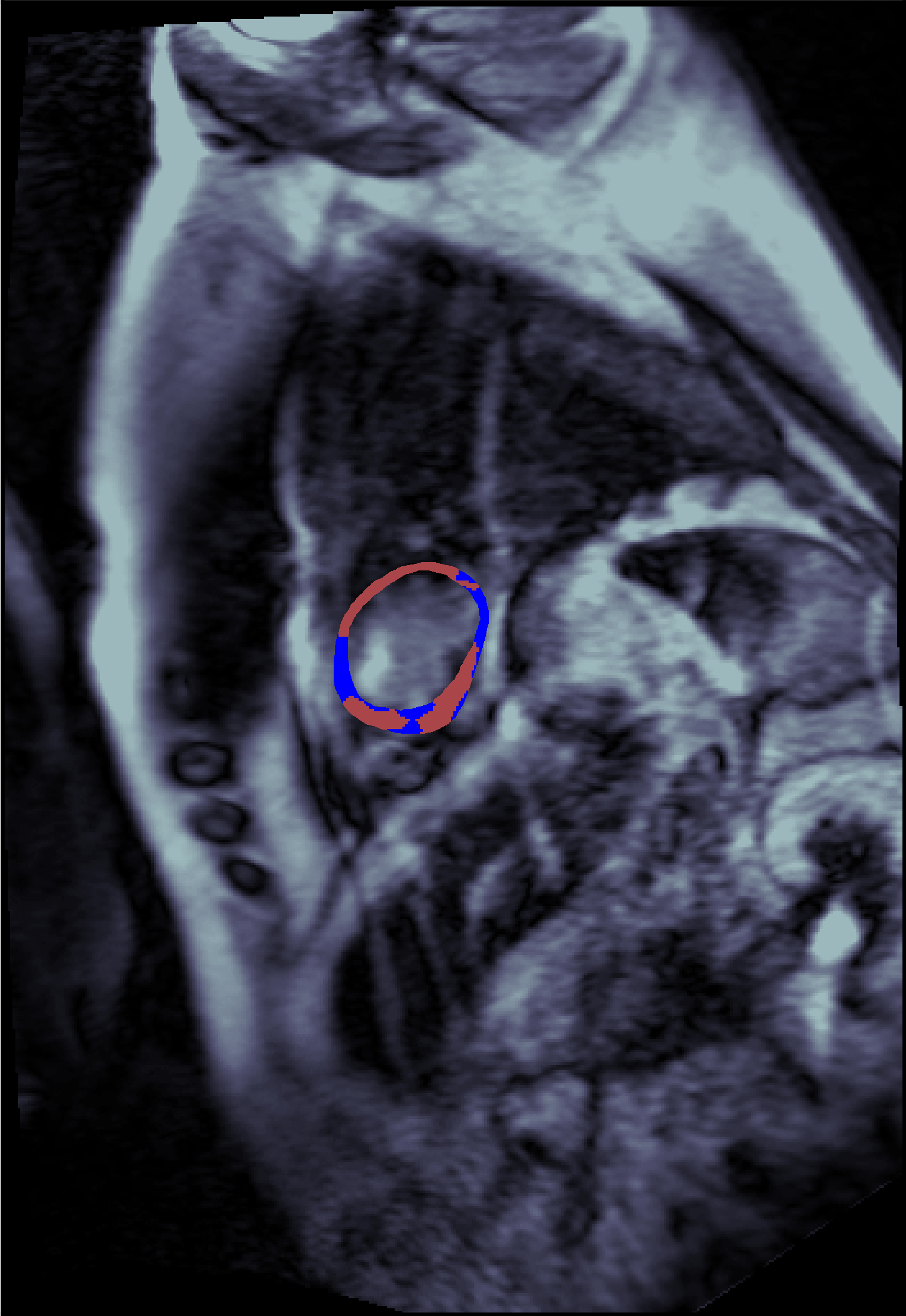

Supplement: S1 Dataset — (ZIP) [file pcbi.1007421.s001.zip › supplementary_segmented_lgemri_data/segmentations/05_14699/112_ROW_20091106154328.png]

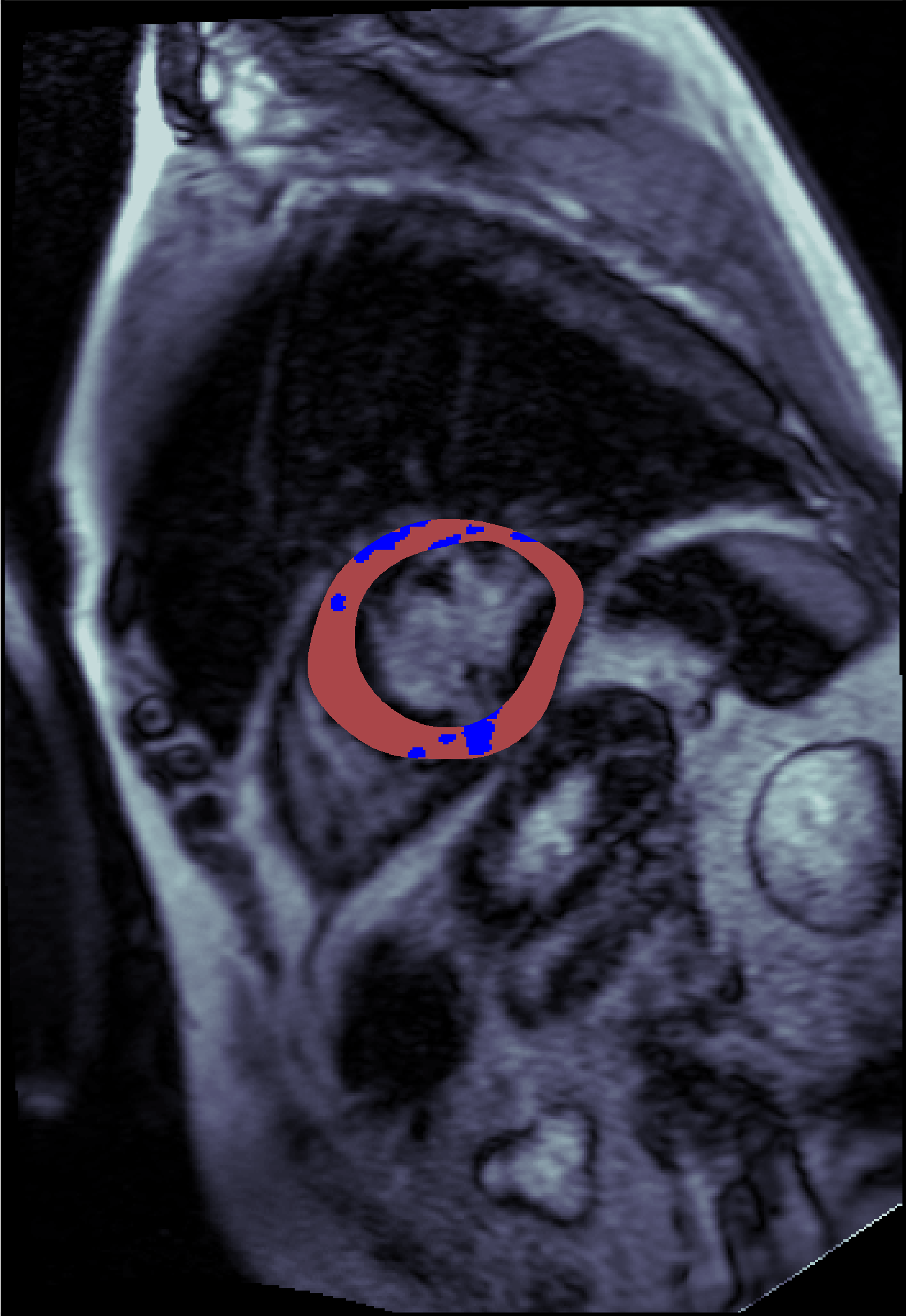

Supplement: S1 Dataset — (ZIP) [file pcbi.1007421.s001.zip › supplementary_segmented_lgemri_data/segmentations/05_14699/92_ROW_20091106154206.png]

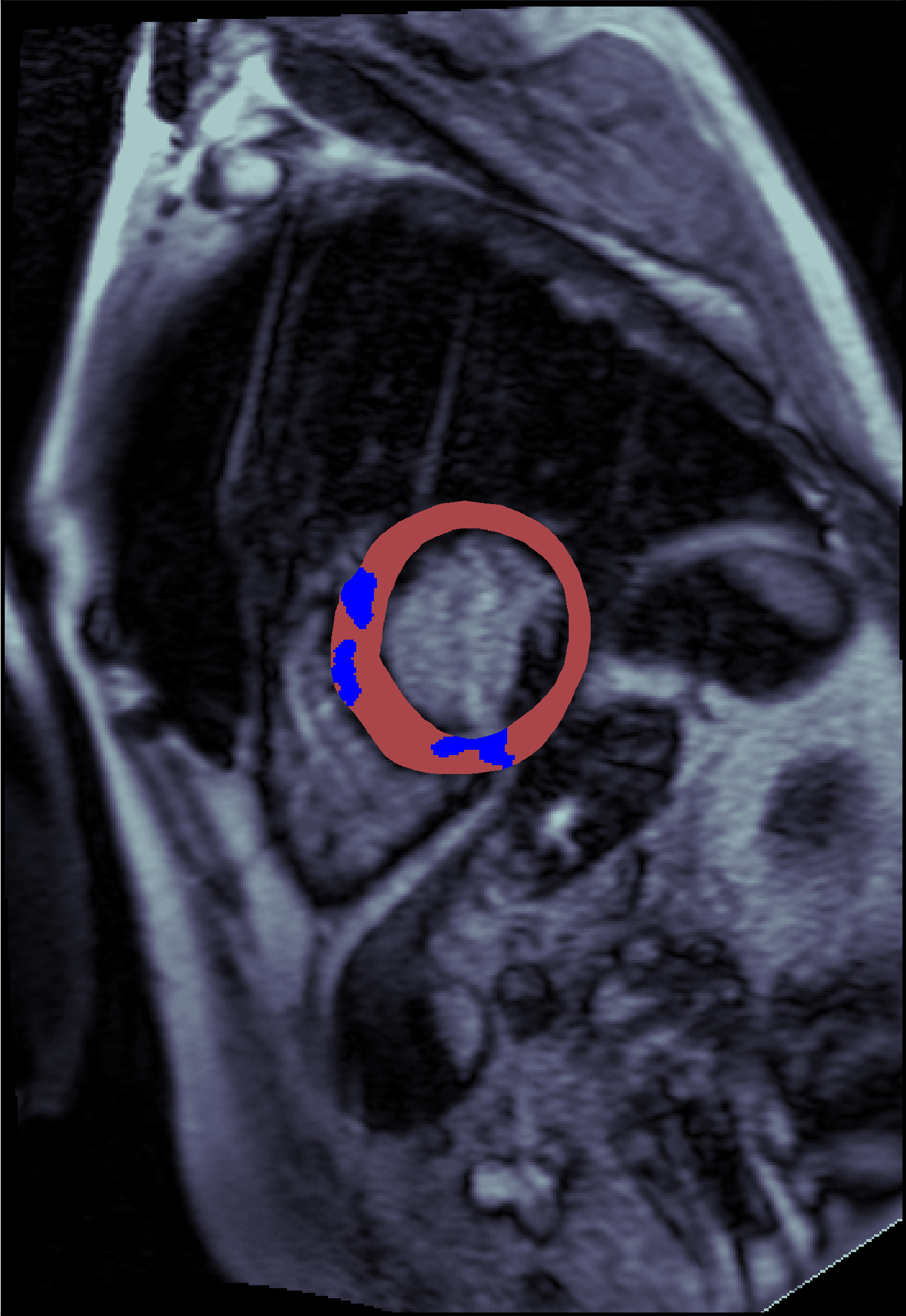

Supplement: S1 Dataset — (ZIP) [file pcbi.1007421.s001.zip › supplementary_segmented_lgemri_data/segmentations/05_14699/82_ROW_20091106154143.png]

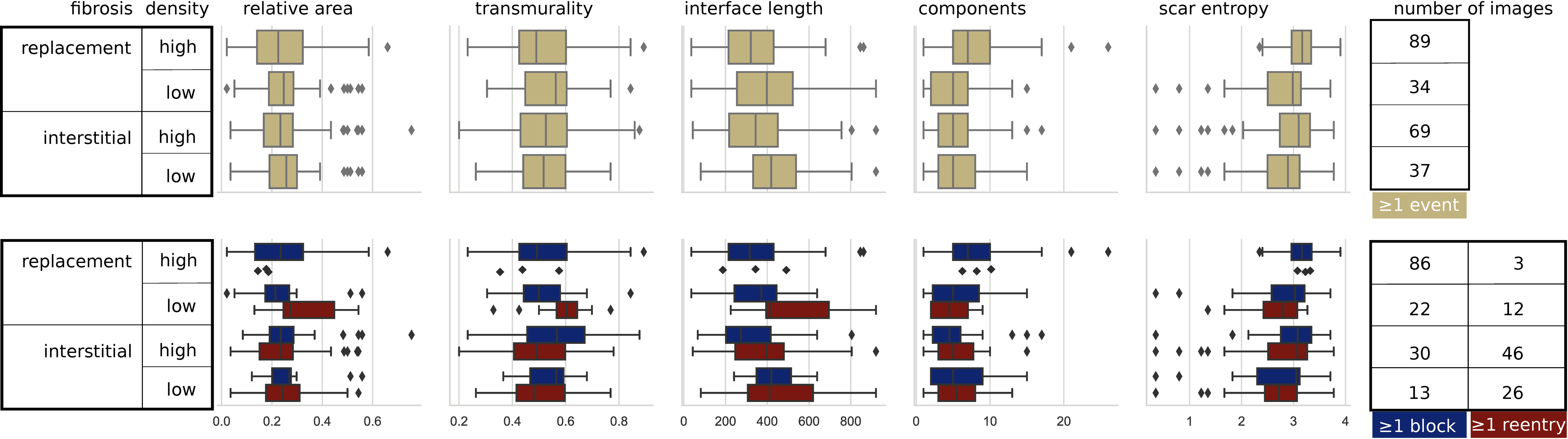

Supplement: S1 Fig — Images associated with least one reentry had a higher scar relative area (P = 0.02), higher transmurality (P = 0.02), and greater interface length (P = 0.03) than those that were associated with at least one block. For the high density interstitial microstructure, the interface length was larger (P = 0.02) and the transmurality smaller (P = 0.02), for the images associated with at least one reentry vs. those associated with at least one block. These trends, though interesting, should be viewed with caution due to the high number of comparisons (n = 20), which reduce the statistical significance after a Bonferonni correction (corrected α = 0.0025 assuming an original α = 0.05). (TIF) [file pcbi.1007421.s002.tif]

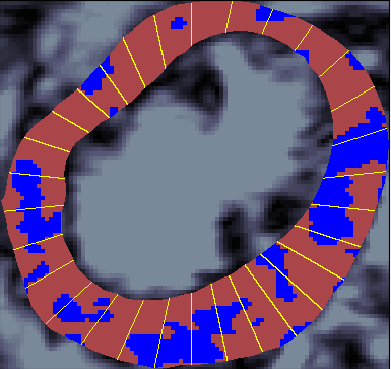

Supplement: S2 Fig — Myocardium is red, enhanced areas are blue, and rays are drawn in yellow. The ratio of enhanced to myocardial (including enchanced) pixels gives the transmurality along a ray. Note that the number of rays has been reduced to 30 in this image in order to demonstrate the method. (TIF) [file pcbi.1007421.s003.tif]
